# Identifying similar populations across independent single cell studies without data integration

**DOI:** 10.1093/nargab/lqaf042

**Published:** 2025-04-24

**Authors:** Oscar González-Velasco, Malte Simon, Rüstem Yilmaz, Rosanna Parlato, Jochen Weishaupt, Charles D Imbusch, Benedikt Brors

**Affiliations:** Division Applied Bioinformatics, German Cancer Research Center (DKFZ), 69120 Heidelberg, Germany; Division of Neurodegenerative Disorders, Department of Neurology, Medical Faculty Mannheim, Mannheim Center for Translational Neurosciences, Heidelberg University, 68167 Mannheim, Germany; Division Applied Bioinformatics, German Cancer Research Center (DKFZ), 69120 Heidelberg, Germany; Leibniz Institute for Immunotherapy, 93053 Regensburg, Germany; Division of Neurodegenerative Disorders, Department of Neurology, Medical Faculty Mannheim, Mannheim Center for Translational Neurosciences, Heidelberg University, 68167 Mannheim, Germany; Division of Neurodegenerative Disorders, Department of Neurology, Medical Faculty Mannheim, Mannheim Center for Translational Neurosciences, Heidelberg University, 68167 Mannheim, Germany; Division of Neurodegenerative Disorders, Department of Neurology, Medical Faculty Mannheim, Mannheim Center for Translational Neurosciences, Heidelberg University, 68167 Mannheim, Germany; Division Applied Bioinformatics, German Cancer Research Center (DKFZ), 69120 Heidelberg, Germany; Institute of Immunology, University Medical Center Mainz, 55131 Mainz, Germany; Research Center for Immunotherapy, University Medical Center Mainz, 55131 Mainz, Germany; Division Applied Bioinformatics, German Cancer Research Center (DKFZ), 69120 Heidelberg, Germany; German Cancer Consortium (DKTK), Core Center Heidelberg, Im Neuenheimer Feld 280, 69120 Heidelberg, Germany; Medical Faculty Heidelberg and Faculty of Biosciences, Heidelberg University, 69120 Heidelberg, Germany

## Abstract

Supervised and unsupervised methods have emerged to address the complexity of single cell data analysis in the context of large pools of independent studies. Here, we present ClusterFoldSimilarity (CFS), a novel statistical method design to quantify the similarity between cell groups across any number of independent datasets, without the need for data correction or integration. By bypassing these processes, CFS avoids the introduction of artifacts and loss of information, offering a simple, efficient, and scalable solution. This method match groups of cells that exhibit conserved phenotypes across datasets, including different tissues and species, and in a multimodal scenario, including single-cell RNA-Seq, ATAC-Seq, single-cell proteomics, or, more broadly, data exhibiting differential abundance effects among groups of cells. Additionally, CFS performs feature selection, obtaining cross-dataset markers of the similar phenotypes observed, providing an inherent interpretability of relationships between cell populations. To showcase the effectiveness of our methodology, we generated single-nuclei RNA-Seq data from the motor cortex and spinal cord of adult mice. By using CFS, we identified three distinct sub-populations of astrocytes conserved on both tissues. CFS includes various visualization methods for the interpretation of the similarity scores and similar cell populations.

## Introduction

Single-cell sequencing is a technology that captures the biomolecular status at cell resolution and measures multiple cellular markers in hundreds to millions of cells. Researchers can elucidate the genomic, epigenomic, and transcriptomic heterogeneity and variability in cellular populations and subpopulations [1].

Cell classification and labeling based on phenotypic characteristics is one of the key features of single-cell analysis, they are achieved by computationally grouping cells based on common gene expression patterns, leading to a compendium of known specific markers from previously discovered cell types, cell status (e.g. cell cycle markers), and developmental stages [2]. Cell labeling in groups is a crucial task for determining the composition and heterogeneity that helps to understand tissue composition, microenvironment function, disease, and cell fate. The available number of large-scale single-cell datasets generated using a wide range of technologies is continuously increasing with the advent of public databases [3, 4]. However, wide data integration remains difficult, because of the inherent batch-specific systematic variations and high heterogeneity of single-cell data and because of data privacy regulations to aggregate human raw data. Bioinformatic removal of batch effects can lead to the loss of biological signals owing to a general assumption of identical biological conditions [5]. Several specific tools for integrative analysis exist [6, 7]; the main challenge for combining disjoint measurements (known as diagonal integration), for both mono-omics and multi-omic data, surfaces from the distinct feature spaces.

Because of this, a general approach consists in the transformation of the input data to a shared feature space, to achieve it we can roughly classify existing methodologies in three distinct groups: label transfer, joint dimensionality reduction, and late integration. However, each approach has its own advantages and limitations, without a clearly superior method [6, 8–10]. For example, early integration using joint dimensionality reduction and late integration are better at overcoming highly heterogeneous noise, but early integration is more impacted by drop-out events, since information is locally shared on the embedding space from nearby-cells, introducing additional biases due to this sparsity [11], additionally dimensionality reduction methods are known to be prone to large information loss [12]. On the other hand, late integration can impact the power of downstream analysis (e.g. clustering), since data size is locally limited on the first steps of the analysis. Methodologically, more recent machine learning and neuronal network-based methods often lack causal modeling making the interpretation of the output difficult [10, 13].

These challenges are particularly relevant for obscured cellular populations, which are often underrepresented or highly specialized subcellular groups where robust markers have not been defined [14]. In all these cases, the inclusion of additional data and integration can improve the characterization of understudied cell groups; however, recently proposed approaches and benchmarks show moderate accuracy in cell type labeling tasks on ground-truth datasets [15, 16]. Overall, integrative methods exhibit considerable performance variability and are highly susceptible to dataset peculiarities [17]. These issues have led to increasing attention and growing concerns regarding reproducibility between single-cell studies [18–21].

Here, we present ClusterFoldSimilarity (CFS), a new methodology that effectively analyzes the similarity between groups of cells from any number of different and heterogeneous datasets without the need for batch-effect removal or integration. Our method is based on the differences in abundance of the sequenced signal between cell groups, using a Bayesian inference approach and permutation analysis to obtain the FCs and computation of similarity values based on the vector space of these differences in abundance. The underlying assumption is that the overall signal expression changes across different cell populations, subpopulations, or phenotypes in each individual dataset should be conserved in external studies with similar populations when using the same frame of reference as a comparison.

We show that our method is highly versatile and accurate and can be used to label single cell-data using reference datasets, including labeling cell types in scATAC-Seq data using scRNA-Seq or scATAC-Seq reference datasets as ground truth, with potential multimodal data analysis applications. Furthermore, we show how it can be used to compare groups of cells from different tissues and different organisms in cross-species studies, and to infer, to some extent, cell mixtures in heterogeneous clusters. Because our computational similarity method can handle any number of independent datasets, it will enable the analysis of large existing collections of single-cell studies, and it will contribute to solve the modern community efforts on building wide-scale single-cell atlases across tissues, organs, and organisms [22–24]

Finally, we exemplify how our methodology can be used to study small cell subpopulations across tissues by sequencing single-nucleus RNA-Seq from the spinal cord and motor cortex of adult mice and analyzing astrocytes as a disease-relevant glial cell subpopulation.

## Materials and methods

A typical single-cell data analysis workflow entails a series of essential steps. These steps commonly include filtering out low-quality cells and non-contributing features, normalizing and scaling the data, performing dimensionality reduction, and clustering or labeling cells based on groups defined by variability and phenotypic characteristics of interest.

Our method is designed to handle untransformed values (raw counts or peaks) and the specific groups of interest that need to be compared (e.g. clusters, cell types, cell states) in each independent dataset.

The features utilized for comparison can embody genes, transcripts, peaks, or other. Our workflow makes use of the set of features that are shared across all datasets. It is advisable to select features that exhibit some degree of variability or serve as markers of interest for our cell populations, albeit in some cases, it might be desirable to include all available features when the phenotypic characteristics are unknown, or the main interest is that of marker selection.

### Fold change distribution and pairwise computation

Fold change (FC) can be formulated as the disparity in abundance between two sample sets or groups of samples/cells, and it is one of the most ubiquitous and intuitive metrics for evaluating hypotheses in quantitative experiments. Typically, we have a set of two samples representing a specific feature abundance, these can be seen as Poisson distributed random variables with mean μ, FC is then formulated as the logarithmic ratio of the means of observed quantities such as mRNA fragments or protein levels, and statistical testing involves assessing the Null hypothesis that the measured entity exhibits no significant change in abundance under different conditions. Various statistical methods have been used and developed to calculate a *P* value associated with this measure of differential expression. However, caution must be exercised when interpreting measurements due to inherent technical biases and stochasticity of biological data. This concern is particularly pertinent when dealing with highly sparse single-cell data, where low or zero abundance observations may yield infinite or extremely high FCs.

To mitigate this problem several approaches have been proposed, being the most widely used the addition of a small positive number p (often set as *p*= 1) to the count data, known as a pseudocount. With this approach, problems like division by 0, or infinite ${{C}_n}$ values for log-normalization are avoided. But, assigning arbitrary numbers to p has a strong influence on the difference in abundance of low expressing entities: this is because artificially inflating the counts make disproportionate increase in low expressed features (for a feature with 1 count, adding a *p*= 1 corresponds to a 100% increment, meanwhile a feature with expression 1000, a *p*= 1 yields an increment in size of just a 0.1%). This has been demonstrated as problematic, both for differential expression and downstream analysis [25–28].

Here we extend the methodology devised by Erhard and Zimmer [28, 29] which employs an empirical Bayes procedure to estimate pseudocounts and FCs. We have made certain modifications to this approach (see details). We selected a FC estimation procedure for several reasons: our similarity score is based on cumulative changes in abundances from a frame of reference (i.e. specific cluster or cell population), rather than the statistical significance of those changes. Consequently, we bypass the computation of statistical scores or P values. Furthermore, considering the large number of pairwise tests required to assess differential abundance and compute our similarity score, utilizing regression models to estimate size effects would incur in excessive computational time.

To calculate the pseudocounts for each group, we estimate the prior distribution based in the observed FCs without pseudocounts by calculating:


(1) \begin{eqnarray*}
{\mathrm{FC}}\left( {{{{\mathrm{C}}}_{\mathrm{n}}}{{{\mathrm{C}}}_{\mathrm{m}}}} \right) = {\mathrm{log}}\left( {{{{\mathrm{C}}}_{\mathrm{n}}}} \right) - {\mathrm{log}}\left( {{{{\mathrm{C}}}_{\mathrm{m}}}} \right)
\end{eqnarray*}

where ${{C}_n}$ and ${{C}_m}$ are, respectively, the mean of feature i expression in groups n and m. Consequently, we compute the mean $\mu$ and variance ${{\sigma }^2}$ of the prior as:


(2) \begin{eqnarray*}
\mu = {\mathrm{mean}}\left( {FC\left( {{{C}_n}{{C}_m}} \right)} \right)
\end{eqnarray*}

(3) \begin{eqnarray*}
{{\sigma }^2} = {\mathrm{max}}{{\left( {q\left( {FC\left( {{{C}_n}{{C}_m}} \right),0.84} \right) - \mu ,\mu - q\left( {FC\left( {{{C}_n}{{C}_m}} \right),0.15} \right)} \right)}^2}\nonumber\\
\end{eqnarray*}

These quantiles correspond to one standard deviation for a Normal distribution. Importantly, to construct this prior distribution, we only use informative features, i.e. entries with non-zero values in both groups. Additionally, we impose a minimum of three features for calculating the prior, as opposed in the original [28] where 0 values are allowed on one group and no minimum number of informative features is required. If there are not sufficient values, we compute the FCs adding a pseudocount p (equation 4) to ${{C}_n}$ and ${{C}_m}$ for each gene, based on the hyperbolic arcsine (asinh) function with no log transformation, and we compute the variance using the standard formula. The asinh function has been shown to have desirable properties as pseudocount estimator [27, 30], as it avoids preselecting an arbitrary constant, and yields a proportional pseudocount for each individual gene.


(4) \begin{eqnarray*}
{{p}_{ij}} = \sqrt {{{{\left( {C{\mathrm{|}}ij} \right)}}^2} + 1} \ {\mathrm{for\ sample\ i}},{\mathrm{gene\ j}}
\end{eqnarray*}

Finally, we select the mean instead of the median as the $\mu$ prior to reflect more extreme values in low abundance observations. In summary, we believe these changes are relevant when dealing with single-cell data, especially in the case of ATAC-Seq, single-nuclei RNA-Seq, and single-cell proteomics. As mentioned, ${{C}_n}$ and ${{C}_m}$ are expected to follow a Poisson distribution, being the conjugated prior of this distribution the gamma distribution. Following previous statistical frameworks [28], the expected mean and variance of the log distribution can be formulated as:


(5) \begin{eqnarray*}
\frac{{\psi \left( \alpha \right) - \psi \left( \beta \right)}}{{log2}} = \mu
\end{eqnarray*}

(6) \begin{eqnarray*}
\frac{{{{\psi }_1}\left( \alpha \right) + {{\psi }_1}\left( \beta \right)}}{{{\mathrm{lo}}{{{\mathrm{g}}}^2}2}} = {{\sigma }^2}
\end{eqnarray*}

And thus, pseudocounts $\alpha$ and $\beta$ can be computed by minimizing the function:


(7) \begin{eqnarray*}
{{\left( {\psi \left( \alpha \right) - \psi \left( \beta \right) - \mu } \right)}^2} + {{\left( {{{\psi }_1}\left( \alpha \right) + {{\psi }_1}\left( \beta \right) - {{\sigma }^2}} \right)}^2}
\end{eqnarray*}

Where $\psi$ and ${{\psi }_1}$ are the digamma and trigamma functions, respectively. Finally, the FC distribution can be estimated using the new pseudocounts $\alpha$ and $\beta$ for each feature as:


(8) \begin{eqnarray*}
FC\left( {{{C}_n}{{C}_m}} \right) = \frac{{\psi \left( {{{C}_n} + \alpha } \right) - \psi \left( {{{C}_m} + \beta } \right)}}{{{\mathrm{log}}2}}
\end{eqnarray*}

We apply the median to zero procedure [31] as a normalization factor over the new distribution of FCs. This estimated FC is computed between cells in cluster/group i and cells in cluster/group j $\forall j\neq i$. In such manner, we obtain k-1 FCs (k: number of clusters/groups in the dataset) for each feature (e.g. a dataset with 10 clusters/groups will yield 9 FC values for each feature on each of the 10 clusters/groups). By doing this, we obtain a unique frame of reference for each cell group that encompass all the abundance differences from the perspective of each individual cell group, this information can be later compared with another external differential abundance reference, in our case, a cluster or group of cells coming from an independent dataset.

### Cell subsampling and FC stabilization

As highlighted in preceding sections, a primary obstacle in calculating differential abundance arises from the occurrence of low to zero values, which can result in infinite or excessively high FCs that do not accurately represent genuine biological effects. To mitigate this, we employ a permutation-based methodology to estimate the null distribution of the differences in abundance. Here, we randomly subsample 1/3 of the cells from each group “n” times and then estimate the FCs utilizing the Bayesian approach (including the pseudocount estimation) described in the previous section:


(9) \begin{eqnarray*}
FC\left( {{{C}_n}{{C}_m}} \right) = \frac{{\mathop \sum \nolimits_{j = 1}^n FC{{{\left( {{{C}_n}{{C}_m}} \right)}}_{{\mathrm{subsampl}}{{{\mathrm{e}}}_{\mathrm{j}}}}}}}{n}
\end{eqnarray*}

By subsampling 1/3 of the cells in each draw, the average number of permutations needed to observe all n cells in a group is:


(10) \begin{eqnarray*}
{\mathrm{permutations}} = \left( {\mathop \sum \limits_{j = 1}^n \frac{1}{{j + 1}}} \right)*3
\end{eqnarray*}

Which yields on average feasible computational times. As a reference, it is needed an average of 16 permutations to observe all cells in a group of 100 cells (Supplementary Fig. S1C).

Overall, we show (Supplementary Fig. S1A and D) that subsampling cells and estimating n times the FC posterior distribution using a Bayesian procedure shrinks the mean of estimated FCs -coming from low count data and with no real and significant difference in abundance towards zero, meanwhile maintaining the FC distribution for significant values (Supplementary Fig. S1B and E). This is expected for a normal random distribution with mean $\mu = 0$. By contrast, features with available expression data and true FC maintain the same effect size throughout the n permutations.

### Similarity score

We can think of FCs as unit vectors, being the magnitude equal to the FC value and direction equal to the sign of the expression change, the element-wise product of the magnitudes of these two vectors gives as a result a perpendicular vector computed as:


(11) \begin{eqnarray*}
C = F{{C}_a} \cdot F{{C}_b}
\end{eqnarray*}

Following this approach, the direction of this perpendicular vector will act as a concordance metric of the direction of the FCs (perpendicular vectors with negative component will indicate non-concordance in the differences in abundance of two frames of reference: the FCs have opposite directions). The FCs are computed using the method described in the previous section, and it is calculated pairwise between each cluster i and all the rest of the clusters j belonging to the same dataset.

Once all frames of reference are computed (all the possible combinations of FCs between all clusters/groups from each independent dataset), we start by comparing the cluster 1 from dataset n with the cluster 1 in dataset m and continue iteratively. We achieve this comparison by computing, for each feature, the Hadamard product of all pair-wise combinations (equation 11). We denominate ${{S}_i}$ these collections of products for each specific feature i and every combination of FCs for the two clusters being compared$,{\mathrm{\ }}$being the number of possible combinations $k$:


(12) \begin{eqnarray*}
k = \mathop \prod \limits_{i = 1}^n \left( {{{k}_i} - 1} \right)
\end{eqnarray*}

Being ${{k}_i}$: the number of groups in dataset i. Then we calculate for each feature the mean of these k perpendicular unit vectors, that we denominate SC (mean of feature-wise products from equation 11).


(13) \begin{eqnarray*}
S{{C}_{{\mathrm{featur}}{{{\mathrm{e}}}_{\mathrm{j}}}}} = \frac{{\mathop \sum \nolimits_{i = 1}^k {{S}_i}}}{k}{\mathrm{\ where\ }}{{{\mathrm{S}}}_{\mathrm{i}}}{\mathrm{\ is\ the\ product\ of\ fold - changes}}\nonumber\\
\end{eqnarray*}

The assumption that we build upon is that two groups (e.g. clusters) resembling the same cell population will have distinct markers (either positive or negative), and thus FC for this marker must show similar size effects across independent datasets yielding a positive SC. This SC value will additionally serve as a measure of feature importance, being the feature with the highest positive value the one that contributes the most to the similarity between the two clusters (negative values of the pair-wise products indicate opposite FC values, and thus, dissimilarities between the changes in expression of the cell groups being compared). As these scores are built from differences in abundance, these selected contributing features can be seen as expression markers for both cell groups tied together by the similarity score.

An additional weight $\omega$ is added based on the concordance of the number of positive vs negative SC values (only values with absolute SC > 0.2 are considered), this assures to give more weight or penalize the similarity values considering the concordance of the direction of expression changes across datasets, especially for low FCs (a set of small negative SCs may add a small quantity to the overall similarity, but if the number of negative SCs is much larger than positive SCs this will indicate and overall tendency of non-concordant changes in abundances). Weight $\omega$ is calculated as:


(14) \begin{eqnarray*}
\omega = \log \left( {\left| {{\mathrm{N}}.{\mathrm{PO}}{{{\mathrm{S}}}_{{\mathrm{SC}}}} - {\mathrm{N}}.{\mathrm{NE}}{{{\mathrm{G}}}_{{\mathrm{SC}}}}} \right|} \right){\mathrm{*SIGN}}\left( {{\mathrm{N}}.{\mathrm{PO}}{{{\mathrm{S}}}_{{\mathrm{SC}}}}- {\mathrm{N}}.{\mathrm{NE}}{{{\mathrm{G}}}_{{\mathrm{SC}}}}} \right)\nonumber\\
\end{eqnarray*}

Finally, the similarity value for a pair of clusters is calculated as the square root of the sum (absolute value) of the SC values from all features, retaining the sign of the sum:


(15) \begin{eqnarray*}
{\mathrm{similarity}} = \left( {\sqrt {\left| {\mathop \sum \limits_{j = 1}^n S{{C}_{{\mathrm{featur}}{{{\mathrm{e}}}_{\mathrm{j}}}}}} \right|} + \omega } \right)*{\mathrm{SIGN}}\left( {\mathop \sum \limits_{j = 1}^n S{{C}_{{\mathrm{featur}}{{{\mathrm{e}}}_{\mathrm{j}}}}}} \right)\nonumber\\
\end{eqnarray*}

The highest similarity positive value calculated this way will correspond with the most similar cluster: it is expected that, for a specific population of cells, overall changes in gene expression across different clusters (other cell populations) are conserved on different datasets when using a similar frame of comparison, and thus the sign of the product of FCs should be positive and its module higher than non-concordant groups of cells.

### Interpretation of the similarity scores

The similarity scores computed in the previous section have either positive or negative values, indicating correlations in gene expression: it compares a cell population against the remainder of the dataset populations, and computes the similarity between another cell population from an external dataset and their respective local cells. Given the inherent diversity and complexity of single-cell data, as well as variations in methods for grouping cells into populations (e.g. clusters, cell states, experimental conditions, or pre-existing annotations), the interpretation of these scores is highly context-dependent.

A positive similarity score suggests that the same markers are identified in populations derived from different datasets. This outcome may indicate that the populations consist of the same cell type, a similar mixture of cell types, or closely related lineages. It may also reflect shared conditions or cell states. Conversely, a negative score indicates anticorrelated expression changes between two independent populations; for example, a marker that is upregulated in one dataset may be downregulated in another. The interpretation of such negative scores depends, again, on the context. For instance, if a specific cell type is absent or is not expected in one dataset, similarity scores for the former population in the other dataset may be negative or near zero. However, if a closely related cell type exists (e.g. within the same lineage), the score may be positive. Negative values may also highlight differences between the same cell type under varying conditions or states. In all cases, a detailed analysis of the markers is essential to draw meaningful conclusions from the similarity scores, and the computed similarity graph as well as the community analysis can help to understand these complex relationships.

### Similarity graph construction and community analysis

A directed graph is built from all the similarity results within the similarity table using the R package for complex network research igraph [32], the similarity table is imported using the function *graph_from_data_frame* with the option *directed = TRUE*. Thus, in the igraph network architecture, the nodes represent cluster/groups defined by the user, and edges represent the similarity score. Edges are directed and point in the direction of the similarity score computed from cluster/group A to B meaning how similar is cluster A to cluster B, the weight (length) of edges correspond with the magnitude of the similarity score.

CFS will compute the network using igraph with the n top similarities desired by the user, as an example, if only the top one similarity is obtained with CFS (option TopN = 1), then each cluster n from dataset i (a node on the graph) will have only one outward edge (arrow) directed towards one cluster from dataset i + 1 (another node of the graph). If the top two similarities are specified, each node will have two edges pointing outwards per external dataset. Once the graph has been built, we visualize the network using the function *layout_with_gem* from igraph, which places vertices on the plane using the GEM force-directed layout algorithm [33].

CFS can also perform a community analysis using the Leiden algorithm on the constructed graph, by using the package function *findCommunitiesSimmilarity* (which makes use of the igraph function *graph_community_leiden*), this analysis returns a table with the detected communities, and which clusters/groups correspond to each community, helping to aggregate the similar groups of clusters algorithmically, additionally the communities are depicted in different colors in the graph. The Leiden algorithm consists of three phases: [1] local moving of nodes, [2] refinement of the partition, and [3] aggregation of the network based on the refined partition, using the non-refined partition to create an initial partition for the aggregate network.

### Mice motor cortex and spinal cord nuclei isolation and single nuclei RNA sequencing

Wild-type mice (C57BL/6NRj, Janvier Labs) were used in this study. Cortex was dissected from the left-brain hemisphere. Spinal cord was dissected and divided into three pieces. All tissues were flash frozen. For nuclei isolation, 20 μg of upper spinal cord (cervical / thoracic) and motor cortex were used. Samples were lysed in 2 ml of lysis buffer (5 mM CaCl_2_, 3 mM magnesium acetate, 0.32 M sucrose, 0.1 mM EDTA pH8.0, 10 mM Tris-HCl pH, 1 mM DTT, 0.5% Triton X-100) using a dounce homogenizer. Next, 3.7 ml of sucrose solution (1.8 M sucrose, 3 mM magnesium acetate, 1 mM DTT, 10 mM Tris-HCl pH8.0), and the ultracentrifugation tubes were filled up with the lysis buffer. Ultracentrifugation was performed at 24 400 rpm (107 164 rcf) for 2.5 h at 4°C in a SW28 swing rotor (Beckman Coulter). Supernatant was discarded and the pelleted nuclei were resuspended on ice in 200 μl of DEPC-treated water-based 1X PBS. To get rid of any debris or nuclei aggregates, pre-separation filters of 30-μm pore size were used (Miltenyi Biotec). Nuclei were counted and the suspension was adjusted to 1000 nuclei per microliter. For single-nuclei capture, 5000 nuclei per sample were loaded on the Chromium controller (10x Genomics). All downstream steps were performed as described in the manufacturer's protocol in the Chromium Next GEM Single Cell 3′ Kit v3.1. The libraries were sequenced on a NextSeq 550 System (Illumina) using the NextSeq 500/550 High Output Kit v2.5 (150 Cycles).

### Mice motor cortex and spinal cord single-nuclei RNA Seq bioinformatic analysis

Sequencing files from the 6 samples were processed using cellranger software version-6.0.1 and aligned to Genome Mouse Build 38 (mm10). Cells with more than 1.5% of mitochondrial content, library size inferior to 1500 counts, and less than 250 genes detected, were filter out. Downstream analysis was conducted individually using Seurat package on each of the six individual samples (tissue-mouse pairs). Cell type labeling was done using type specific markers from the scientific literature.

Astrocyte population was then subtracted for re-analysis, selecting clusters that showed unambiguous and distinct expression of astrocyte markers. The number of principal components for calculating nearest neighbors was chosen using both JackStraw and elbow methods in a data driven manner for each sample. Re-clustering was done using Louvain algorithm, testing for granularity parameters that yield a coherent and stable number of clusters.

Similarity scores were obtained with CFS statistical approach, using as a grouping variable the cluster id, and using a subsampling number of n = 30. Feature selection of markers by CFS were obtained by specifying topN = 10 (obtain top 10 markers contributing to the similarity for each cluster pair compared). Markers depicted in Fig. 3C were selected as being a similarity contributor in more than one cluster pair contrast.

Differential expression between Astrocyte Groups (AG) was tested using a negative binomial model using the merged expression from all mice and tissues, and including as model covariates the tissue, the sequencing date, and the mouse id.

Cell-cell communication analysis was done using CellChat [34] version 2 [35] with default parameters, this method is based on literature-supported ligand-receptor interactions. Groups interrogated for cell-cell communication were obtained by clustering the neuronal populations using the merged data from all mice and only the motor cortex region, and the communication analysis was done between neuronal clusters and the three astrocyte populations described before.

Trajectory analysis, pseudotime scores, and graph analysis to identify differential expressed genes as a function of pseudotime for AG3 were obtained using Monocle2 [36] by using the raw merged expression of AG3 subpopulations from all mice and tissues and using Monocle2 default parameters. Gene set enrichment analysis was obtained with gprofiler2 R package.

### Benchmark of CFS and different integrative methods

Inspired by the approach used by Ghazanfar et al. [16], we adapted extended and performed a benchmark comparison with other integrative and batch correction algorithms: MultiMap; naive PCA with no batch correction, PCA corrected using MNN (mutual nearest neighbors), and PCA corrected using Harmony; Seurat CCA (canonical correlation analysis); StabMap with no batch correction, StabMap corrected using MNN. We design two different scenarios: a scRNA-Seq pancreatic multispecies datasets [37], composed of mouse and human samples (human *n* = 4, mice *n* = 2), with different sizes of cell populations (human *n* = 7561 cells, mice *n* = 1861 cells), downloaded using R/Bioconductor package scRNAseq. To make this benchmark reproducible we used set.seed(01 022 025). Additionally, and using the pancreas dataset, we computed extra quantitative metrics using the Pearson distance, cosine similarity, and Minkowski distance with *p*= 1.5, again with set.seed(01 022 025) to make it comparable we the previous benchmark. The second scenario uses an assay of scRNA-seq that aims to study mouse gastrulation across entire embryos on E8.5 timepoint [38], downloaded using the MouseGastrulationData R/Bioconductor package [39], using R function set.seed (29 072 024).

For each scenario, a pair of random samples were selected and processed using Seurat workflow (normalized, scaled, computation of top 2000 HVG, and dimensionality reduction), we then selected one sample as the reference dataset, to be used as ground-truth reference with the original annotation, and assign the other sample as the query dataset, keeping it unannotated and subjected to clustering analysis for the group similarity computation. Within each sampling round, we randomly selected a subgroup from the 2000 variable genes (*n* = 25, 50, 100, 250, 500, 1000, 1500), doing this random sampling 4 times for each n gene subgroup (producing four observations of n genes between the same query and reference datasets). For StabMap, we followed the instructions depicted on the manual and benchmarking approach on the original paper, to compute the reference and query joint embedding into a common space, and using similar approaches with naive PCA, UINMF, and MultiMAP.

To compute the accuracy at cell type label inference using CFS, clusters from the query dataset were labeled as the top-similar class group from the reference dataset, assuming that the entire cluster cell composition to be that cell type. Then accuracy was computed as assessing the number of cells from that cluster having the real annotation matching the assigned annotation. To assess the accuracy of the other integrative methods, we followed the approach by Ghazanfar et al., computing the mean accuracy of cell type annotation from the query sample cells using k-nearest neighbors with *k* = 5.

### Data processing

#### Human and mouse single cell RNA-Seq from pancreas

Annotated data from both studies were downloaded using the R Bioconductor package scRNAseq.

Downstream analysis was conducted using Seurat package on each of the datasets. The number of principal components for calculating nearest neighbors was chosen using both JackStraw (*n* = 15). Clustering was done using Louvain algorithm. CFS was run using a subsampling of *n* = 28. Barplots and exploratory analysis of the cell composition of clusters was done using LotOfCells R package [40]. To analyze the correlation between similarity scores and cell type mixture composition of clusters, simulated single cell count data was produced using scDesign2 [41] selecting the Gaussian copula method to compute the model. The same number of cell per cell type were produced to account for population size (*n* = 1200 cells per cell type).

#### RA and OR single-cell RNA-seq data, bulk RNA-seq data, and mass cytometry data

Normalized and filtered data was downloaded from (https://www.immport.org/shared/study/SDY998. Expression was scaled, T-cell subpopulation was extracted from the main dataset, re-analyzed, and re-clustered using Louvain algorithm (“FindNeighbors(reduction = “pca,” dims = 1:25),” “FindClusters(resolution = 1)”). The same approach was followed for Cytof data with clustering resolution of 0.8.

#### PBMCs ATAC-Seq and single cell RNA-Seq

We obtained two public single-cell datasets of human PBMCs. First, an unannotated scATAC-seq of human PBMCs were downloaded from the 10x Genomics website. In addition, a fully processed and high-quality annotated scRNA and ADT dataset was downloaded from https://atlas.fredhutch.org/nygc/multimodal-pbmc (Hao et al., 2021, PMID: 34 062 119) and used without further modifications. For the 10x Genomics dataset, the filtered peak-barcode matrices were loaded into R (v.4.0.0) using the Seurat (v.4.0.1)/Signac (v.1.4.0) functions “Read10X_h5” and “CreateChromatinAssay(min.cells = 10, min.features = 200. Barcodes from the unimodal dataset were filtered for a number of cut sites between 5000 and 50 000 and a transcription start site score > 3.5. Gene activity scores were calculated using the function “GeneActivity” and log-normalized using as scaling factor the median of the gene activity matrix. Subsequently dimensionality reduction was applied based on the peak matrix using top 2000 variable, and cells were clustered using the first 20 PCA dimensions, with a resolution of 0.8. For the final cell type annotation, clusters were annotated based on marker gene expression. CFS was run based on the gene activity matrix (10x Genomics dataset) or the RNA count matrix (ADT dataset) using all common features and fine cell type labels as cell identities, with a subsampling of *n* = 30.

### Statistical significance

Throughout this study, we considered a P-value threshold for statistical significance of 0.005, as it has been shown to foster reproducibility and lower the false-positive rate [42, 43]. Therefore, any claims of significance on this paper should be noted as passing the threshold α = 0.005.

### Compatibility

CFS is compatible with the most popular single-cell pipelines for an easy downstream integration: Seurat [44] and SingleCellExperiment [45].

### Ethics approval

All animal experiments were performed in accordance with institutional guidelines of the University of Heidelberg and were approved by the local authority (Regierungspräsidium Karlsruhe, Germany).

## Results

### CFS Overview

**Figure 1.**
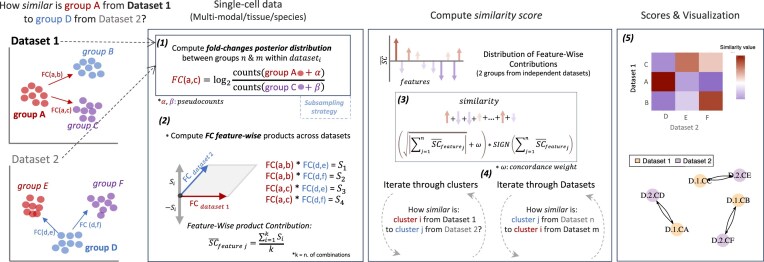
Diagram of CFS for the comparison of two independent datasets. The datasets are depicted by dimensionality reduction (PCA, UMAP, t-SNE, VAE, PATHE, or others) for visualization purposes only.

CFS computes scores for each pair of cell groups based on differences in abundance, these values are centered around 0 (negative scores mean dissimilarity, while positive values are similar phenotypical states based on abundance differences). A summary of the workflow for computing the similarity metric is shown in Fig. 1. The tool expects two or more single-cell datasets, each with their raw feature count matrix, and defined groups of interest at the cell level. The identity of these groups can be a phenotypic characteristic (e.g. cell type, cell cycle, or time series) or an algorithmically obtained label (e.g. clustering techniques). In the first step, differences in abundance from raw expression between the groups in each dataset are estimated using a Bayesian approach. These FCs are normalized and used to compute a similarity metric between groups from independent single-cell assays. Additionally, the contribution of each feature to the similarity score is used to understand its importance as a marker of these specific groups of cells, acting as a feature importance discovery tool. To understand the complex interaction between the groups and their similarities, CFS builds a directed graph using the igraph R package framework [32], in which edges represent the direction (arrow) and intensity (length) of similarities from cluster source to cluster target. An additional community algorithm can be applied to find communities of clusters from the similarity graph, helping to identify the most similar cell communities.

### Comparison with popular integrative methods

**Figure 2.**
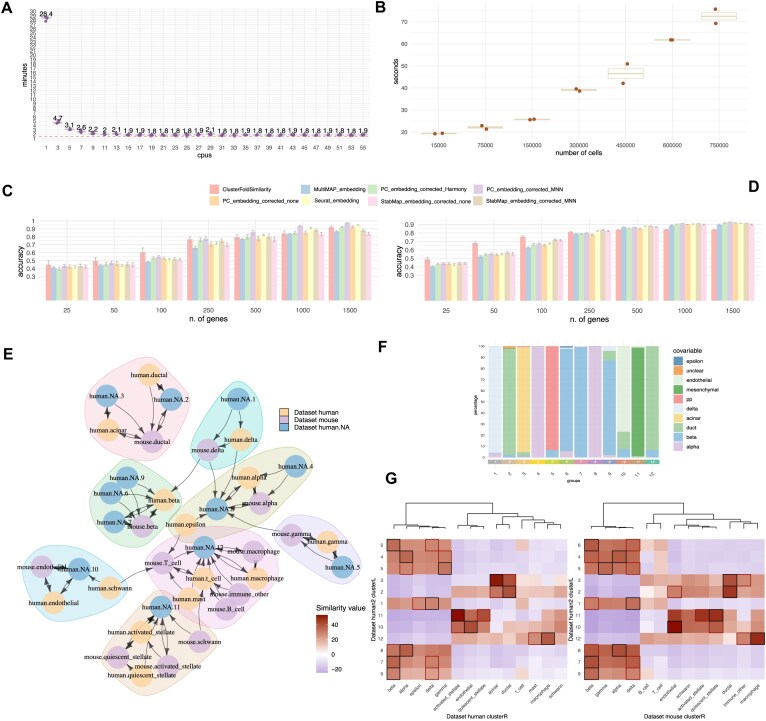
(**A**) Benchmark of computing times of CFS using different numbers of CPUs. The artificial dataset is composed of 750k cells, divided into three datasets with 20 clusters each (*k* = 6819 combinations). (**B**) Benchmark of computing times of CFS using different dataset sizes using a fixed number of 17 CPUs. The artificial datasets are composed from 15k to 750k cells, divided into three datasets with 20 clusters each (*k* = 6819 combinations). (**C**) Barplot displaying mean cell type classification accuracy results of the Benchmark comparison against other integrative methods using two pancreatic scRNA-Seq datasets, each gene subset includes several permutations per gene sampling. Error bars represent the standard error of the mean. (**D**) Barplot showing mean accuracy results of cell type classification benchmark comparison against integrative methods using a mice gastrulation scRNA-Seq datasets (*n* = 4 mouse) on E8. Error bars represent the SEM. (**E**) Directed graph of clusters by top similarity; each node corresponds to a cluster/cell type group from one dataset (human and mice). Length of edges corresponds with the magnitude of the similarity and arrows depict the direction of the similarity test. (**F**) Proportion of cell types per cluster using the authors annotation (Muraro et al.) from human pancreatic single cell dataset used as an unannotated target (human.NA). (**G**) Heatmap showing all computed similarity scores (all possible combinations of groups) between the datasets.

CFS can process a total of 750k cells, divided into three datasets with 20 clusters each (*k* = 6819 combinations) in three minutes using seven cores (Fig. 2A). Additionally, we tested how CFS is able to scale with larger data sizes, for this purpose, we measure the processing time of datasets composed of 15–750k cells, again divided into three datasets with 20 clusters each (*k* = 6819 combinations), and a fixed number of 17 CPUs, we demonstrate that the increase in processing time with size is almost linear (Fig. 2B). To assess the performance of CFS against other popular methods, we first selected two public annotated datasets and computed the accuracy of predicting the cell type by cluster similarities. The first scenario is composed of two scRNA-Seq pancreatic tissue datasets, from mouse and human samples [37]. The second scenario uses an assay of scRNA-seq that aims to study mouse gastrulation across entire embryos on E8.5 time point [38]. These datasets were used as ground-truth reference and unlabeled query iteratively, with cell groups defined based on their known cell-type annotation by the original authors. For the pancreatic scRNA-Seq, apart from the organism and sample batch effect (human samples *n* = 4, mice individuals *n* = 2), these datasets have a large difference in the number of available cells (human dataset *n* = 7561 cells, mice dataset *n* = 1861 cells), which allowed us to benchmark the sample size impact on the performance (Supplementary Fig. S2A), and show that on average CFS is not significantly impacted by the varying size of the datasets. We performed an extensive benchmark comparison with other integrative and batch correction algorithms: MultiMap; naive PCA with no batch correction, PCA corrected using MNN (mutual nearest neighbors), and PCA corrected using Harmony; Seurat CCA (canonical correlation analysis); StabMap with no batch correction, StabMap corrected using MNN. We iterated across all datasets, using each as the reference and the query, and with varying number of available features (*n* = 25, 50, 100, 250, 500, 1000, 1500) following a similar approach as depicted by Ghazanfar et al. [16]. In our case, for each reference-query pair we select from the top 2000 high variable features n random genes more than once (producing several predictions for the same reference-query n features pair), to assess the accuracy for different sets of genes. Results show high accuracy of CFS at cell type mapping task, especially when low number of shared features are available (Fig. 2C and D), case in which CFS has a better performance, highlighting that it can be applied in scenarios where the number common shared features is low, like multi-organism or multi-modal (e.g. single-cell proteomics with extremely small number of surface markers screened).

In general, results suggest that the performance and accuracy of integrative single-cell methods are dataset-dependent, as an example StabMap showed a varying performance, being one of the topmost accurate in the gastrulation dataset (Fig. 2D), but one of the worst performing in the pancreatic multi-species dataset (Fig 2C). This again highlights that a careful consideration of the selected method should be done for each independent single-cell data analysis, and many factors must be considered beforehand, like the number of features available, size of the datasets, degree of tissue/organism/technical batch effects, and others.

Finally, and to add extra quantitative metrics with more closely related similarity metrics, we performed a benchmark comparing CFS to the Pearson distance, cosine similarity, and Minkowski distance with *p*= 1.5 (a generalization in between the Manhattan distance and the Euclidean distance), computing these metrics using the count matrix from the pancreas dataset. We choose again this dataset both because it will let us compare these metrics with the already tested methods, and because its low-complexity with mild batch effect. Results clearly indicate the superiority of our CFS metric (Supplementary Fig. S2B), with the other distance scores having an empirical upper limit on this scenario of around 75% accuracy at matching clusters and cell types.

### CFS effectively compares groups of mice and human cells

We used one mouse and two publicly available human single-cell RNA-Seq datasets from pancreatic tissue, each with annotated cell-type labels, to evaluate the accuracy of the similarity metric. The first human dataset [46] was subjected to unsupervised clustering to interrogate the cell-type composition, retaining the annotated cell types for later comparison. The other two datasets, from mouse and human pancreatic samples [37], were used as ground-truth references, with cell groups defined based on their known cell-type annotation by the original authors.

To assess the pair matches between the clusters in the unannotated human dataset and the cell type groups in the remaining studies, we computed and selected the top similarity scores for each of the 11 unannotated clusters (Supplementary Table S1). This approach allowed us to confidently establish matches between clusters and annotated cell type groups (Fig. 2E–G). The results show that 10 out of 11 clusters exhibited perfect matches to the known annotations of the clusters from the original publication and both reference datasets.

Notably, the only exception (cluster 3), corresponding to acinar cells, displayed the highest similarity with the acinar cell group from the second human dataset and the ductal cell group from the mouse dataset. This could be expected, as acinar cells were absent in the annotation of the utilized mouse dataset. Notably, our similarity metric successfully indicated the closest related cell type by lineage: ductal cells. Furthermore, we observed a consistent association for cluster 11, which represents mesenchymal cells (a cell type that is not present in both reference datasets), with stellate cells, which were missing in the clustered human dataset, but present in both mouse and second human reference datasets. This association has been previously demonstrated in the literature [47].

Our results demonstrated the capability of our algorithmic approach to successfully match comparable phenotypic cell groups, even when working with different organisms. Notably, using more than one dataset as a reference helped to clear and define the broad relationships between cell populations (Fig. 2E); we believe that ClusterFoldSimilarity will benefit for the numerous public cell atlases available. However, it is important to acknowledge the potential limitation of our metric, which operates at the cell group level rather than at the individual cell level, which may lead to some loss of resolution in the annotation of heterogeneous clusters. However, we believe that this issue can be mitigated to some extent by increasing the granularity in the clustering analysis or by using existing meta-cell analysis algorithms that capture small cell groups with the same technical variability and thus maximize data resolution [48–50].

### Correlation between cell mixture proportion and similarity score

To investigate the effect of different cell mixtures within clusters on our similarity metric, we generated an artificial single-cell RNA-Seq dataset using the scDesign2 algorithm [41]. The data were constructed based on a human pancreatic dataset [37] with known cell type labels. We adjusted the artificially recreated count matrix to ensure an identical number of cells per cell type (*n* = 1200), thereby minimizing the cluster size effect and fostering the signal of small-cell populations (Supplementary Fig. S2C).

Using CFS, we computed the similarity scores between clusters from the original human pancreatic dataset, which included clusters with cell type mixtures, and the artificially recreated and cell type balanced dataset. Interestingly, the similarity scores demonstrated the ability to effectively capture cell mixture proportions at some extent (Supplementary Fig. S2E). For example, in the original dataset, Cluster 10 comprised up to nine different cell types, with marginal cell types not typically found in the pancreas. The similarity scores mirrored the cell type proportions in descending order: endothelial cells, macrophages, mast cells, Schwann cells, and T-cells (Supplementary Fig. S2E). Only stellate cells exhibited a higher similarity value than anticipated, although they were still lower than those of the top two most abundant cell types. Similar trends were observed in Cluster 4 of the original dataset, composed of five different cell types. The similarity values again correlated with the percentage of cells from each cell type in the following descending order: activated stellate cells, quiescent stellate cells, endothelial cells, acinar cells, and Schwann cells (Supplementary Fig. S2D–F).

Overall, our results demonstrate how CFS is able to capture -at some extent- the complex mixture of cell populations, although we do not believe that it will be suitable for deconvolutional single-cell task, we think it is an important point to take into consideration when analyzing and interpreting CFS results, and we believe similarity values exhibit great potential for downstream analysis, and can be integrated in combination with additional bioinformatics methods.

### Expanding to multimodal assays: single-cell proteomics, sorted bulk RNA-Seq and single-cell RNA-Seq

To test whether cell populations from additional multimodalities can be linked using our technique, we analyzed 51 samples of synovial tissue biopsies from rheumatoid arthritis (RA) and osteoarthritis (OA) from sorted B cells, T cells, monocytes, and stromal fibroblast populations [51]. The multimodal study included sorted bulk RNA-seq from all patients (*n* = 54 patients), single-cell RNA expression (n = 21), and single-cell protein expression from a 34-marker mass cytometry panel (Cytof, *n* = 26).

We processed the three datasets independently, obtaining 13 clusters for the single cell RNA-Seq assay to be used as targets of the annotation by similarity, and used the cell type annotations from the original authors as ground truth labels for both bulk RNA-Seq and Cytof. Annotations for the computed single cell RNA-Seq clusters were retrieved and compared with the unannotated cluster for later comparison (fibroblast clusters 0, 4, 6; T cell clusters 1, 5, 8, 9, 12; B cells clusters: 2, 7, 11; monocyte clusters: 3, 10). The data processing differs considerably from the original study, as an integration strategy is used by the authors, which might negatively impact the clustering analysis especially in the case of cell subtypes.

The similarity metric was computed using 33 common features across all datasets (proteins in the case of mass spectrometry data and genes coding for those proteins in the case of RNA-Seq assays). For all the cell type classes present in the assays (B cells, T cells, monocytes, and fibroblasts) clusters of cells with the same pre-annotation were unambiguously mapped together using our similarity metric (Supplementary Table S1) for all three data modalities, including the unannotated clusters (Supplementary Fig. S3A and B). Only the small cluster CD4 + HLA-DR + from the Cytof data, composed of T cells, was linked per top similarity to cluster 3 from single cell RNA-Seq, composed of monocytes. Although the same cluster 3 (monocytes) from single cell RNA-Seq assay showed as its top similarity the Cytof cell group CD11c + CD38 − CD64+ (Supplementary Fig. S3A), which is composed solely of monocytes. This shows that using more than two datasets can improve the similarity matching and thus the understanding of complex interactions between groups. Notably, even with an extremely small number of common features available (*n* = 33), our approach demonstrates to be robust and accurate, showing again the flexibility and adaptability of the methodology.

### Similarity matches population across single-cell RNA-seq and ATAC-seq data

We applied our novel methodology to multimodal single-cell data comprising RNA-seq and ATAC-seq from two PBMC datasets. As a reference, we used a high-quality CITE-seq atlas of the circulating human immune system, comprising 160k cells with 26 annotated cell types [44]. Additionally, we processed one non-annotated public 10x ATAC-Seq dataset, obtaining 18 clusters that were manually labeled by cell type using canonical markers, including fine annotations for specialized subtypes, with a total of 15 different phenotype characterizations. We then computed the similarity metric from the ATAC-Seq clusters against the reference ground-truth CITE-seq annotated data. For ATAC-Seq, the gene activity matrix was computed from peak calling using Signac, easing the mapping between features from different modalities and datasets.

Overall, similarity scores were able to link clusters from ATAC-Seq data to reference cell populations in RNA-Seq data with identical phenotypes (Supplementary Table S1), including small populations such as plasmacytoid Dendritic Cells and myeloid Dendritic Cells (mDCs), and differentiate between specialized subgroups of CD8 and CD4 of T-memory VS T-naïve (Supplementary Fig. S3C). Results showed that out of 17 comparable cell populations, 14 obtained top similarity values with the corresponding and matching cell types. The second and third most similar groups were the corresponding types from those that did not map with the most similar group. For instance, non-classical monocytes top similar group in the reference was classical monocytes, followed closely as second most similar by the non-classical monocytes. Interestingly, one cluster of CD8 T-memory cells was found to be most similar to Natural Killer cells, and KLRD1 selected by our tool (Supplementary Fig. S3D), the marker gene contributing the most to the score. KLRD1 is used as an NK marker and is known to be expressed in CD8 cytotoxic cells [52–55]. The top markers selected by feature selection ability of CFS were in general highly specific (Supplementary Fig. S3D).

In the case of some highly specialized manually annotated groups that were not present in the reference dataset, cell types engaging in those specific phenotypes were found to be the most similar (e.g. the top similar group for the ATAC-Seq manually annotated T helper cell clusters was CD4 naïve from the reference single-cell RNA-Seq, Supplementary Fig. S3C).

These results show that our method is a powerful tool not only for matching clusters or finding markers of interest but can also be used in conjunction with high-quality reference datasets.

### Astrocyte analysis on mouse motor cortex and spinal cord

**Figure 3.**
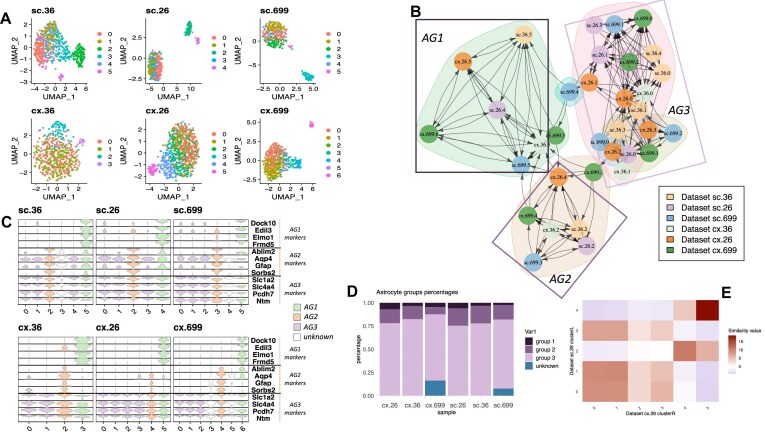
Integration of astrocyte cluster subpopulation using our similarity metric. (**A**) UMAP dimensionality reduction of datasets (N = 6) sc:spinal cord, cx:cortex. (**B**) Directed graph of clusters by similarity, each node corresponds to a cluster from one dataset, edges correspond to the similarity value and the direction of the similarity, shorter edge lengths translate to larger similarity values. (**C**) Violin plots of astrocyte gene markers selected by CFS as contributing the most to the similarity value for the specified AG1, AG2, and AG3 subpopulations. (**D**) Percentage of cells corresponding to each AG per sample. (**E**) Heatmap plot of all the similarity values between spinal cord and cortex from the same mouse (id:26).

To further demonstrate the versatility of our metric, we sequenced and analyzed single-nuclei RNA-Seq from the motor cortex and spinal cord of three wild-type 12 months old mice, for a total of six samples (Fig. 3A), with the focus of identifying and characterizing astrocytes in different parts of the motor-neuronal system. Astrocytes are glial cells in the central nervous system with a wide diversity of roles and physiological functions, including development and maturation of synapses, neurotransmitter homeostasis, glycogen storage, blood brain barrier maintenance, and clearance of protein aggregates [56–59]. They have become of high interest as they contribute to several neurological diseases, such as the motor disease amyotrophic lateral sclerosis (ALS) [60–62]. After processing and filtering for quality control, a total of 39 493 cell-nuclei were selected for downstream analysis (mean of nuclei per mouse = 6582, sd = 1107). Astrocytic cell populations were selected using respective canonical markers (Fig. 3C), for a total of 3056 astrocytes (mean per mouse = 509 astrocytes, sd = 167).

An initial integration of the expression data revealed, as expected, major differences between spinal cord and motor cortex (Supplementary Fig. S4B and E). To overcome the tissue and intra-specimen variability (Supplementary Fig. S4A, B, D, and E) and identify conserved astrocyte populations, we used our similarity score analysis to match clusters between mice. For this, each sample was independently processed (Fig. 3A) and clustered (see “Materials and methods”). The processed samples were then fed into our similarity score algorithmic approach, using all available genes, so that novel markers outside the top variable genes could be detected by CFS feature selection ability.

Exploratory analysis of common markers like Gfap and Aqp4 showed high variability (Fig. 3C), revealing the emerging heterogeneity and diverse functionality of astrocytes [63, 64]. Glial fibrillary acidic protein (Gfap), while showing overall expression in spinal cord, was cluster-specific in motor cortex, showing the limitations of this marker as previously described [65, 66]. In the same direction, Aquaporin 4 (Aqp4), highly expressed in astrocytes and responsible for water exchange across the brain, showed consistent expression in spinal cord astrocyte populations, but not in the motor cortex. The enzyme aldehyde dehydrogenase 1 family member L1 (Aldh1l1), regulating folate metabolism and known to be strongly expressed in astrocytes, showed overall inconsistent expression across tissues and clusters.

### Similarity score identifies conserved astrocyte populations across motor cortex and spinal cord

Using the similarity scores from the clusters of each sample and the graph community analysis using Leiden algorithm, we defined three distinct subpopulations of astrocytes based on differences in transcript abundances (Fig. 3B–E). Isolated clusters with few or no inward arrows on the similarity graph were excluded (cx.699 clusters 3 and 5, sc.699 cluster 4, and sc.36 cluster 3). These subpopulations were observed in both motor cortex and spinal cord. Top features selected by CFS as the ones contributing the most to the observed similarities were highly specific in most of the cases (Fig. 3C) and in they were observed in the significant DEG found on the contrast analysis withing each group (see next section). Astrocyte Group 3 (AG3) was the largest and most heterogeneous, comprising three additional sub-groups showcasing similar characteristics but different expression patterns. In contrast, AG1 was the smallest in terms of number of cells, yet exhibiting high homogeneity, and showing highly specific markers found by our similarity method. AG2 resembled GFAP + physiologically active population, showing upregulated genes that are shared with pathogenic reactive populations [67].

To better understand the main differences between these three AG populations, we performed a differential expression analysis between the groups discovered by CFS pulling all the data together and adding as model covariates for the DE test the mouse id and sequencing date. A justification for this is that the marker selection capabilities of CFS are complementary to differential expression analysis, but a more refined test is needed to uncover the significant differences once the main groups have been identified, since features contributing to the similarity between clusters can be relevant for several cluster pairs and thus not necessarily differentially expressed between main groups.

### A small astrocyte population enriched in neuronal and CNS developmental markers

**Figure 4.**
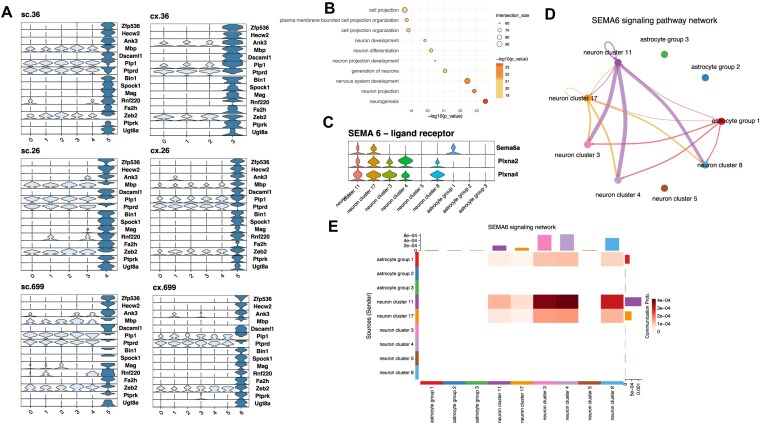
Detailed analysis of AG1. (**A**) Gene markers related with neurogenesis and neuronal development. Dark blue violin plots correspond to cell clusters of AG1 in individual samples (cx: cortex, sc:spinal cord). **(B**) Gene ontology enrichment analysis results of the significantly upregulated markers in AG1. (**C**) SEMA6 signaling pathway ligand-receptor components RNA expression across neuronal clusters and AGs. (**D**) SEMA6 cell-cell communication network analysis between neuronal clusters and AGs. The only AG actively communicating is AG1. (**E**) Heatmap of the SEMA6 signaling pathway and the role in the communication of the different neuronal and AGs.

In total, we found 330 differentially upregulated genes (*P* value < 0.005, see materials and methods statistical significance) in Astrocyte Group 1 (AG1) in contrast with AG2 and AG3 (Table 1), both in spinal cord and motor cortex tissues (differential expression analysis was done using the merged data with the tissue, sequencing date, and the mouse id as covariates). A large quantity of these upregulated genes were linked to neurogenesis and nervous system development functions (Fig. 4A and B). Ontology analysis revealed a set of major biological terms significantly associated with neurogenesis, nervous system development, neuron projection, neuron differentiation, and cell projection organization (Fig. 4B). Relevant actors in this upregulated list include a set of transcription factors (TF) uniquely expressed in AG1. Sox10 involved in embryonic development and cell fate, has been described to play an important role in neural crest and peripheral nervous system development [68], as well as activating additional TFs [69, 70]. Zfp536 with roles in regulation of neuron differentiation and negative regulation of retinoic acid receptor signaling pathway [71, 72]. Zeb2 (Sip1, Zfhx1b), a TF that acts both as a DNA-binding transcriptional repressor and activator, is known to be involved in neuronal development [73] and in the differentiation between cortical and striatal interneurons [74]; Zeb2 shows expression across all groups in our study, being significantly higher in AG1.

**Table 1.** Differentially upregulated genes (*P* value < 0.005) in AG1 in comparison with AG2 and AG3

Gene	p_val	avg_log2FC	pct.1	pct.2	p_val_adj	Description
Tmeff2	1.96E-279	2.190 353	0.866	0.171	6.32E-275	transmembrane protein with EGF-like and two follistatin-like domains 2 [Source:MGI Symbol;Acc:MGI:1 861 735]
Slc24a2	2.18E-271	2.39 047 055	0.901	0.313	7.04E-267	solute carrier family 24 (sodium/potassium/calcium exchanger), member 2 [Source:MGI Symbol;Acc:MGI:1 923 626]
St18	6.00E-247	1.99 495 027	0.831	0.065	1.94E-242	suppression of tumorigenicity 18 [Source:MGI Symbol;Acc:MGI:2 446 700]
Pde4b	1.48E-234	2.43 293 918	0.958	0.518	4.79E-230	phosphodiesterase 4B, cAMP specific [Source:MGI Symbol;Acc:MGI:99 557]
Edil3	1.19E-227	1.90 688 869	0.838	0.19	3.86E-223	EGF-like repeats and discoidin I-like domains 3 [Source:MGI Symbol;Acc:MGI:1 329 025]
Zfp536	4.61E-204	1.59 566 407	0.789	0.046	1.49E-199	zinc finger protein 536 [Source:MGI Symbol;Acc:MGI:1 926 102]
Dock10	2.99E-202	1.82 246 249	0.859	0.107	9.65E-198	dedicator of cytokinesis 10 [Source:MGI Symbol;Acc:MGI:2 146 320]
Frmd5	2.29E-187	1.91 664 375	0.81	0.105	7.40E-183	FERM domain containing 5 [Source:MGI Symbol;Acc:MGI:2 442 557]
Pex5l	2.43E-179	2.1 784 658	0.817	0.187	7.84E-175	peroxisomal biogenesis factor 5-like [Source:MGI Symbol;Acc:MGI:1 916 672]
Prr5l	8.98E-164	2.11 001 049	0.838	0.084	2.90E-159	proline rich 5 like [Source:MGI Symbol;Acc:MGI:1 919 696]
Map7	3.57E-156	1.42 866 784	0.739	0.155	1.15E-151	microtubule-associated protein 7 [Source:MGI Symbol;Acc:MGI:1 328 328]
Hecw2	1.21E-141	1.3 025 446	0.697	0.091	3.89E-137	HECT, C2 and WW domain containing E3 ubiquitin protein ligase 2 [Source:MGI Symbol;Acc:MGI:2 685 817]
Ank3	5.63E-131	1.39 801 683	0.824	0.241	1.82E-126	ankyrin 3, epithelial [Source:MGI Symbol;Acc:MGI:88 026]
Mbp	3.03E-122	1.61 563 575	0.873	0.384	9.80E-118	myelin basic protein [Source:MGI Symbol;Acc:MGI:96 925]
Dscaml1	5.79E-122	1.14 094 576	0.592	0.031	1.87E-117	DS cell adhesion molecule like 1 [Source:MGI Symbol;Acc:MGI:2 150 309]
Ano4	2.70E-120	1.19 501 179	0.662	0.078	8.70E-116	anoctamin 4 [Source:MGI Symbol;Acc:MGI:2 443 344]
Nkain2	1.23E-118	2.459 105	0.923	0.479	3.99E-114	Na+/K + transporting ATPase interacting 2 [Source:MGI Symbol;Acc:MGI:1 923 447]
Plp1	4.27E-111	1.18 847 623	0.93	0.614	1.38E-106	proteolipid protein (myelin) 1 [Source:MGI Symbol;Acc:MGI:97 623]
Elmo1	7.68E-110	1.49 626 476	0.775	0.111	2.48E-105	engulfment and cell motility 1 [Source:MGI Symbol;Acc:MGI:2 153 044]
Plcl1	5.42E-109	1.79 213 873	0.958	0.551	1.75E-104	phospholipase C-like 1 [Source:MGI Symbol;Acc:MGI:3 036 262]
Mog	5.45E-105	0.83 664 247	0.528	0.057	1.76E-100	myelin oligodendrocyte glycoprotein [Source:MGI Symbol;Acc:MGI:97 435]
Spock1	3.35E-104	1.06 025 645	0.62	0.114	1.08E-99	sparc/osteonectin, cwcv and kazal-like domains proteoglycan 1 [Source:MGI Symbol;Acc:MGI:105 371]
C030029H02Rik	6.53E-104	0.87 497 805	0.535	0.013	2.11E-99	RIKEN cDNA C030029H02 gene [Source:MGI Symbol;Acc:MGI:1 924 633]
Bin1	1.85E-100	0.96 607 469	0.676	0.135	5.98E-96	bridging integrator 1 [Source:MGI Symbol;Acc:MGI:108 092]
Ptprd	1.32E-99	1.33 071 484	0.979	0.842	4.26E-95	protein tyrosine phosphatase, receptor type, D [Source:MGI Symbol;Acc:MGI:97 812]
Mag	2.63E-98	0.8 681 837	0.563	0.104	8.50E-94	myelin-associated glycoprotein [Source:MGI Symbol;Acc:MGI:96 912]
Ypel2	4.47E-98	0.90 782 693	0.556	0.075	1.44E-93	yippee like 2 [Source:MGI Symbol;Acc:MGI:1 925 114]
Ppp1r16b	9.04E-98	1.05 503 186	0.599	0.089	2.92E-93	protein phosphatase 1, regulatory subunit 16B [Source:MGI Symbol;Acc:MGI:2 151 841]
D7Ertd443e	7.06E-96	0.95 018 319	0.542	0.033	2.28E-91	DNA segment, Chr 7, ERATO Doi 443, expressed [Source:MGI Symbol;Acc:MGI:1 196 431]
Sec14l5	7.85E-96	0.8 238 531	0.493	0.024	2.53E-91	SEC14-like lipid binding 5 [Source:MGI Symbol;Acc:MGI:3 616 084]
Bcas1	2.14E-95	0.8 599 487	0.528	0.058	6.91E-91	brain enriched myelin associated protein 1 [Source:MGI Symbol;Acc:MGI:1 924 210]
Plekhh1	1.69E-93	0.67 017 468	0.444	0.022	5.46E-89	pleckstrin homology domain containing, family H (with MyTH4 domain) member 1 [Source:MGI Symbol;Acc:MGI:2 144 989]
Rnf220	3.11E-90	1.51 733 162	0.739	0.152	1.00E-85	ring finger protein 220 [Source:MGI Symbol;Acc:MGI:1 913 993]
Fa2h	3.38E-87	0.75 377 598	0.437	0.03	1.09E-82	fatty acid 2-hydroxylase [Source:MGI Symbol;Acc:MGI:2 443 327]
Gjc3	1.13E-85	0.75 679 755	0.472	0.035	3.66E-81	gap junction protein, gamma 3 [Source:MGI Symbol;Acc:MGI:2 153 041]
Zeb2	1.86E-84	1.28 891 095	0.88	0.53	6.02E-80	zinc finger E-box binding homeobox 2 [Source:MGI Symbol;Acc:MGI:1 344 407]
Agap1	3.01E-81	1.00 618 537	0.732	0.207	9.71E-77	ArfGAP with GTPase domain, ankyrin repeat and PH domain 1 [Source:MGI Symbol;Acc:MGI:2 653 690]
Ptprk	2.86E-79	1.01 913 221	0.613	0.093	9.23E-75	protein tyrosine phosphatase, receptor type, K [Source:MGI Symbol;Acc:MGI:103 310]
Sox2ot	4.38E-78	1.13 822 803	0.866	0.475	1.41E-73	SOX2 overlapping transcript (non-protein coding) [Source:MGI Symbol;Acc:MGI:2 444 112]
A230001M10Rik	3.33E-77	0.72 716 261	0.437	0.008	1.08E-72	RIKEN cDNA A230001M10 gene [Source:MGI Symbol;Acc:MGI:2 443 043]
Pakap	9.74E-77	1.0 263 766	0.563	0.051	3.15E-72	paralemmin A kinase anchor protein [Source:MGI Symbol;Acc:MGI:5 141 924]
4930419G24Rik	1.16E-76	0.70 591 971	0.415	0.017	3.74E-72	RIKEN cDNA 4930419G24 gene [Source:MGI Symbol;Acc:MGI:1 921 124]
Adipor2	2.95E-76	1.02 693 216	0.563	0.153	9.52E-72	adiponectin receptor 2 [Source:MGI Symbol;Acc:MGI:93 830]
Arhgap23	4.40E-76	1.02 552 197	0.669	0.234	1.42E-71	Rho GTPase activating protein 23 [Source:MGI Symbol;Acc:MGI:3 697 726]
Phldb1	1.03E-75	0.68 383 294	0.472	0.065	3.34E-71	pleckstrin homology like domain, family B, member 1 [Source:MGI Symbol;Acc:MGI:2 143 230]
Ugt8a	2.30E-75	0.73 747 839	0.486	0.063	7.42E-71	UDP galactosyltransferase 8A [Source:MGI Symbol;Acc:MGI:109 522]
Shtn1	1.26E-72	0.71 115 032	0.458	0.042	4.07E-68	shootin 1 [Source:MGI Symbol;Acc:MGI:1 918 903]
Pak7	2.67E-70	0.8 747 079	0.486	0.077	8.63E-66	NA
March1	6.51E-69	1.176 595	0.634	0.091	2.10E-64	NA
Mobp	2.10E-68	1.06 170 786	0.648	0.139	6.77E-64	myelin-associated oligodendrocytic basic protein [Source:MGI Symbol;Acc:MGI:108 511]
Csmd3	4.28E-67	1.0 810 812	0.817	0.404	1.38E-62	CUB and Sushi multiple domains 3 [Source:MGI Symbol;Acc:MGI:2 386 403]
Slain1	2.35E-66	0.61 273 458	0.451	0.061	7.58E-62	SLAIN motif family, member 1 [Source:MGI Symbol;Acc:MGI:2 145 578]
A330015K06Rik	1.42E-62	0.64 696 362	0.359	0.023	4.60E-58	RIKEN cDNA A330015K06 gene [Source:MGI Symbol;Acc:MGI:2 443 553]
Enpp2	4.34E-62	1.01 557 397	0.69	0.247	1.40E-57	ectonucleotide pyrophosphatase/phosphodiesterase 2 [Source:MGI Symbol;Acc:MGI:1 321 390]
A330049N07Rik	1.28E-61	0.50 447 518	0.317	0.012	4.14E-57	RIKEN cDNA A330049N07 gene [Source:MGI Symbol;Acc:MGI:3 041 175]
Slco3a1	2.53E-61	0.71 425 882	0.43	0.065	8.17E-57	solute carrier organic anion transporter family, member 3a1 [Source:MGI Symbol;Acc:MGI:1 351 867]
Mapt	3.51E-61	1.03 710 995	0.824	0.44	1.13E-56	microtubule-associated protein tau [Source:MGI Symbol;Acc:MGI:97 180]
Neat1	9.26E-61	0.90 096 116	0.873	0.53	2.99E-56	nuclear paraspeckle assembly transcript 1 (non-protein coding) [Source:MGI Symbol;Acc:MGI:1 914 211]
Sox10	1.48E-60	0.48 456 892	0.317	0.012	4.78E-56	SRY (sex determining region Y)-box 10 [Source:MGI Symbol;Acc:MGI:98 358]
Sh3d19	2.37E-60	0.73 672 366	0.458	0.052	7.64E-56	SH3 domain protein D19 [Source:MGI Symbol;Acc:MGI:1 350 923]
Aspa	2.86E-60	0.83 141 426	0.563	0.125	9.23E-56	aspartoacylase [Source:MGI Symbol;Acc:MGI:87 914]
St6galnac3	9.88E-59	1.36 442 656	0.831	0.169	3.19E-54	ST6 (alpha-N-acetyl-neuraminyl-2,3-beta-galactosyl-1,3)-N-acetylgalactosaminide alpha-2,6-sialyltransferase 3 [Source:MGI Symbol;Acc:MGI:1 341 828]
Dnm3	2.80E-58	1.08 067 896	0.796	0.312	9.05E-54	dynamin 3 [Source:MGI Symbol;Acc:MGI:1 341 299]
Mast4	6.02E-58	1.17 111 178	0.923	0.662	1.94E-53	microtubule associated serine/threonine kinase family member 4 [Source:MGI Symbol;Acc:MGI:1 918 885]
Spock3	1.06E-55	0.82 781 574	0.5	0.146	3.42E-51	sparc/osteonectin, cwcv and kazal-like domains proteoglycan 3 [Source:MGI Symbol;Acc:MGI:1 920 152]
Tspan2	1.59E-55	0.53 547 825	0.387	0.042	5.14E-51	tetraspanin 2 [Source:MGI Symbol;Acc:MGI:1 917 997]
Pstpip2	7.48E-55	0.6 397 393	0.352	0.027	2.41E-50	proline-serine-threonine phosphatase-interacting protein 2 [Source:MGI Symbol;Acc:MGI:1 335 088]
Myo1d	2.03E-53	0.5 941 816	0.338	0.019	6.54E-49	myosin ID [Source:MGI Symbol;Acc:MGI:107 728]
Trf	2.50E-52	0.74 161 891	0.57	0.223	8.09E-48	transferrin [Source:MGI Symbol;Acc:MGI:98 821]
Gm37459	6.58E-51	0.47 492 859	0.289	0.013	2.12E-46	predicted gene, 37 459 [Source:MGI Symbol;Acc:MGI:5 610 687]
Gatm	3.61E-49	0.6 767 977	0.549	0.162	1.17E-44	glycine amidinotransferase (L-arginine:glycine amidinotransferase) [Source:MGI Symbol;Acc:MGI:1 914 342]
Tbc1d5	2.37E-47	0.96 314 208	0.782	0.393	7.67E-43	TBC1 domain family, member 5 [Source:MGI Symbol;Acc:MGI:1 919 488]
Arhgef10	4.17E-47	0.61 505 565	0.472	0.096	1.35E-42	Rho guanine nucleotide exchange factor (GEF) 10 [Source:MGI Symbol;Acc:MGI:2 444 453]
Magi2	8.29E-47	0.76 494 346	0.993	0.958	2.68E-42	membrane associated guanylate kinase, WW and PDZ domain containing 2 [Source:MGI Symbol;Acc:MGI:1 354 953]
Frmd4b	3.84E-46	0.83 612 737	0.556	0.114	1.24E-41	FERM domain containing 4B [Source:MGI Symbol;Acc:MGI:2 141 794]
Slc25a13	1.13E-45	0.47 421 771	0.282	0.023	3.66E-41	solute carrier family 25 (mitochondrial carrier, adenine nucleotide translocator), member 13 [Source:MGI Symbol;Acc:MGI:1 354 721]
Mast3	1.21E-45	0.54 141 479	0.394	0.061	3.91E-41	microtubule associated serine/threonine kinase 3 [Source:MGI Symbol;Acc:MGI:2 683 541]
9330111N05Rik	2.85E-45	0.86 924 309	0.239	0.02	9.20E-41	RIKEN cDNA 9330111N05 gene [Source:MGI Symbol;Acc:MGI:2 443 112]
Plxdc2	2.90E-45	0.99 119 411	0.697	0.289	9.37E-41	plexin domain containing 2 [Source:MGI Symbol;Acc:MGI:1 914 698]
Hapln2	1.55E-42	0.42 343 615	0.303	0.044	5.01E-38	hyaluronan and proteoglycan link protein 2 [Source:MGI Symbol;Acc:MGI:2 137 300]
Pls1	1.69E-42	0.48 138 908	0.331	0.036	5.45E-38	plastin 1 (I-isoform) [Source:MGI Symbol;Acc:MGI:104 809]
Zdhhc20	1.12E-41	0.71 868 914	0.648	0.271	3.63E-37	zinc finger, DHHC domain containing 20 [Source:MGI Symbol;Acc:MGI:1 923 215]
Kif13b	1.84E-41	0.65 625 565	0.592	0.223	5.93E-37	kinesin family member 13B [Source:MGI Symbol;Acc:MGI:1 098 265]
Nkain1	7.94E-41	0.39 682 336	0.275	0.02	2.56E-36	Na+/K + transporting ATPase interacting 1 [Source:MGI Symbol;Acc:MGI:1 914 399]
Erbin	1.15E-40	0.86 650 298	0.704	0.318	3.73E-36	Erbb2 interacting protein [Source:MGI Symbol;Acc:MGI:1 890 169]
Fnbp1	1.39E-39	0.87 125 728	0.873	0.712	4.50E-35	formin binding protein 1 [Source:MGI Symbol;Acc:MGI:109 606]
Ttll7	2.04E-39	0.60 669 722	0.479	0.149	6.58E-35	tubulin tyrosine ligase-like family, member 7 [Source:MGI Symbol;Acc:MGI:1 918 142]
Srcin1	2.73E-39	0.51 518 035	0.423	0.093	8.83E-35	SRC kinase signaling inhibitor 1 [Source:MGI Symbol;Acc:MGI:1 933 179]
Zdhhc14	4.12E-39	0.68 121 479	0.549	0.153	1.33E-34	zinc finger, DHHC domain containing 14 [Source:MGI Symbol;Acc:MGI:2 653 229]
Arsg	8.39E-39	0.38 634 539	0.268	0.022	2.71E-34	arylsulfatase G [Source:MGI Symbol;Acc:MGI:1 921 258]
Gm42413	1.02E-38	0.3 871 635	0.268	0.028	3.31E-34	predicted gene, 42 413 [Source:MGI Symbol;Acc:MGI:5 648 986]
Tubb4a	1.15E-38	0.54 604 165	0.458	0.133	3.72E-34	tubulin, beta 4A class IVA [Source:MGI Symbol;Acc:MGI:107 848]
Slc12a2	1.22E-38	0.58 801 645	0.521	0.135	3.94E-34	solute carrier family 12, member 2 [Source:MGI Symbol;Acc:MGI:101 924]
Gm16168	2.12E-38	0.87 079 498	0.599	0.205	6.86E-34	predicted gene 16 168 [Source:MGI Symbol;Acc:MGI:3 802 010]
Pcdh9	2.68E-38	0.97 488 422	1	0.984	8.66E-34	protocadherin 9 [Source:MGI Symbol;Acc:MGI:1 306 801]
Pik3c2b	4.28E-38	0.37 728 202	0.246	0.024	1.38E-33	phosphatidylinositol-4-phosphate 3-kinase catalytic subunit type 2 beta [Source:MGI Symbol;Acc:MGI:2 685 045]
4930511M06Rik	1.11E-37	0.3 310 713	0.254	0.018	3.60E-33	RIKEN cDNA 4930511M06 gene [Source:MGI Symbol;Acc:MGI:1 922 334]
Cdc42bpa	2.23E-37	0.78 839 334	0.81	0.573	7.19E-33	CDC42 binding protein kinase alpha [Source:MGI Symbol;Acc:MGI:2 441 841]
Sept4	2.64E-37	0.54 089 898	0.465	0.137	8.53E-33	NA
Ninj2	3.79E-37	0.51 902 061	0.303	0.023	1.22E-32	ninjurin 2 [Source:MGI Symbol;Acc:MGI:1 352 751]
Kcnk13	4.12E-37	0.43 764 788	0.282	0.027	1.33E-32	potassium channel, subfamily K, member 13 [Source:MGI Symbol;Acc:MGI:2 384 976]
Unc5c	6.43E-37	1.11 147 044	0.739	0.212	2.08E-32	unc-5 netrin receptor C [Source:MGI Symbol;Acc:MGI:1 095 412]
Gm19500	2.08E-36	0.30 602 491	0.211	0.008	6.72E-32	predicted gene, 19 500 [Source:MGI Symbol;Acc:MGI:5 011 685]
Il1rap	2.56E-36	0.57 608 255	0.415	0.076	8.25E-32	interleukin 1 receptor accessory protein [Source:MGI Symbol;Acc:MGI:104 975]
Vmp1	5.27E-36	0.66 465 054	0.577	0.251	1.70E-31	vacuole membrane protein 1 [Source:MGI Symbol;Acc:MGI:1 923 159]
Anln	1.38E-35	0.48 487 975	0.303	0.057	4.46E-31	anillin, actin binding protein [Source:MGI Symbol;Acc:MGI:1 920 174]
Tmeff1	4.17E-35	0.51 360 528	0.345	0.075	1.35E-30	transmembrane protein with EGF-like and two follistatin-like domains 1 [Source:MGI Symbol;Acc:MGI:1 926 810]
Pak1	4.91E-35	0.62 796 502	0.627	0.215	1.58E-30	p21 (RAC1) activated kinase 1 [Source:MGI Symbol;Acc:MGI:1 339 975]
Sept7	1.27E-34	0.73 987 847	0.775	0.471	4.09E-30	NA
Creb5	1.29E-34	0.56 432 815	0.387	0.041	4.18E-30	cAMP responsive element binding protein 5 [Source:MGI Symbol;Acc:MGI:2 443 973]
Eml1	1.29E-34	0.56 745 044	0.352	0.083	4.18E-30	echinoderm microtubule associated protein like 1 [Source:MGI Symbol;Acc:MGI:1 915 769]
Slc44a1	3.97E-34	0.58 788 549	0.486	0.126	1.28E-29	solute carrier family 44, member 1 [Source:MGI Symbol;Acc:MGI:2 140 592]
Exoc6b	5.55E-34	0.73 711 601	0.739	0.441	1.79E-29	exocyst complex component 6B [Source:MGI Symbol;Acc:MGI:1 923 164]
Pde8a	1.37E-33	0.68 761 145	0.458	0.094	4.42E-29	phosphodiesterase 8A [Source:MGI Symbol;Acc:MGI:1 277 116]
Phlpp1	1.83E-33	0.81 013 889	0.972	0.776	5.92E-29	PH domain and leucine rich repeat protein phosphatase 1 [Source:MGI Symbol;Acc:MGI:2 138 327]
Synj2	3.19E-33	0.37 727 953	0.303	0.049	1.03E-28	synaptojanin 2 [Source:MGI Symbol;Acc:MGI:1 201 671]
Gm42756	3.27E-33	0.33 667 023	0.204	0.004	1.05E-28	predicted gene 42 756 [Source:MGI Symbol;Acc:MGI:5 662 893]
Rffl	4.32E-33	0.53 223 076	0.387	0.065	1.39E-28	ring finger and FYVE like domain containing protein [Source:MGI Symbol;Acc:MGI:1 914 588]
Cntn2	1.03E-32	0.40 649 633	0.282	0.044	3.31E-28	contactin 2 [Source:MGI Symbol;Acc:MGI:104 518]
Litaf	1.69E-32	0.40 625 268	0.289	0.049	5.44E-28	LPS-induced TN factor [Source:MGI Symbol;Acc:MGI:1 929 512]
Anks1b	3.33E-32	0.75 156 814	0.521	0.256	1.08E-27	ankyrin repeat and sterile alpha motif domain containing 1B [Source:MGI Symbol;Acc:MGI:1 924 781]
Otud7b	7.11E-32	0.65 083 144	0.563	0.222	2.29E-27	OTU domain containing 7B [Source:MGI Symbol;Acc:MGI:2 654 703]
Klhl2	8.54E-32	0.65 820 899	0.542	0.222	2.76E-27	kelch-like 2, Mayven [Source:MGI Symbol;Acc:MGI:1 924 363]
Grb14	1.01E-31	0.4 093 834	0.261	0.032	3.28E-27	growth factor receptor bound protein 14 [Source:MGI Symbol;Acc:MGI:1 355 324]
Cdc37l1	1.36E-31	0.66 387 689	0.683	0.369	4.39E-27	cell division cycle 37-like 1 [Source:MGI Symbol;Acc:MGI:1 914 322]
Sorcs1	2.12E-31	0.81 115 619	0.5	0.12	6.85E-27	sortilin-related VPS10 domain containing receptor 1 [Source:MGI Symbol;Acc:MGI:1 929 666]
Kctd3	6.37E-31	0.43 513 922	0.317	0.079	2.06E-26	potassium channel tetramerisation domain containing 3 [Source:MGI Symbol;Acc:MGI:2 444 629]
Tmem63a	1.55E-30	0.30 592 671	0.218	0.024	4.99E-26	transmembrane protein 63a [Source:MGI Symbol;Acc:MGI:2 384 789]
Agpat4	1.74E-30	0.4 155 855	0.303	0.052	5.61E-26	1-acylglycerol-3-phosphate O-acyltransferase 4 (lysophosphatidic acid acyltransferase, delta) [Source:MGI Symbol;Acc:MGI:1 915 512]
Ssh2	1.52E-29	0.68 483 343	0.556	0.198	4.92E-25	slingshot protein phosphatase 2 [Source:MGI Symbol;Acc:MGI:2 679 255]
Ptgds	2.16E-29	0.78 930 202	0.577	0.396	6.98E-25	prostaglandin D2 synthase (brain) [Source:MGI Symbol;Acc:MGI:99 261]
Opalin	2.55E-29	0.29 885 537	0.211	0.024	8.24E-25	oligodendrocytic myelin paranodal and inner loop protein [Source:MGI Symbol;Acc:MGI:2 657 025]
Gm48508	3.89E-29	0.2 522 365	0.162	0.005	1.26E-24	predicted gene, 48 508 [Source:MGI Symbol;Acc:MGI:6 098 039]
Cpm	7.69E-29	0.26 961 435	0.176	0.011	2.48E-24	carboxypeptidase M [Source:MGI Symbol;Acc:MGI:1 917 824]
Prickle2	1.51E-28	0.81 121 371	0.732	0.389	4.87E-24	prickle planar cell polarity protein 2 [Source:MGI Symbol;Acc:MGI:1 925 144]
Abca8a	2.27E-28	0.30 631 736	0.211	0.012	7.32E-24	ATP-binding cassette, sub-family A (ABC1), member 8a [Source:MGI Symbol;Acc:MGI:2 386 846]
Kndc1	7.59E-28	0.38 605 826	0.225	0.033	2.45E-23	kinase non-catalytic C-lobe domain (KIND) containing 1 [Source:MGI Symbol;Acc:MGI:1 923 734]
Lpgat1	1.47E-27	0.52 009 461	0.43	0.17	4.76E-23	lysophosphatidylglycerol acyltransferase 1 [Source:MGI Symbol;Acc:MGI:2 446 186]
Efnb3	1.57E-27	0.33 242 631	0.246	0.05	5.06E-23	ephrin B3 [Source:MGI Symbol;Acc:MGI:109 196]
Slc8a1	2.10E-26	1.08 822 736	0.746	0.266	6.77E-22	solute carrier family 8 (sodium/calcium exchanger), member 1 [Source:MGI Symbol;Acc:MGI:107 956]
Ubash3b	2.74E-26	0.59 280 785	0.415	0.115	8.85E-22	ubiquitin associated and SH3 domain containing, B [Source:MGI Symbol;Acc:MGI:1 920 078]
Dixdc1	7.79E-26	0.37 251 042	0.275	0.058	2.51E-21	DIX domain containing 1 [Source:MGI Symbol;Acc:MGI:2 679 721]
Rtn4	9.96E-26	0.64 795 108	0.761	0.527	3.21E-21	reticulon 4 [Source:MGI Symbol;Acc:MGI:1 915 835]
Tmem132d	1.74E-25	0.35 903 804	0.232	0.03	5.61E-21	transmembrane protein 132D [Source:MGI Symbol;Acc:MGI:3 044 963]
Lrp1b	2.41E-25	0.74 690 316	0.93	0.817	7.78E-21	low density lipoprotein-related protein 1B [Source:MGI Symbol;Acc:MGI:2 151 136]
Tmem178b	3.75E-25	0.74 144 454	0.704	0.404	1.21E-20	transmembrane protein 178B [Source:MGI Symbol;Acc:MGI:3 647 581]
Car2	4.82E-25	0.56 411 707	0.542	0.278	1.56E-20	carbonic anhydrase 2 [Source:MGI Symbol;Acc:MGI:88 269]
Dock9	6.04E-25	0.60 210 995	0.606	0.322	1.95E-20	dedicator of cytokinesis 9 [Source:MGI Symbol;Acc:MGI:106 321]
Gab1	8.06E-25	0.60 942 824	0.528	0.241	2.60E-20	growth factor receptor bound protein 2-associated protein 1 [Source:MGI Symbol;Acc:MGI:108 088]
Trim2	1.01E-24	0.67 060 973	0.648	0.336	3.27E-20	tripartite motif-containing 2 [Source:MGI Symbol;Acc:MGI:1 933 163]
Gm28376	1.45E-24	0.3 902 197	0.225	0.017	4.69E-20	predicted gene 28 376 [Source:MGI Symbol;Acc:MGI:5 579 082]
Atp8a1	1.75E-24	0.58 217 791	0.725	0.454	5.64E-20	ATPase, aminophospholipid transporter (APLT), class I, type 8A, member 1 [Source:MGI Symbol;Acc:MGI:1 330 848]
Kif21a	2.22E-24	0.64 239 668	0.754	0.427	7.16E-20	kinesin family member 21A [Source:MGI Symbol;Acc:MGI:109 188]
1700047M11Rik	2.26E-24	0.33 808 765	0.232	0.044	7.29E-20	RIKEN cDNA 1700047M11 gene [Source:MGI Symbol;Acc:MGI:1 914 580]
Rasgrp3	2.48E-24	0.32 476 814	0.211	0.02	8.02E-20	RAS, guanyl releasing protein 3 [Source:MGI Symbol;Acc:MGI:3 028 579]
Gm4258	4.16E-24	0.63 782 375	0.563	0.252	1.34E-19	predicted gene 4258 [Source:MGI Symbol;Acc:MGI:3 782 435]
Dip2b	4.33E-24	0.58 787 875	0.606	0.365	1.40E-19	disco interacting protein 2 homolog B [Source:MGI Symbol;Acc:MGI:2 145 977]
Elovl7	1.48E-23	0.30 062 533	0.225	0.026	4.79E-19	ELOVL family member 7, elongation of long chain fatty acids (yeast) [Source:MGI Symbol;Acc:MGI:1 921 809]
Tenm2	1.85E-23	0.63 263 064	0.479	0.244	5.96E-19	teneurin transmembrane protein 2 [Source:MGI Symbol;Acc:MGI:1 345 184]
Rcbtb1	2.41E-23	0.42 611 248	0.331	0.12	7.78E-19	regulator of chromosome condensation (RCC1) and BTB (POZ) domain containing protein 1 [Source:MGI Symbol;Acc:MGI:1 918 580]
Pacs2	1.61E-22	0.40 374 969	0.359	0.121	5.20E-18	phosphofurin acidic cluster sorting protein 2 [Source:MGI Symbol;Acc:MGI:1 924 399]
Inpp5f	2.18E-22	0.43 895 651	0.373	0.152	7.05E-18	inositol polyphosphate-5-phosphatase F [Source:MGI Symbol;Acc:MGI:2 141 867]
Kcnab1	2.87E-22	0.52 369 383	0.444	0.133	9.27E-18	potassium voltage-gated channel, shaker-related subfamily, beta member 1 [Source:MGI Symbol;Acc:MGI:109 155]
Snx30	3.23E-22	0.36 229 522	0.296	0.057	1.04E-17	sorting nexin family member 30 [Source:MGI Symbol;Acc:MGI:2 443 882]
Nbas	3.59E-22	0.46 269 951	0.5	0.223	1.16E-17	neuroblastoma amplified sequence [Source:MGI Symbol;Acc:MGI:1 918 419]
Fign	8.79E-22	0.42 219 088	0.268	0.028	2.84E-17	fidgetin [Source:MGI Symbol;Acc:MGI:1 890 647]
Dgki	9.62E-22	0.80 427 961	0.634	0.277	3.11E-17	diacylglycerol kinase, iota [Source:MGI Symbol;Acc:MGI:2 443 430]
Jph1	9.64E-22	0.25 605 177	0.204	0.025	3.11E-17	junctophilin 1 [Source:MGI Symbol;Acc:MGI:1 891 495]
Prkcq	1.26E-21	0.2 556 603	0.204	0.027	4.06E-17	protein kinase C, theta [Source:MGI Symbol;Acc:MGI:97 601]
Smad7	1.48E-21	0.3 719 771	0.324	0.096	4.78E-17	SMAD family member 7 [Source:MGI Symbol;Acc:MGI:1 100 518]
Kif13a	1.69E-21	0.53 230 798	0.549	0.268	5.45E-17	kinesin family member 13A [Source:MGI Symbol;Acc:MGI:1 098 264]
Rftn1	1.78E-21	0.26 476 314	0.162	0.019	5.75E-17	raftlin lipid raft linker 1 [Source:MGI Symbol;Acc:MGI:1 923 688]
4930420G21Rik	1.96E-21	0.30 770 549	0.176	0.001	6.32E-17	RIKEN cDNA 4930420G21 gene [Source:MGI Symbol;Acc:MGI:1 926 174]
Lpar1	2.02E-21	0.25 219 687	0.197	0.028	6.51E-17	lysophosphatidic acid receptor 1 [Source:MGI Symbol;Acc:MGI:108 429]
Clasp2	6.71E-21	0.62 845 545	0.838	0.669	2.16E-16	CLIP associating protein 2 [Source:MGI Symbol;Acc:MGI:1 923 749]
Stmn4	1.51E-20	0.39 314 171	0.303	0.097	4.88E-16	stathmin-like 4 [Source:MGI Symbol;Acc:MGI:1 931 224]
Il33	2.18E-20	0.45 476 103	0.338	0.112	7.04E-16	interleukin 33 [Source:MGI Symbol;Acc:MGI:1 924 375]
Opcml	3.18E-20	0.63 576 015	0.556	0.323	1.03E-15	opioid binding protein/cell adhesion molecule-like [Source:MGI Symbol;Acc:MGI:97 397]
Mbnl2	3.25E-20	0.59 288 224	0.838	0.596	1.05E-15	muscleblind like splicing factor 2 [Source:MGI Symbol;Acc:MGI:2 145 597]
Ttll5	5.13E-20	0.44 199 473	0.472	0.183	1.66E-15	tubulin tyrosine ligase-like family, member 5 [Source:MGI Symbol;Acc:MGI:2 443 657]
Itpk1	6.27E-20	0.40 133 366	0.352	0.117	2.02E-15	inositol 1,3,4-triphosphate 5/6 kinase [Source:MGI Symbol;Acc:MGI:2 446 159]
Jakmip3	7.34E-20	0.25 099 991	0.162	0.017	2.37E-15	janus kinase and microtubule interacting protein 3 [Source:MGI Symbol;Acc:MGI:1 921 254]
Ppp1cc	9.20E-20	0.38 432 967	0.289	0.098	2.97E-15	protein phosphatase 1 catalytic subunit gamma [Source:MGI Symbol;Acc:MGI:104 872]
Sez6l2	1.10E-19	0.36 167 954	0.338	0.116	3.55E-15	seizure related 6 homolog like 2 [Source:MGI Symbol;Acc:MGI:2 385 295]
Adamtsl1	2.14E-19	0.30 111 108	0.352	0.075	6.91E-15	ADAMTS-like 1 [Source:MGI Symbol;Acc:MGI:1 924 989]
Aatk	3.55E-19	0.50 286 064	0.599	0.274	1.15E-14	apoptosis-associated tyrosine kinase [Source:MGI Symbol;Acc:MGI:1 197 518]
Nfe2l3	5.30E-19	0.25 950 237	0.197	0.027	1.71E-14	nuclear factor, erythroid derived 2, like 3 [Source:MGI Symbol;Acc:MGI:1 339 958]
Dip2a	7.75E-19	0.39 330 794	0.338	0.128	2.50E-14	disco interacting protein 2 homolog A [Source:MGI Symbol;Acc:MGI:2 385 920]
App	8.63E-19	0.57 992 089	0.761	0.539	2.79E-14	amyloid beta (A4) precursor protein [Source:MGI Symbol;Acc:MGI:88 059]
Tmod2	9.60E-19	0.50 543 344	0.585	0.354	3.10E-14	tropomodulin 2 [Source:MGI Symbol;Acc:MGI:1 355 335]
Nbea	1.14E-18	0.55 141 548	0.718	0.527	3.68E-14	neurobeachin [Source:MGI Symbol;Acc:MGI:1 347 075]
Grm3	1.15E-18	0.49 127 603	0.817	0.533	3.70E-14	glutamate receptor, metabotropic 3 [Source:MGI Symbol;Acc:MGI:1 351 340]
Tcf12	1.77E-18	0.49 589 446	0.761	0.561	5.70E-14	transcription factor 12 [Source:MGI Symbol;Acc:MGI:101 877]
Nfasc	1.91E-18	0.35 074 282	0.62	0.4	6.16E-14	neurofascin [Source:MGI Symbol;Acc:MGI:104 753]
Tmem117	3.88E-18	0.40 506 231	0.296	0.089	1.25E-13	transmembrane protein 117 [Source:MGI Symbol;Acc:MGI:2 444 580]
Arrdc3	4.42E-18	0.29 785 618	0.282	0.077	1.43E-13	arrestin domain containing 3 [Source:MGI Symbol;Acc:MGI:2 145 242]
Qk	4.48E-18	0.53 794 064	0.972	0.943	1.45E-13	NA
Arhgap39	4.73E-18	0.47 953 333	0.444	0.146	1.53E-13	Rho GTPase activating protein 39 [Source:MGI Symbol;Acc:MGI:107 858]
Lrrc8b	8.33E-18	0.32 156 334	0.275	0.065	2.69E-13	leucine rich repeat containing 8 family, member B [Source:MGI Symbol;Acc:MGI:2 141 353]
Pacrg	1.41E-17	0.71 534 172	0.606	0.328	4.55E-13	PARK2 co-regulated [Source:MGI Symbol;Acc:MGI:1 916 560]
Cipc	1.41E-17	0.40 268 333	0.408	0.215	4.56E-13	CLOCK interacting protein, circadian [Source:MGI Symbol;Acc:MGI:1 919 185]
Cdk19	2.11E-17	0.59 985 453	0.746	0.469	6.80E-13	cyclin-dependent kinase 19 [Source:MGI Symbol;Acc:MGI:1 925 584]
Apba1	3.45E-17	0.47 134 471	0.401	0.147	1.12E-12	amyloid beta (A4) precursor protein binding, family A, member 1 [Source:MGI Symbol;Acc:MGI:1 860 297]
Kcna1	3.87E-17	0.34 416 244	0.317	0.094	1.25E-12	potassium voltage-gated channel, shaker-related subfamily, member 1 [Source:MGI Symbol;Acc:MGI:96 654]
Prkcz	4.84E-17	0.32 116 027	0.289	0.093	1.56E-12	protein kinase C, zeta [Source:MGI Symbol;Acc:MGI:97 602]
9330182L06Rik	4.86E-17	0.29 562 838	0.232	0.058	1.57E-12	NA
Ube2d1	8.60E-17	0.29 694 192	0.254	0.079	2.78E-12	ubiquitin-conjugating enzyme E2D 1 [Source:MGI Symbol;Acc:MGI:2 384 911]
Dpy19l1	1.64E-16	0.33 789 763	0.289	0.1	5.29E-12	dpy-19-like 1 (C. elegans) [Source:MGI Symbol;Acc:MGI:1 915 685]
1500004A13Rik	3.15E-16	0.37 441 614	0.43	0.193	1.02E-11	RIKEN cDNA 1500004A13 gene [Source:MGI Symbol;Acc:MGI:2 442 808]
Kazn	3.33E-16	0.25 772 965	0.254	0.071	1.08E-11	kazrin, periplakin interacting protein [Source:MGI Symbol;Acc:MGI:1 918 779]
Ccp110	4.68E-16	0.26 463 582	0.218	0.07	1.51E-11	centriolar coiled coil protein 110 [Source:MGI Symbol;Acc:MGI:2 141 942]
Robo1	4.70E-16	0.59 124 202	0.556	0.388	1.52E-11	roundabout guidance receptor 1 [Source:MGI Symbol;Acc:MGI:1 274 781]
Sorcs3	7.52E-16	0.34 798 285	0.275	0.069	2.43E-11	sortilin-related VPS10 domain containing receptor 3 [Source:MGI Symbol;Acc:MGI:1 913 923]
Dlg2	7.57E-16	0.66 353 429	0.873	0.608	2.44E-11	discs large MAGUK scaffold protein 2 [Source:MGI Symbol;Acc:MGI:1 344 351]
Phactr1	1.20E-15	0.52 911 661	0.732	0.46	3.87E-11	phosphatase and actin regulator 1 [Source:MGI Symbol;Acc:MGI:2 659 021]
Cobl	1.33E-15	0.3 222 975	0.218	0.059	4.28E-11	cordon-bleu WH2 repeat [Source:MGI Symbol;Acc:MGI:105 056]
Pcdh15	1.33E-15	0.43 452 653	0.225	0.117	4.29E-11	protocadherin 15 [Source:MGI Symbol;Acc:MGI:1 891 428]
Ralgps1	2.05E-15	0.41 133 638	0.437	0.213	6.62E-11	Ral GEF with PH domain and SH3 binding motif 1 [Source:MGI Symbol;Acc:MGI:1 922 008]
Ppfibp2	2.54E-15	0.35 273 163	0.394	0.158	8.20E-11	PTPRF interacting protein, binding protein 2 (liprin beta 2) [Source:MGI Symbol;Acc:MGI:894 649]
Ube2e2	3.46E-15	0.53 184 922	0.754	0.494	1.12E-10	ubiquitin-conjugating enzyme E2E 2 [Source:MGI Symbol;Acc:MGI:2 384 997]
Olfml1	4.70E-15	0.28 671 091	0.239	0.069	1.52E-10	olfactomedin-like 1 [Source:MGI Symbol;Acc:MGI:2 679 264]
Jam3	7.35E-15	0.2 902 026	0.275	0.075	2.37E-10	junction adhesion molecule 3 [Source:MGI Symbol;Acc:MGI:1 933 825]
Dleu2	9.17E-15	0.49 958 378	0.577	0.253	2.96E-10	deleted in lymphocytic leukemia, 2 [Source:MGI Symbol;Acc:MGI:1 934 030]
Cobll1	1.02E-14	0.33 155 731	0.31	0.097	3.31E-10	Cobl-like 1 [Source:MGI Symbol;Acc:MGI:2 442 894]
Tnfaip6	1.75E-14	0.26 196 361	0.169	0.031	5.66E-10	tumor necrosis factor alpha induced protein 6 [Source:MGI Symbol;Acc:MGI:1 195 266]
Grm7	1.98E-14	0.75 582 859	0.507	0.252	6.38E-10	glutamate receptor, metabotropic 7 [Source:MGI Symbol;Acc:MGI:1 351 344]
Wnk1	2.81E-14	0.44 146 825	0.69	0.476	9.08E-10	WNK lysine deficient protein kinase 1 [Source:MGI Symbol;Acc:MGI:2 442 092]
Galnt7	3.18E-14	0.34 120 701	0.275	0.078	1.03E-09	polypeptide N-acetylgalactosaminyltransferase 7 [Source:MGI Symbol;Acc:MGI:1 349 449]
Dlg1	3.25E-14	0.47 381 536	0.627	0.43	1.05E-09	discs large MAGUK scaffold protein 1 [Source:MGI Symbol;Acc:MGI:107 231]
Ttyh2	4.00E-14	0.30 576 196	0.373	0.168	1.29E-09	tweety family member 2 [Source:MGI Symbol;Acc:MGI:2 157 091]
Kif6	4.36E-14	0.26 052 737	0.197	0.027	1.41E-09	kinesin family member 6 [Source:MGI Symbol;Acc:MGI:1 098 238]
Sytl2	4.73E-14	0.29 329 807	0.232	0.053	1.53E -09	synaptotagmin-like 2 [Source:MGI Symbol;Acc:MGI:1 933 366]
Tmcc1	5.11E-14	0.49 862 924	0.739	0.539	1.65E-09	transmembrane and coiled coil domains 1 [Source:MGI Symbol;Acc:MGI:2 442 368]
Pip4k2a	6.16E-14	0.40 115 848	0.514	0.298	1.99E-09	phosphatidylinositol-5-phosphate 4-kinase, type II, alpha [Source:MGI Symbol;Acc:MGI:1 298 206]
Tulp4	6.52E-14	0.47 631 882	0.634	0.411	2.10E-09	tubby like protein 4 [Source:MGI Symbol;Acc:MGI:1 916 092]
Dpyd	6.89E-14	0.40 904 682	0.423	0.277	2.23E-09	dihydropyrimidine dehydrogenase [Source:MGI Symbol;Acc:MGI:2 139 667]
Rere	8.76E-14	0.46 371 646	0.775	0.603	2.83E-09	arginine glutamic acid dipeptide (RE) repeats [Source:MGI Symbol;Acc:MGI:2 683 486]
Klf13	8.90E-14	0.35 945 172	0.387	0.171	2.87E-09	Kruppel-like factor 13 [Source:MGI Symbol;Acc:MGI:1 354 948]
Picalm	1.00E-13	0.38 008 828	0.465	0.218	3.23E-09	phosphatidylinositol binding clathrin assembly protein [Source:MGI Symbol;Acc:MGI:2 385 902]
Abca2	1.07E-13	0.3 176 473	0.317	0.138	3.47E-09	ATP-binding cassette, sub-family A (ABC1), member 2 [Source:MGI Symbol;Acc:MGI:99 606]
Schip1	1.19E-13	0.41 507 819	0.613	0.361	3.84E-09	schwannomin interacting protein 1 [Source:MGI Symbol;Acc:MGI:1 353 557]
Trim35	1.19E-13	0.33 581 682	0.338	0.143	3.85E-09	tripartite motif-containing 35 [Source:MGI Symbol;Acc:MGI:1 914 104]
Sik3	1.26E-13	0.55 695 036	0.81	0.662	4.08E-09	SIK family kinase 3 [Source:MGI Symbol;Acc:MGI:2 446 296]
Dscam	1.81E-13	0.35 722 675	0.169	0.087	5.84E-09	DS cell adhesion molecule [Source:MGI Symbol;Acc:MGI:1 196 281]
Gas7	1.93E-13	0.31 931 176	0.268	0.101	6.24E-09	growth arrest specific 7 [Source:MGI Symbol;Acc:MGI:1 202 388]
Map4k5	2.07E-13	0.35 137 591	0.387	0.2	6.69E-09	mitogen-activated protein kinase kinase kinase kinase 5 [Source:MGI Symbol;Acc:MGI:1 925 503]
Ptk2	2.31E-13	0.46 037 821	0.718	0.535	7.46E-09	PTK2 protein tyrosine kinase 2 [Source:MGI Symbol;Acc:MGI:95 481]
Rab31	2.35E-13	0.51 587 304	0.577	0.388	7.58E-09	RAB31, member RAS oncogene family [Source:MGI Symbol;Acc:MGI:1 914 603]
Cerk	2.67E-13	0.30 557 382	0.324	0.108	8.62E-09	ceramide kinase [Source:MGI Symbol;Acc:MGI:2 386 052]
Chn2	2.83E-13	0.28 594 957	0.176	0.023	9.13E-09	chimerin 2 [Source:MGI Symbol;Acc:MGI:1 917 243]
Epb41l3	3.41E-13	0.3 103 642	0.317	0.143	1.10E-08	erythrocyte membrane protein band 4.1 like 3 [Source:MGI Symbol;Acc:MGI:103 008]
Naaladl2	3.52E-13	0.59 644 958	0.648	0.349	1.14E-08	N-acetylated alpha-linked acidic dipeptidase-like 2 [Source:MGI Symbol;Acc:MGI:2 685 867]
Fam102a	5.93E-13	0.27 780 908	0.261	0.102	1.92E-08	family with sequence similarity 102, member A [Source:MGI Symbol;Acc:MGI:2 138 935]
Slc38a2	6.77E-13	0.33 572 382	0.31	0.152	2.19E-08	solute carrier family 38, member 2 [Source:MGI Symbol;Acc:MGI:1 915 010]
St6gal1	7.93E-13	0.48 959 382	0.486	0.25	2.56E-08	beta galactoside alpha 2,6 sialyltransferase 1 [Source:MGI Symbol;Acc:MGI:108 470]
Tanc1	8.90E-13	0.26 089 024	0.19	0.063	2.87E-08	tetratricopeptide repeat, ankyrin repeat and coiled-coil containing 1 [Source:MGI Symbol;Acc:MGI:1 914 110]
Rassf2	9.55E-13	0.31 791 016	0.31	0.118	3.08E-08	Ras association (RalGDS/AF-6) domain family member 2 [Source:MGI Symbol;Acc:MGI:2 442 060]
Epn2	1.30E-12	0.40 804 835	0.401	0.243	4.21E-08	epsin 2 [Source:MGI Symbol;Acc:MGI:1 333 766]
Usp31	1.39E-12	0.28 668 209	0.289	0.097	4.49E-08	ubiquitin specific peptidase 31 [Source:MGI Symbol;Acc:MGI:1 923 429]
Enox1	1.48E-12	0.64 119 845	0.641	0.291	4.77E-08	ecto-NOX disulfide-thiol exchanger 1 [Source:MGI Symbol;Acc:MGI:2 444 896]
Pbx3	1.60E-12	0.36 348 669	0.218	0.082	5.18E-08	pre B cell leukemia homeobox 3 [Source:MGI Symbol;Acc:MGI:97 496]
Lrrc8d	1.69E-12	0.26 743 018	0.268	0.067	5.46E-08	leucine rich repeat containing 8D [Source:MGI Symbol;Acc:MGI:1 922 368]
Kctd13	1.89E-12	0.25 910 824	0.232	0.071	6.12E-08	potassium channel tetramerisation domain containing 13 [Source:MGI Symbol;Acc:MGI:1 923 739]
Wipf1	2.10E-12	0.42 212 835	0.472	0.225	6.79E-08	WAS/WASL interacting protein family, member 1 [Source:MGI Symbol;Acc:MGI:2 178 801]
Fmnl2	2.98E-12	0.50 797 646	0.81	0.562	9.62E-08	formin-like 2 [Source:MGI Symbol;Acc:MGI:1 918 659]
Ralyl	3.13E-12	0.37 461 331	0.359	0.197	1.01E-07	RALY RNA binding protein-like [Source:MGI Symbol;Acc:MGI:1 924 147]
Snrnp48	4.35E-12	0.30 167 109	0.38	0.163	1.40E-07	small nuclear ribonucleoprotein 48 (U11/U12) [Source:MGI Symbol;Acc:MGI:1 915 047]
Apbb2	5.86E-12	0.37 732 976	0.5	0.268	1.89E-07	amyloid beta (A4) precursor protein-binding, family B, member 2 [Source:MGI Symbol;Acc:MGI:108 405]
St3gal3	8.35E-12	0.33 765 266	0.387	0.212	2.70E-07	ST3 beta-galactoside alpha-2,3-sialyltransferase 3 [Source:MGI Symbol;Acc:MGI:1 316 659]
Hipk2	9.23E-12	0.42 920 263	0.697	0.481	2.98E-07	homeodomain interacting protein kinase 2 [Source:MGI Symbol;Acc:MGI:1 314 872]
Zfp638	1.00E-11	0.39 071 848	0.606	0.382	3.23E-07	zinc finger protein 638 [Source:MGI Symbol;Acc:MGI:1 203 484]
Bcl2l1	1.02E-11	0.37 843 461	0.387	0.209	3.30E-07	BCL2-like 1 [Source:MGI Symbol;Acc:MGI:88 139]
Plaat3	1.10E-11	0.2 560 622	0.246	0.083	3.54E-07	phospholipase A and acyltransferase 3 [Source:MGI Symbol;Acc:MGI:2 179 715]
Pkp4	1.53E-11	0.42 997 395	0.514	0.279	4.95E-07	plakophilin 4 [Source:MGI Symbol;Acc:MGI:109 281]
Dst	2.29E-11	0.41 891 481	0.838	0.634	7.38E-07	dystonin [Source:MGI Symbol;Acc:MGI:104 627]
Snx1	2.37E-11	0.26 798 752	0.261	0.094	7.65E-07	sorting nexin 1 [Source:MGI Symbol;Acc:MGI:1 928 395]
Itsn2	3.99E-11	0.3 832 391	0.563	0.313	1.29E-06	intersectin 2 [Source:MGI Symbol;Acc:MGI:1 338 049]
Itch	4.64E-11	0.34 894 216	0.556	0.351	1.50E-06	itchy, E3 ubiquitin protein ligase [Source:MGI Symbol;Acc:MGI:1 202 301]
Ankib1	6.01E-11	0.32 551 401	0.38	0.187	1.94E-06	ankyrin repeat and IBR domain containing 1 [Source:MGI Symbol;Acc:MGI:1 918 047]
Rapgef5	7.30E-11	0.28 518 135	0.204	0.078	2.36E-06	Rap guanine nucleotide exchange factor (GEF) 5 [Source:MGI Symbol;Acc:MGI:2 444 365]
Alcam	7.37E-11	0.49 777 924	0.577	0.492	2.38E-06	activated leukocyte cell adhesion molecule [Source:MGI Symbol;Acc:MGI:1 313 266]
Slc25a27	8.64E-11	0.25 283 864	0.254	0.106	2.79E-06	solute carrier family 25, member 27 [Source:MGI Symbol;Acc:MGI:1 921 261]
Ppp2r3a	1.54E-10	0.37 290 247	0.542	0.368	4.96E-06	protein phosphatase 2, regulatory subunit B'', alpha [Source:MGI Symbol;Acc:MGI:2 442 104]
Foxp1	1.58E-10	0.36 946 891	0.634	0.446	5.11E-06	forkhead box P1 [Source:MGI Symbol;Acc:MGI:1 914 004]
Sh3kbp1	1.76E-10	0.32 577 313	0.331	0.145	5.69E-06	SH3-domain kinase binding protein 1 [Source:MGI Symbol;Acc:MGI:1 889 583]
Oxr1	2.01E-10	0.43 543 651	0.648	0.402	6.48E-06	oxidation resistance 1 [Source:MGI Symbol;Acc:MGI:2 179 326]
Ppp1r21	2.34E-10	0.26 332 997	0.289	0.129	7.54E-06	protein phosphatase 1, regulatory subunit 21 [Source:MGI Symbol;Acc:MGI:1 921 075]
Elavl3	2.44E-10	0.30 396 832	0.303	0.143	7.88E-06	ELAV like RNA binding protein 3 [Source:MGI Symbol;Acc:MGI:109 157]
Clic4	2.45E-10	0.28 194 369	0.408	0.232	7.90E-06	chloride intracellular channel 4 (mitochondrial) [Source:MGI Symbol;Acc:MGI:1 352 754]
Dnajc6	2.62E-10	0.29 955 562	0.239	0.109	8.44E-06	DnaJ heat shock protein family (Hsp40) member C6 [Source:MGI Symbol;Acc:MGI:1 919 935]
Ncam1	2.62E-10	0.3 620 721	0.873	0.779	8.45E-06	neural cell adhesion molecule 1 [Source:MGI Symbol;Acc:MGI:97 281]
Gm48512	2.85E-10	0.31 683 148	0.31	0.106	9.19E-06	predicted gene, 48 512 [Source:MGI Symbol;Acc:MGI:6 098 046]
Usp54	7.29E-10	0.35 238 725	0.458	0.317	2.35E-05	ubiquitin specific peptidase 54 [Source:MGI Symbol;Acc:MGI:1 926 037]
Mtss1	9.29E-10	0.25 566 784	0.261	0.114	3.00E-05	MTSS I-BAR domain containing 1 [Source:MGI Symbol;Acc:MGI:2 384 818]
Kat2b	1.19E-09	0.31 444 963	0.423	0.224	3.85E-05	K(lysine) acetyltransferase 2B [Source:MGI Symbol;Acc:MGI:1 343 094]
Dpysl2	1.24E-09	0.3 221 776	0.549	0.343	4.00E-05	dihydropyrimidinase-like 2 [Source:MGI Symbol;Acc:MGI:1 349 763]
Gna12	1.28E-09	0.29 101 118	0.38	0.175	4.14E-05	guanine nucleotide binding protein, alpha 12 [Source:MGI Symbol;Acc:MGI:95 767]
Ywhaq	1.52E-09	0.30 001 321	0.415	0.221	4.92E-05	tyrosine 3-monooxygenase/tryptophan 5-monooxygenase activation protein theta [Source:MGI Symbol;Acc:MGI:891 963]
Kif1b	1.55E-09	0.36 174 404	0.923	0.81	5.01E-05	kinesin family member 1B [Source:MGI Symbol;Acc:MGI:108 426]
5031439G07Rik	1.55E-09	0.30 489 788	0.535	0.389	5.02E-05	RIKEN cDNA 5031439G07 gene [Source:MGI Symbol;Acc:MGI:2 444 899]
Nrbp2	1.59E-09	0.32 356 984	0.394	0.215	5.12E-05	nuclear receptor binding protein 2 [Source:MGI Symbol;Acc:MGI:2 385 017]
Ncoa3	1.61E-09	0.25 337 761	0.275	0.104	5.19E-05	nuclear receptor coactivator 3 [Source:MGI Symbol;Acc:MGI:1 276 535]
Otud7a	1.77E-09	0.50 197 617	0.704	0.473	5.73E-05	OTU domain containing 7A [Source:MGI Symbol;Acc:MGI:2 158 505]
Daam1	1.89E-09	0.38 269 952	0.563	0.404	6.11E-05	dishevelled associated activator of morphogenesis 1 [Source:MGI Symbol;Acc:MGI:1 914 596]
Ankrd28	4.30E-09	0.34 478 589	0.556	0.365	0.00 013 897	ankyrin repeat domain 28 [Source:MGI Symbol;Acc:MGI:2 145 661]
Fryl	4.33E-09	0.32 061 006	0.57	0.332	0.00 013 975	FRY like transcription coactivator [Source:MGI Symbol;Acc:MGI:1 919 563]
Ncam2	5.63E-09	0.52 172 685	0.754	0.557	0.00 018 185	neural cell adhesion molecule 2 [Source:MGI Symbol;Acc:MGI:97 282]
Pakap.1	5.86E-09	0.25 161 446	0.268	0.101	0.0 001 892	NA
Cpeb2	6.71E-09	0.27 668 338	0.366	0.254	0.00 021 677	cytoplasmic polyadenylation element binding protein 2 [Source:MGI Symbol;Acc:MGI:2 442 640]
Pld1	7.92E-09	0.26 934 365	0.261	0.093	0.00 025 557	phospholipase D1 [Source:MGI Symbol;Acc:MGI:109 585]
Retreg1	8.48E-09	0.28 313 566	0.324	0.126	0.0 002 738	reticulophagy regulator 1 [Source:MGI Symbol;Acc:MGI:1 913 520]
Apod	8.62E-09	0.27 079 684	0.345	0.213	0.0 002 784	apolipoprotein D [Source:MGI Symbol;Acc:MGI:88 056]
Atp11a	1.01E-08	0.25 199 273	0.282	0.151	0.00 032 617	ATPase, class VI, type 11A [Source:MGI Symbol;Acc:MGI:1 354 735]
Sema6a	1.31E-08	0.30 897 174	0.296	0.102	0.00 042 137	sema domain, transmembrane domain (TM), and cytoplasmic domain, (semaphorin) 6A [Source:MGI Symbol;Acc:MGI:1 203 727]
2610035D17Rik	1.51E-08	0.40 048 971	0.577	0.428	0.00 048 781	RIKEN cDNA 2610035D17 gene [Source:MGI Symbol;Acc:MGI:1 919 636]
Baz2b	1.57E-08	0.37 303 625	0.697	0.551	0.00 050 559	bromodomain adjacent to zinc finger domain, 2B [Source:MGI Symbol;Acc:MGI:2 442 782]
Wdr20	2.34E-08	0.25 520 223	0.338	0.159	0.00 075 438	WD repeat domain 20 [Source:MGI Symbol;Acc:MGI:1 916 891]
Cttnbp2	2.40E-08	0.32 095 607	0.458	0.266	0.0 007 738	cortactin binding protein 2 [Source:MGI Symbol;Acc:MGI:1 353 467]
Osbpl1a	2.57E-08	0.31 900 489	0.493	0.325	0.00 082 996	oxysterol binding protein-like 1A [Source:MGI Symbol;Acc:MGI:1 927 551]
Abhd17b	2.70E-08	0.29 402 589	0.465	0.29	0.00 087 174	abhydrolase domain containing 17B [Source:MGI Symbol;Acc:MGI:1 917 816]
Rnf13	3.01E-08	0.27 892 308	0.423	0.229	0.000 972	ring finger protein 13 [Source:MGI Symbol;Acc:MGI:1 346 341]
Tmem132b	4.93E-08	0.26 716 194	0.268	0.101	0.00 159 086	transmembrane protein 132B [Source:MGI Symbol;Acc:MGI:3 609 245]
Gnao1	5.42E-08	0.3 966 222	0.838	0.654	0.00 175 013	guanine nucleotide binding protein, alpha O [Source:MGI Symbol;Acc:MGI:95 775]
Pde1c	5.66E-08	0.25 711 314	0.232	0.051	0.00 182 824	phosphodiesterase 1C [Source:MGI Symbol;Acc:MGI:108 413]
Pvt1	7.44E-08	0.25 652 958	0.31	0.154	0.00 240 229	Pvt1 oncogene [Source:MGI Symbol;Acc:MGI:97 824]
Pard3	8.43E-08	0.3 133 286	0.592	0.454	0.00 272 182	par-3 family cell polarity regulator [Source:MGI Symbol;Acc:MGI:2 135 608]
Malat1	8.64E-08	0.26 747 411	1	1	0.00 279 021	metastasis associated lung adenocarcinoma transcript 1 (non-coding RNA) [Source:MGI Symbol;Acc:MGI:1 919 539]
Dcc	9.55E-08	0.34 680 795	0.5	0.294	0.00 308 213	deleted in colorectal carcinoma [Source:MGI Symbol;Acc:MGI:94 869]
Zcchc7	1.00E-07	0.32 309 135	0.69	0.5	0.00 323 528	zinc finger, CCHC domain containing 7 [Source:MGI Symbol;Acc:MGI:2 442 912]
Limch1	1.08E-07	0.35 675 448	0.739	0.632	0.00 349 991	LIM and calponin homology domains 1 [Source:MGI Symbol;Acc:MGI:1 924 819]

Interestingly, Foxp1, a TF with major roles in gene transcription regulation of tissue and cell type-specificity, and involved in subtype identity coordination, migration, and organization of motor neurons [75–77], showed a diverse expression across all groups, but it was significantly upregulated in AG1 of motor cortex. Additional TFs found significantly upregulated included Creb5, Litaf, Nfe2l3, Tulp4, Tcf12, Rere, and Pbx3.

Tmem117 transmembrane protein was found expressed uniquely in AG1 from spinal cord, being both group 1 and tissue specific marker.

Achaete-scute family bHLH TF 1 (Ascl1) gene was significantly upregulated in AG1. Magnusson *et al.* demonstrated that Ascl1 + mature astrocytes take on a progenitor-cell like role and become neurogenic after stroke in adult mice [78]. Similar small populations related with progenitor cells have been described in rodent hippocampus, linked with GFAP expression [79], or defined by high expression of Frzb, Ascl1, and Slc1a3 [80], although there is no consensus if misidentification of progenitors in adult brain could be due to de-differentiation of mature neurons [81].

To extend the understanding of these findings in a multicellular scenario and how AG1 might interact with specific sets of neurons, we analyzed cell-cell communication patterns and signaling pathways based on ligand-receptor interactions using the motor cortex expression data from the tree mice, between neuron clusters and the three different AG. Results show SEMA6 signaling and communication pathway exclusively active between AG1 and specific neuron clusters 3, 4, and 8 (Fig. 4C–E). SEMA6 signaling network, with known roles in CNS development, neuron migration and axon guidance [82–84], was shown to be active among AG1 with a signaling sender role mediated by expression of Sema6a in AG1 (Fig. 4C and E). All AG1, AG2 and AG3 showed moderated expression of receptors Nrg1 and Nrg3, but Erbb4 receptor was exclusively expressed in AG1. AG3 had exclusively a sender role in the NRG signaling pathway, AG1 on the contrary had a receiver role. Neuregulins (NRGs) are a large family of growth factors that stimulate ERBB receptor tyrosine kinases and are critical for the assembly of the GABAergic circuitry including interneuron migration, neural differentiation, axon and dendrite development, myelination and synapse formation [85–88].

These gene markers and findings have been previously described in scientific literature, in which they have been linked with mature astrocytes promoting a neurogenic environment or acquiring progenitor-cell like properties [78, 89–94].

### GFAP-positive astrocyte population

Astrocyte group 2 (AG2) yielded 223 genes that were differentially upregulated (*P*-value < 0.005, Table 2). Within this list, Gfap was among the top, particularly in the motor cortex, where it was almost exclusively expressed in AG2 cell clusters. Additional markers of astrocyte activation [67] were found upregulated on the AG2 population, including Aldoc, Stat3, and C3.

**Table 2.** Differentially upregulated genes (*P* value < 0.005) in AG2 in comparison with AG1 and AG3

Gene	p_val	avg_log2FC	pct.1	pct.2	p_val_adj	description
Ablim2	0	2.01 268 205	0.592	0.142	0	actin-binding LIM protein 2 [Source:MGI Symbol;Acc:MGI:2 385 758]
Gpc5	0	−2.4215435	0.523	0.978	0	glypican 5 [Source:MGI Symbol;Acc:MGI:1 194 894]
Trpm3	0	−2.7772547	0.475	0.931	0	transient receptor potential cation channel, subfamily M, member 3 [Source:MGI Symbol;Acc:MGI:2 443 101]
Lsamp	5.69E-249	−1.4851803	0.993	1	1.84E-244	limbic system-associated membrane protein [Source:MGI Symbol;Acc:MGI:1 261 760]
Plcb1	6.73E-181	−1.740611	0.67	0.946	2.17E-176	phospholipase C, beta 1 [Source:MGI Symbol;Acc:MGI:97 613]
6330411D24Rik	6.01E-180	2.09 342 464	0.401	0.022	1.94E-175	RIKEN cDNA 6330411D24 gene [Source:MGI Symbol;Acc:MGI:1 917 975]
Kcnd2	5.20E-170	−1.9350475	0.373	0.706	1.68E-165	potassium voltage-gated channel, Shal-related family, member 2 [Source:MGI Symbol;Acc:MGI:102 663]
Prune2	2.77E-168	1.331 272	0.614	0.135	8.94E-164	prune homolog 2 [Source:MGI Symbol;Acc:MGI:1 925 004]
Sorbs2	1.02E-167	1.50 461 418	0.618	0.173	3.30E-163	sorbin and SH3 domain containing 2 [Source:MGI Symbol;Acc:MGI:1 924 574]
Kirrel3	6.70E-158	−1.7929888	0.401	0.879	2.16E-153	kirre like nephrin family adhesion molecule 3 [Source:MGI Symbol;Acc:MGI:1 914 953]
Gfap	7.52E-154	1.81 373 942	0.666	0.215	2.43E-149	glial fibrillary acidic protein [Source:MGI Symbol;Acc:MGI:95 697]
Mdga2	4.23E-152	−1.3081606	0.811	0.974	1.36E-147	MAM domain containing glycosylphosphatidylinositol anchor 2 [Source:MGI Symbol;Acc:MGI:2 444 706]
Nrxn1	2.30E-143	−1.2740743	0.863	0.998	7.42E-139	neurexin I [Source:MGI Symbol;Acc:MGI:1 096 391]
Gm3764	7.00E-142	−1.2598406	0.727	0.937	2.26E-137	predicted gene 3764 [Source:MGI Symbol;Acc:MGI:3 781 938]
Grik2	4.07E-133	1.35 293 147	0.796	0.463	1.31E-128	glutamate receptor, ionotropic, kainate 2 (beta 2) [Source:MGI Symbol;Acc:MGI:95 815]
Cacnb2	5.58E-130	1.4 550 695	0.644	0.257	1.80E-125	calcium channel, voltage-dependent, beta 2 subunit [Source:MGI Symbol;Acc:MGI:894 644]
Ccdc148	6.30E-122	1.04 581 674	0.401	0.055	2.04E-117	coiled-coil domain containing 148 [Source:MGI Symbol;Acc:MGI:3 039 583]
Pkp4	1.87E-121	1.16 887 237	0.61	0.233	6.05E-117	plakophilin 4 [Source:MGI Symbol;Acc:MGI:109 281]
Gm12239	1.17E-119	−1.5630729	0.15	0.566	3.78E-115	predicted gene 12 239 [Source:MGI Symbol;Acc:MGI:3 651 547]
Adamtsl1	1.78E-118	1.0 194 373	0.369	0.038	5.76E-114	ADAMTS-like 1 [Source:MGI Symbol;Acc:MGI:1 924 989]
A2m	1.89E-109	1.16 768 979	0.421	0.065	6.11E-105	alpha-2-macroglobulin [Source:MGI Symbol;Acc:MGI:2 449 119]
Rora	3.95E-109	−1.0725217	0.952	0.989	1.28E-104	RAR-related orphan receptor alpha [Source:MGI Symbol;Acc:MGI:104 661]
Pcdh9	4.52E-109	−1.0889765	0.944	0.992	1.46E-104	protocadherin 9 [Source:MGI Symbol;Acc:MGI:1 306 801]
Slc38a1	6.45E-108	1.18 683 573	0.629	0.236	2.08E-103	solute carrier family 38, member 1 [Source:MGI Symbol;Acc:MGI:2 145 895]
Cadps	3.49E-101	1.07 111 948	0.681	0.305	1.13E-96	Ca2+-dependent secretion activator [Source:MGI Symbol;Acc:MGI:1 350 922]
Pitpnc1	5.38E-101	−1.2086183	0.694	0.913	1.74E-96	phosphatidylinositol transfer protein, cytoplasmic 1 [Source:MGI Symbol;Acc:MGI:1 919 045]
Kcnj3	3.06E-98	0.90 152 953	0.562	0.166	9.89E-94	potassium inwardly-rectifying channel, subfamily J, member 3 [Source:MGI Symbol;Acc:MGI:104 742]
Gm14964	1.15E-96	0.76 450 618	0.508	0.159	3.70E-92	predicted gene 14 964 [Source:MGI Symbol;Acc:MGI:3 641 621]
Ccdc3	5.80E-96	0.76 885 968	0.341	0.024	1.87E-91	coiled-coil domain containing 3 [Source:MGI Symbol;Acc:MGI:1 921 436]
Asap3	1.18E-93	0.63 349 421	0.343	0.027	3.82E-89	ArfGAP with SH3 domain, ankyrin repeat and PH domain 3 [Source:MGI Symbol;Acc:MGI:2 684 986]
Atp1a2	1.41E-92	−0.9512626	0.779	0.932	4.56E-88	ATPase, Na+/K + transporting, alpha 2 polypeptide [Source:MGI Symbol;Acc:MGI:88 106]
Gm4876	4.73E-88	0.82 426 118	0.477	0.131	1.53E-83	predicted gene 4876 [Source:MGI Symbol;Acc:MGI:3 647 654]
Car10	9.87E-88	−1.1700691	0.252	0.479	3.19E-83	carbonic anhydrase 10 [Source:MGI Symbol;Acc:MGI:1 919 855]
Ldb2	1.61E-86	0.78 138 886	0.462	0.119	5.19E-82	LIM domain binding 2 [Source:MGI Symbol;Acc:MGI:894 670]
Arhgef4	6.30E-86	1.03 026 893	0.772	0.564	2.03E-81	Rho guanine nucleotide exchange factor (GEF) 4 [Source:MGI Symbol;Acc:MGI:2 442 507]
Prkag2	6.94E-85	0.77 822 608	0.492	0.156	2.24E-80	protein kinase, AMP-activated, gamma 2 non-catalytic subunit [Source:MGI Symbol;Acc:MGI:1 336 153]
Col23a1	8.05E-85	1.11 579 567	0.469	0.11	2.60E-80	collagen, type XXIII, alpha 1 [Source:MGI Symbol;Acc:MGI:2 653 243]
Brinp3	4.08E-84	−1.0567625	0.267	0.698	1.32E-79	bone morphogenetic protein/retinoic acid inducible neural specific 3 [Source:MGI Symbol;Acc:MGI:2 443 035]
Myoc	9.22E-83	0.80 203 131	0.273	0.012	2.98E-78	myocilin [Source:MGI Symbol;Acc:MGI:1 202 864]
Pdzrn3	4.51E-82	0.69 065 993	0.434	0.1	1.46E-77	PDZ domain containing RING finger 3 [Source:MGI Symbol;Acc:MGI:1 933 157]
C4b	9.92E-82	0.67 373 866	0.401	0.073	3.20E-77	complement component 4B (Chido blood group) [Source:MGI Symbol;Acc:MGI:88 228]
Gm20713	1.04E-81	−1.1671095	0.148	0.71	3.35E-77	predicted gene 20 713 [Source:MGI Symbol;Acc:MGI:5 313 160]
Cadm2	9.70E-81	−0.8677423	0.887	0.991	3.13E-76	cell adhesion molecule 2 [Source:MGI Symbol;Acc:MGI:2 442 722]
Cobll1	1.01E-80	0.69 941 561	0.38	0.058	3.26E-76	Cobl-like 1 [Source:MGI Symbol;Acc:MGI:2 442 894]
Tmem108	7.84E-79	0.68 764 555	0.325	0.048	2.53E-74	transmembrane protein 108 [Source:MGI Symbol;Acc:MGI:1 932 411]
Slc6a11	1.58E-78	−0.9528088	0.26	0.577	5.11E-74	solute carrier family 6 (neurotransmitter transporter, GABA), member 11 [Source:MGI Symbol;Acc:MGI:95 630]
Gria2	8.90E-78	−1.1708527	0.401	0.765	2.87E-73	glutamate receptor, ionotropic, AMPA2 (alpha 2) [Source:MGI Symbol;Acc:MGI:95 809]
Ptch1	1.21E-77	−1.0379565	0.178	0.572	3.91E-73	patched 1 [Source:MGI Symbol;Acc:MGI:105 373]
Nhsl1	2.00E-77	−1.0084889	0.362	0.724	6.47E-73	NHS-like 1 [Source:MGI Symbol;Acc:MGI:106 390]
Cd44	3.84E-75	0.61 517 867	0.289	0.022	1.24E-70	CD44 antigen [Source:MGI Symbol;Acc:MGI:88 338]
Adgrb3	8.83E-75	−0.9307768	0.876	0.956	2.85E-70	adhesion G protein-coupled receptor B3 [Source:MGI Symbol;Acc:MGI:2 441 837]
Man1c1	2.14E-73	0.63 698 922	0.336	0.063	6.91E-69	mannosidase, alpha, class 1C, member 1 [Source:MGI Symbol;Acc:MGI:2 446 214]
Prkca	4.62E-71	0.97 231 792	0.8	0.561	1.49E-66	protein kinase C, alpha [Source:MGI Symbol;Acc:MGI:97 595]
Sntg2	1.58E-70	0.49 453 624	0.282	0.015	5.10E-66	syntrophin, gamma 2 [Source:MGI Symbol;Acc:MGI:1 919 541]
Sulf2	1.19E-69	0.53 943 003	0.291	0.044	3.85E-65	sulfatase 2 [Source:MGI Symbol;Acc:MGI:1 919 293]
Glis3	2.10E-69	0.81 574 381	0.79	0.528	6.77E-65	GLIS family zinc finger 3 [Source:MGI Symbol;Acc:MGI:2 444 289]
Nrp2	8.88E-69	0.52 318 158	0.323	0.045	2.87E-64	neuropilin 2 [Source:MGI Symbol;Acc:MGI:1 100 492]
Reep1	7.50E-67	0.64 992 347	0.343	0.069	2.42E-62	receptor accessory protein 1 [Source:MGI Symbol;Acc:MGI:1 098 827]
Slc7a10	5.38E-65	−1.1162491	0.065	0.552	1.74E-60	solute carrier family 7 (cationic amino acid transporter, y + system), member 10 [Source:MGI Symbol;Acc:MGI:1 858 261]
Pde7b	6.13E-65	−0.9965179	0.273	0.701	1.98E-60	phosphodiesterase 7B [Source:MGI Symbol;Acc:MGI:1 352 752]
Ctnna2	2.42E-63	1.08 054 998	0.844	0.676	7.81E-59	catenin (cadherin associated protein), alpha 2 [Source:MGI Symbol;Acc:MGI:88 275]
Csmd1	7.81E-63	0.67 950 176	0.577	0.395	2.52E-58	CUB and Sushi multiple domains 1 [Source:MGI Symbol;Acc:MGI:2 137 383]
Sorbs1	1.00E-62	0.95 190 338	0.829	0.638	3.24E-58	sorbin and SH3 domain containing 1 [Source:MGI Symbol;Acc:MGI:700 014]
Aebp1	1.73E-62	0.45 464 351	0.258	0.013	5.59E-58	AE binding protein 1 [Source:MGI Symbol;Acc:MGI:1 197 012]
Sema3a	1.11E-60	0.54 359 609	0.252	0.03	3.59E-56	sema domain, immunoglobulin domain (Ig), short basic domain, secreted, (semaphorin) 3A [Source:MGI Symbol;Acc:MGI:107 558]
Rgs7	2.21E-60	−1.0127883	0.243	0.642	7.14E-56	regulator of G protein signaling 7 [Source:MGI Symbol;Acc:MGI:1 346 089]
Slc8a1	5.29E-60	0.95 401 456	0.521	0.247	1.71E-55	solute carrier family 8 (sodium/calcium exchanger), member 1 [Source:MGI Symbol;Acc:MGI:107 956]
Synpo2	5.36E-60	0.82 634 096	0.497	0.165	1.73E-55	synaptopodin 2 [Source:MGI Symbol;Acc:MGI:2 153 070]
Dock4	7.62E-60	−0.7963306	0.579	0.81	2.46E-55	dedicator of cytokinesis 4 [Source:MGI Symbol;Acc:MGI:1 918 006]
C530044C16Rik	1.87E-59	0.4 873 999	0.234	0.012	6.04E-55	RIKEN cDNA C530044C16 gene [Source:MGI Symbol;Acc:MGI:2 443 109]
Tagln3	4.73E-59	0.61 896 383	0.577	0.299	1.53E-54	transgelin 3 [Source:MGI Symbol;Acc:MGI:1 926 784]
Adgrl3	1.51E-57	−0.7136463	0.779	0.899	4.88E-53	adhesion G protein-coupled receptor L3 [Source:MGI Symbol;Acc:MGI:2 441 950]
Cpne8	2.52E-56	0.41 103 057	0.262	0.037	8.13E-52	copine VIII [Source:MGI Symbol;Acc:MGI:1 914 121]
Phyhd1	4.30E-56	0.6 780 051	0.534	0.228	1.39E-51	phytanoyl-CoA dioxygenase domain containing 1 [Source:MGI Symbol;Acc:MGI:3 612 860]
Gria4	4.97E-56	−0.8576014	0.206	0.42	1.60E-51	glutamate receptor, ionotropic, AMPA4 (alpha 4) [Source:MGI Symbol;Acc:MGI:95 811]
Padi2	8.82E-56	0.61 810 846	0.423	0.128	2.85E-51	peptidyl arginine deiminase, type II [Source:MGI Symbol;Acc:MGI:1 338 892]
Robo2	1.23E-54	0.9 155 612	0.787	0.569	3.98E-50	roundabout guidance receptor 2 [Source:MGI Symbol;Acc:MGI:1 890 110]
Ccser1	1.27E-54	0.80 908 857	0.473	0.287	4.09E-50	coiled-coil serine rich 1 [Source:MGI Symbol;Acc:MGI:3 045 354]
St6galnac3	2.01E-54	1.53 920 755	0.54	0.139	6.49E-50	ST6 (alpha-N-acetyl-neuraminyl-2,3-beta-galactosyl-1,3)-N-acetylgalactosaminide alpha-2,6-sialyltransferase 3 [Source:MGI Symbol;Acc:MGI:1 341 828]
Cdh10	8.08E-54	−0.7785671	0.445	0.736	2.61E-49	cadherin 10 [Source:MGI Symbol;Acc:MGI:107 436]
Dok6	1.84E-53	0.70 032 164	0.462	0.163	5.93E-49	docking protein 6 [Source:MGI Symbol;Acc:MGI:3 639 495]
Smad6	3.72E-53	0.34 957 981	0.247	0.034	1.20E-48	SMAD family member 6 [Source:MGI Symbol;Acc:MGI:1 336 883]
Aqp4	6.40E-53	0.78 867 024	0.701	0.403	2.07E-48	aquaporin 4 [Source:MGI Symbol;Acc:MGI:107 387]
Grm7	9.42E-51	0.88 253 317	0.462	0.229	3.04E-46	glutamate receptor, metabotropic 7 [Source:MGI Symbol;Acc:MGI:1 351 344]
Dgki	6.91E-50	0.76 957 449	0.573	0.244	2.23E-45	diacylglycerol kinase, iota [Source:MGI Symbol;Acc:MGI:2 443 430]
Tex11	1.04E-49	0.36 512 003	0.215	0.012	3.35E-45	testis expressed gene 11 [Source:MGI Symbol;Acc:MGI:1 933 237]
Ahnak	1.08E-48	0.32 270 059	0.234	0.027	3.48E-44	AHNAK nucleoprotein (desmoyokin) [Source:MGI Symbol;Acc:MGI:1 316 648]
Trp63	2.37E-48	−0.9180372	0.035	0.318	7.64E-44	transformation related protein 63 [Source:MGI Symbol;Acc:MGI:1 330 810]
Tnc	2.67E-48	0.37 489 867	0.234	0.023	8.61E-44	tenascin C [Source:MGI Symbol;Acc:MGI:101 922]
Reln	3.62E-48	0.6 459 527	0.397	0.109	1.17E-43	reelin [Source:MGI Symbol;Acc:MGI:103 022]
Pxdn	3.76E-48	0.4 112 221	0.293	0.072	1.21E-43	peroxidasin [Source:MGI Symbol;Acc:MGI:1 916 925]
Atp13a4	7.34E-48	−0.6669755	0.145	0.486	2.37E-43	ATPase type 13A4 [Source:MGI Symbol;Acc:MGI:1 924 456]
Cacnb4	9.29E-47	0.71 396 113	0.484	0.238	3.00E-42	calcium channel, voltage-dependent, beta 4 subunit [Source:MGI Symbol;Acc:MGI:103 301]
Adamts9	2.03E-46	0.68 554 096	0.499	0.202	6.57E-42	a disintegrin-like and metallopeptidase (reprolysin type) with thrombospondin type 1 motif, 9 [Source:MGI Symbol;Acc:MGI:1 916 320]
Tmtc2	3.43E-46	−0.7329488	0.243	0.576	1.11E-41	transmembrane and tetratricopeptide repeat containing 2 [Source:MGI Symbol;Acc:MGI:1 914 057]
Limch1	7.50E-46	−0.6811558	0.395	0.681	2.42E-41	LIM and calponin homology domains 1 [Source:MGI Symbol;Acc:MGI:1 924 819]
Grid2	1.96E-45	−0.6694846	0.709	0.906	6.34E-41	glutamate receptor, ionotropic, delta 2 [Source:MGI Symbol;Acc:MGI:95 813]
Cadm1	2.29E-45	−0.8065897	0.549	0.835	7.40E-41	cell adhesion molecule 1 [Source:MGI Symbol;Acc:MGI:1 889 272]
Sema6a	1.15E-44	0.44 205 131	0.315	0.074	3.73E-40	sema domain, transmembrane domain (TM), and cytoplasmic domain, (semaphorin) 6A [Source:MGI Symbol;Acc:MGI:1 203 727]
Ggta1	3.51E-44	0.29 853 878	0.193	0.018	1.13E-39	glycoprotein galactosyltransferase alpha 1, 3 [Source:MGI Symbol;Acc:MGI:95 704]
Npas3	5.45E-44	−0.5712718	0.944	0.981	1.76E-39	neuronal PAS domain protein 3 [Source:MGI Symbol;Acc:MGI:1 351 610]
Garnl3	7.71E-44	0.42 029 537	0.306	0.093	2.49E-39	GTPase activating RANGAP domain-like 3 [Source:MGI Symbol;Acc:MGI:2 139 309]
Kcnma1	1.92E-43	−0.7810046	0.31	0.667	6.19E-39	potassium large conductance calcium-activated channel, subfamily M, alpha member 1 [Source:MGI Symbol;Acc:MGI:99 923]
Auts2	2.67E-43	−0.700036	0.735	0.913	8.63E-39	autism susceptibility candidate 2 [Source:MGI Symbol;Acc:MGI:1 919 847]
Fmn1	2.69E-43	0.79 828 693	0.427	0.198	8.69E-39	formin 1 [Source:MGI Symbol;Acc:MGI:101 815]
Disp3	2.97E-43	0.4 166 997	0.252	0.079	9.59E-39	dispatched RND transporter family member 3 [Source:MGI Symbol;Acc:MGI:2 444 403]
Grin3a	4.31E-43	0.59 174 305	0.386	0.136	1.39E-38	glutamate receptor ionotropic, NMDA3A [Source:MGI Symbol;Acc:MGI:1 933 206]
Sema6d	8.82E-43	0.98 719 666	0.711	0.533	2.85E-38	sema domain, transmembrane domain (TM), and cytoplasmic domain, (semaphorin) 6D [Source:MGI Symbol;Acc:MGI:2 387 661]
Pcdh7	1.43E-42	−0.6289841	0.735	0.874	4.62E-38	protocadherin 7 [Source:MGI Symbol;Acc:MGI:1 860 487]
Ank2	1.64E-42	0.95 150 824	0.939	0.955	5.30E-38	ankyrin 2, brain [Source:MGI Symbol;Acc:MGI:88 025]
Mast4	1.87E-41	−0.6718364	0.44	0.716	6.04E-37	microtubule associated serine/threonine kinase family member 4 [Source:MGI Symbol;Acc:MGI:1 918 885]
Slc4a4	2.81E-41	−0.5501395	0.852	0.919	9.08E-37	solute carrier family 4 (anion exchanger), member 4 [Source:MGI Symbol;Acc:MGI:1 927 555]
Ano1	5.02E-41	0.46 720 261	0.215	0.003	1.62E-36	anoctamin 1, calcium activated chloride channel [Source:MGI Symbol;Acc:MGI:2 142 149]
Cmya5	8.05E-41	0.45 054 959	0.341	0.097	2.60E-36	cardiomyopathy associated 5 [Source:MGI Symbol;Acc:MGI:1 923 719]
Dner	1.62E-40	0.46 188 889	0.399	0.176	5.22E-36	delta/notch-like EGF repeat containing [Source:MGI Symbol;Acc:MGI:2 152 889]
Gpld1	2.60E-40	−0.6378762	0.08	0.385	8.38E-36	glycosylphosphatidylinositol specific phospholipase D1 [Source:MGI Symbol;Acc:MGI:106 604]
Negr1	2.65E-40	−0.6970982	0.62	0.852	8.56E-36	neuronal growth regulator 1 [Source:MGI Symbol;Acc:MGI:2 444 846]
Anxa2	1.10E-39	0.27 925 372	0.176	0.012	3.54E-35	annexin A2 [Source:MGI Symbol;Acc:MGI:88 246]
Vav3	2.04E-39	−0.686609	0.091	0.439	6.58E-35	vav 3 oncogene [Source:MGI Symbol;Acc:MGI:1 888 518]
Airn	2.14E-39	0.4 276 238	0.232	0.057	6.90E-35	antisense Igf2r RNA [Source:MGI Symbol;Acc:MGI:1 353 471]
Mgat4a	2.28E-39	0.32 616 976	0.217	0.039	7.37E-35	mannoside acetylglucosaminyltransferase 4, isoenzyme A [Source:MGI Symbol;Acc:MGI:2 662 992]
Enox1	4.40E-39	0.73 574 687	0.499	0.274	1.42E-34	ecto-NOX disulfide-thiol exchanger 1 [Source:MGI Symbol;Acc:MGI:2 444 896]
Frmd4a	6.76E-39	−0.7555608	0.735	0.929	2.18E-34	FERM domain containing 4A [Source:MGI Symbol;Acc:MGI:1 919 850]
Eps8	7.63E-39	−0.5995504	0.219	0.523	2.46E-34	epidermal growth factor receptor pathway substrate 8 [Source:MGI Symbol;Acc:MGI:104 684]
Gm10635	8.27E-39	0.4 519 927	0.219	0.022	2.67E-34	predicted gene 10 635 [Source:MGI Symbol;Acc:MGI:3 641 740]
Atoh8	9.59E-39	0.28 185 611	0.165	0.008	3.10E-34	atonal bHLH transcription factor 8 [Source:MGI Symbol;Acc:MGI:1 918 343]
Slc16a2	3.09E-38	0.31 372 704	0.228	0.051	9.98E-34	solute carrier family 16 (monocarboxylic acid transporters), member 2 [Source:MGI Symbol;Acc:MGI:1 203 732]
Tab2	8.33E-38	0.49 912 784	0.521	0.257	2.69E-33	TGF-beta activated kinase 1/MAP3K7 binding protein 2 [Source:MGI Symbol;Acc:MGI:1 915 902]
Gm11099	9.08E-38	0.33 560 931	0.156	0.007	2.93E-33	predicted gene 11 099 [Source:MGI Symbol;Acc:MGI:3 779 335]
Itih3	9.39E-38	−0.6159658	0.074	0.332	3.03E-33	inter-alpha trypsin inhibitor, heavy chain 3 [Source:MGI Symbol;Acc:MGI:96 620]
P3h2	1.14E-36	0.29 550 061	0.178	0.018	3.69E-32	prolyl 3-hydroxylase 2 [Source:MGI Symbol;Acc:MGI:2 146 663]
Cables1	1.22E-36	−0.588048	0.145	0.459	3.95E-32	CDK5 and Abl enzyme substrate 1 [Source:MGI Symbol;Acc:MGI:1 927 065]
4930402H24Rik	1.48E-36	0.65 012 509	0.855	0.834	4.78E-32	NA
Syne1	1.85E-36	−0.5532005	0.748	0.878	5.97E-32	spectrin repeat containing, nuclear envelope 1 [Source:MGI Symbol;Acc:MGI:1 927 152]
Grm3	3.44E-36	−0.8946045	0.334	0.583	1.11E-31	glutamate receptor, metabotropic 3 [Source:MGI Symbol;Acc:MGI:1 351 340]
Eya4	4.53E-36	0.43 761 345	0.31	0.102	1.46E-31	EYA transcriptional coactivator and phosphatase 4 [Source:MGI Symbol;Acc:MGI:1 337 104]
Mamdc2	3.19E-35	0.27 013 547	0.156	0.007	1.03E-30	MAM domain containing 2 [Source:MGI Symbol;Acc:MGI:1 918 988]
Slc1a2	4.34E-35	−0.5523763	0.967	0.998	1.40E-30	solute carrier family 1 (glial high affinity glutamate transporter), member 2 [Source:MGI Symbol;Acc:MGI:101 931]
Nxn	5.99E-35	0.62 688 207	0.651	0.427	1.93E-30	nucleoredoxin [Source:MGI Symbol;Acc:MGI:109 331]
Phactr3	6.21E-35	−0.5799752	0.176	0.516	2.01E-30	phosphatase and actin regulator 3 [Source:MGI Symbol;Acc:MGI:1 921 439]
Ccdc85a	1.62E-34	−0.6265068	0.187	0.519	5.22E-30	coiled-coil domain containing 85A [Source:MGI Symbol;Acc:MGI:2 445 069]
Ndrg2	4.31E-34	−0.4950696	0.579	0.727	1.39E-29	N-myc downstream regulated gene 2 [Source:MGI Symbol;Acc:MGI:1 352 498]
Htra1	5.00E-34	−0.5737498	0.377	0.661	1.61E-29	HtrA serine peptidase 1 [Source:MGI Symbol;Acc:MGI:1 929 076]
St3gal4	8.55E-34	−0.5887351	0.265	0.541	2.76E-29	ST3 beta-galactoside alpha-2,3-sialyltransferase 4 [Source:MGI Symbol;Acc:MGI:1 316 743]
Tmem132b	1.11E-33	0.31 405 038	0.265	0.081	3.59E-29	transmembrane protein 132B [Source:MGI Symbol;Acc:MGI:3 609 245]
Nav3	1.86E-33	−0.6084331	0.317	0.514	6.00E-29	neuron navigator 3 [Source:MGI Symbol;Acc:MGI:2 183 703]
Atp2b4	2.16E-33	0.32 348 148	0.265	0.081	6.97E-29	ATPase, Ca++ transporting, plasma membrane 4 [Source:MGI Symbol;Acc:MGI:88 111]
Met	2.35E-33	0.29 645 257	0.163	0.005	7.60E-29	met proto-oncogene [Source:MGI Symbol;Acc:MGI:96 969]
Flnc	7.50E-33	0.27 142 056	0.141	0.01	2.42E-28	filamin C, gamma [Source:MGI Symbol;Acc:MGI:95 557]
Fat3	1.86E-32	−0.5446937	0.536	0.668	6.01E-28	FAT atypical cadherin 3 [Source:MGI Symbol;Acc:MGI:2 444 314]
Id3	2.99E-32	0.44 213 536	0.382	0.138	9.65E-28	inhibitor of DNA binding 3 [Source:MGI Symbol;Acc:MGI:96 398]
Acsbg1	3.38E-32	−0.5166302	0.375	0.593	1.09E-27	acyl-CoA synthetase bubblegum family member 1 [Source:MGI Symbol;Acc:MGI:2 385 656]
Ptprg	5.17E-32	−0.5485878	0.479	0.631	1.67E-27	NA
Tenm4	6.03E-32	−0.4994723	0.208	0.414	1.95E-27	teneurin transmembrane protein 4 [Source:MGI Symbol;Acc:MGI:2 447 063]
Slc6a1	6.17E-32	−0.4625577	0.39	0.595	1.99E-27	solute carrier family 6 (neurotransmitter transporter, GABA), member 1 [Source:MGI Symbol;Acc:MGI:95 627]
Fgfr3	7.78E-32	−0.4636898	0.416	0.625	2.51E-27	fibroblast growth factor receptor 3 [Source:MGI Symbol;Acc:MGI:95 524]
Kcnip3	2.19E-31	−0.5040672	0.132	0.369	7.08E-27	Kv channel interacting protein 3, calsenilin [Source:MGI Symbol;Acc:MGI:1 929 258]
Ntng1	2.53E-31	0.47 422 667	0.247	0.061	8.18E-27	netrin G1 [Source:MGI Symbol;Acc:MGI:1 934 028]
Tmc7	4.94E-31	−0.4970903	0.091	0.336	1.59E-26	transmembrane channel-like gene family 7 [Source:MGI Symbol;Acc:MGI:2 443 317]
Tafa1	5.30E-31	−0.6589328	0.108	0.308	1.71E-26	TAFA chemokine like family member 1 [Source:MGI Symbol;Acc:MGI:2 443 695]
Frmpd4	6.58E-31	0.36 641 115	0.286	0.136	2.12E-26	FERM and PDZ domain containing 4 [Source:MGI Symbol;Acc:MGI:3 042 378]
Cep85l	2.02E-30	−0.4820037	0.334	0.568	6.52E-26	centrosomal protein 85-like [Source:MGI Symbol;Acc:MGI:3 642 684]
Prickle2	2.16E-30	0.63 320 045	0.61	0.369	6.96E-26	prickle planar cell polarity protein 2 [Source:MGI Symbol;Acc:MGI:1 925 144]
C3	3.37E-30	0.26 171 321	0.148	0.012	1.09E-25	complement component 3 [Source:MGI Symbol;Acc:MGI:88 227]
Pdgfd	4.30E-30	0.64 872 886	0.607	0.389	1.39E-25	platelet-derived growth factor, D polypeptide [Source:MGI Symbol;Acc:MGI:1 919 035]
Miat	4.48E-30	0.28 579 965	0.21	0.056	1.45E-25	myocardial infarction associated transcript (non-protein coding) [Source:MGI Symbol;Acc:MGI:2 444 886]
Kalrn	4.56E-30	−0.5572135	0.249	0.556	1.47E-25	kalirin, RhoGEF kinase [Source:MGI Symbol;Acc:MGI:2 685 385]
Klhl29	4.79E-30	0.34 632 908	0.33	0.128	1.54E-25	kelch-like 29 [Source:MGI Symbol;Acc:MGI:2 683 857]
Osbpl3	5.99E-30	0.32 270 941	0.219	0.057	1.93E-25	oxysterol binding protein-like 3 [Source:MGI Symbol;Acc:MGI:1 918 970]
Atp1b2	2.35E-29	−0.4802299	0.421	0.531	7.59E-25	ATPase, Na+/K + transporting, beta 2 polypeptide [Source:MGI Symbol;Acc:MGI:88 109]
Dgkb	2.41E-29	−0.5456615	0.746	0.896	7.79E-25	diacylglycerol kinase, beta [Source:MGI Symbol;Acc:MGI:2 442 474]
2610307P16Rik	2.50E-29	0.44 161 538	0.265	0.065	8.07E-25	RIKEN cDNA 2610307P16 gene [Source:MGI Symbol;Acc:MGI:1 919 768]
Dock5	2.53E-29	−0.4279339	0.098	0.271	8.17E-25	dedicator of cytokinesis 5 [Source:MGI Symbol;Acc:MGI:2 652 871]
Slco1c1	2.73E-29	−0.5834417	0.15	0.453	8.80E-25	solute carrier organic anion transporter family, member 1c1 [Source:MGI Symbol;Acc:MGI:1 889 679]
Grin2c	4.74E-29	−0.5140905	0.104	0.421	1.53E-24	glutamate receptor, ionotropic, NMDA2C (epsilon 3) [Source:MGI Symbol;Acc:MGI:95 822]
Plekha6	4.74E-29	0.32 322 575	0.262	0.08	1.53E-24	pleckstrin homology domain containing, family A member 6 [Source:MGI Symbol;Acc:MGI:2 388 662]
Luzp2	4.80E-29	−1.136353	0.649	0.888	1.55E-24	leucine zipper protein 2 [Source:MGI Symbol;Acc:MGI:1 889 615]
Apbb1ip	8.64E-29	0.319 461	0.195	0.021	2.79E-24	amyloid beta (A4) precursor protein-binding, family B, member 1 interacting protein [Source:MGI Symbol;Acc:MGI:1 861 354]
Tnik	1.81E-28	−0.4528652	0.848	0.919	5.84E-24	TRAF2 and NCK interacting kinase [Source:MGI Symbol;Acc:MGI:1 916 264]
Grk3	1.89E-28	−0.4867982	0.273	0.562	6.11E-24	G protein-coupled receptor kinase 3 [Source:MGI Symbol;Acc:MGI:87 941]
Nwd1	2.12E-28	−0.4913772	0.458	0.695	6.83E-24	NACHT and WD repeat domain containing 1 [Source:MGI Symbol;Acc:MGI:2 442 268]
Shroom3	3.36E-28	−0.4720592	0.312	0.445	1.08E-23	shroom family member 3 [Source:MGI Symbol;Acc:MGI:1 351 655]
Hip1	4.84E-28	−0.4873346	0.221	0.479	1.56E-23	huntingtin interacting protein 1 [Source:MGI Symbol;Acc:MGI:1 099 804]
Armc2	1.06E-27	0.35 458 049	0.26	0.081	3.43E-23	armadillo repeat containing 2 [Source:MGI Symbol;Acc:MGI:1 916 449]
Plscr4	1.14E-27	0.36 422 822	0.315	0.102	3.67E-23	phospholipid scramblase 4 [Source:MGI Symbol;Acc:MGI:2 143 267]
Cep112	3.75E-27	0.31 022 389	0.258	0.08	1.21E-22	centrosomal protein 112 [Source:MGI Symbol;Acc:MGI:1 923 673]
Ahcyl1	4.67E-27	−0.4613004	0.438	0.664	1.51E-22	S-adenosylhomocysteine hydrolase-like 1 [Source:MGI Symbol;Acc:MGI:2 385 184]
Mertk	5.96E-27	−0.6295402	0.577	0.794	1.92E-22	MER proto-oncogene tyrosine kinase [Source:MGI Symbol;Acc:MGI:96 965]
Tulp4	7.54E-27	0.48 002 098	0.59	0.392	2.43E-22	tubby like protein 4 [Source:MGI Symbol;Acc:MGI:1 916 092]
Tspan7	9.19E-27	−0.6025166	0.557	0.783	2.97E-22	tetraspanin 7 [Source:MGI Symbol;Acc:MGI:1 298 407]
Xylt1	1.90E-26	−0.5173475	0.223	0.501	6.13E-22	xylosyltransferase 1 [Source:MGI Symbol;Acc:MGI:2 451 073]
St3gal6	2.80E-26	0.26 891 079	0.189	0.042	9.05E-22	ST3 beta-galactoside alpha-2,3-sialyltransferase 6 [Source:MGI Symbol;Acc:MGI:1 888 707]
Myo16	3.60E-26	0.3 868 627	0.275	0.094	1.16E-21	myosin XVI [Source:MGI Symbol;Acc:MGI:2 685 951]
Ptprj	3.83E-26	−0.5179752	0.241	0.425	1.24E-21	protein tyrosine phosphatase, receptor type, J [Source:MGI Symbol;Acc:MGI:104 574]
Npas2	3.91E-26	−0.4745273	0.349	0.561	1.26E-21	neuronal PAS domain protein 2 [Source:MGI Symbol;Acc:MGI:109 232]
Abcc4	5.32E-26	0.25 504 183	0.228	0.065	1.72E-21	ATP-binding cassette, sub-family C (CFTR/MRP), member 4 [Source:MGI Symbol;Acc:MGI:2 443 111]
Ablim1	6.53E-26	−0.441237	0.26	0.508	2.11E-21	actin-binding LIM protein 1 [Source:MGI Symbol;Acc:MGI:1 194 500]
Pamr1	9.42E-26	0.31 300 271	0.243	0.08	3.04E-21	peptidase domain containing associated with muscle regeneration 1 [Source:MGI Symbol;Acc:MGI:2 445 082]
Pde4b	2.15E-25	−0.6125836	0.395	0.564	6.93E-21	phosphodiesterase 4B, cAMP specific [Source:MGI Symbol;Acc:MGI:99 557]
Rfx2	3.58E-25	0.25 164 904	0.206	0.053	1.16E-20	regulatory factor X, 2 (influences HLA class II expression) [Source:MGI Symbol;Acc:MGI:106 583]
Gjb6	3.65E-25	−0.3992799	0.106	0.341	1.18E-20	gap junction protein, beta 6 [Source:MGI Symbol;Acc:MGI:107 588]
Rapgef4	6.94E-25	0.3 878 806	0.269	0.128	2.24E-20	Rap guanine nucleotide exchange factor (GEF) 4 [Source:MGI Symbol;Acc:MGI:1 917 723]
Rps6ka5	7.23E-25	0.39 342 234	0.419	0.218	2.34E-20	ribosomal protein S6 kinase, polypeptide 5 [Source:MGI Symbol;Acc:MGI:1 920 336]
Slc6a9	8.63E-25	−0.3417066	0.108	0.265	2.78E-20	solute carrier family 6 (neurotransmitter transporter, glycine), member 9 [Source:MGI Symbol;Acc:MGI:95 760]
Gm35188	1.74E-24	−0.5198597	0.393	0.642	5.61E-20	predicted gene, 35 188 [Source:MGI Symbol;Acc:MGI:5 594 347]
Wdr17	1.84E-24	−0.6736996	0.716	0.842	5.93E-20	WD repeat domain 17 [Source:MGI Symbol;Acc:MGI:1 924 662]
Mgat4c	1.93E-24	−0.3874397	0.508	0.552	6.23E-20	MGAT4 family, member C [Source:MGI Symbol;Acc:MGI:1 914 819]
Il6st	1.96E-24	0.30 887 398	0.293	0.114	6.31E-20	interleukin 6 signal transducer [Source:MGI Symbol;Acc:MGI:96 560]
Fry	2.03E-24	−0.3308365	0.601	0.661	6.56E-20	FRY microtubule binding protein [Source:MGI Symbol;Acc:MGI:2 443 895]
Ddr1	2.06E-24	0.34 612 253	0.319	0.143	6.65E-20	discoidin domain receptor family, member 1 [Source:MGI Symbol;Acc:MGI:99 216]
9530026P05Rik	2.30E-24	0.6 724 121	0.451	0.238	7.43E-20	RIKEN cDNA 9530026P05 gene [Source:MGI Symbol;Acc:MGI:1 924 659]
Plcl1	2.96E-24	−0.5461779	0.39	0.602	9.55E-20	phospholipase C-like 1 [Source:MGI Symbol;Acc:MGI:3 036 262]
Thbs4	3.13E-24	0.26 018 099	0.126	0.018	1.01E-19	thrombospondin 4 [Source:MGI Symbol;Acc:MGI:1 101 779]
Wasf3	4.11E-24	−0.4080166	0.323	0.541	1.33E-19	WASP family, member 3 [Source:MGI Symbol;Acc:MGI:2 658 986]
Bmpr1b	4.19E-24	0.51 910 095	0.777	0.671	1.35E-19	bone morphogenetic protein receptor, type 1B [Source:MGI Symbol;Acc:MGI:107 191]
Abcg2	4.64E-24	0.2 696 169	0.18	0.039	1.50E-19	ATP binding cassette subfamily G member 2 (Junior blood group) [Source:MGI Symbol;Acc:MGI:1 347 061]
Pacrg	5.76E-24	0.51 573 197	0.54	0.305	1.86E-19	PARK2 co-regulated [Source:MGI Symbol;Acc:MGI:1 916 560]
Nav2	6.65E-24	0.50 495 932	0.672	0.526	2.15E-19	neuron navigator 2 [Source:MGI Symbol;Acc:MGI:2 183 691]
Adcy8	6.82E-24	0.49 769 687	0.421	0.238	2.20E-19	adenylate cyclase 8 [Source:MGI Symbol;Acc:MGI:1 341 110]
Arhgef28	7.54E-24	−0.3417158	0.336	0.439	2.43E-19	Rho guanine nucleotide exchange factor (GEF) 28 [Source:MGI Symbol;Acc:MGI:1 346 016]
Phka1	8.43E-24	−0.4988937	0.471	0.645	2.72E-19	phosphorylase kinase alpha 1 [Source:MGI Symbol;Acc:MGI:97 576]
Sorcs2	1.49E-23	−0.4046082	0.102	0.342	4.82E-19	sortilin-related VPS10 domain containing receptor 2 [Source:MGI Symbol;Acc:MGI:1 932 289]
Fgfr2	2.62E-23	−0.4928716	0.696	0.826	8.45E-19	fibroblast growth factor receptor 2 [Source:MGI Symbol;Acc:MGI:95 523]
Vegfa	2.66E-23	−0.4443213	0.323	0.533	8.58E-19	vascular endothelial growth factor A [Source:MGI Symbol;Acc:MGI:103 178]
Phlpp1	2.95E-23	−0.441501	0.67	0.805	9.52E-19	PH domain and leucine rich repeat protein phosphatase 1 [Source:MGI Symbol;Acc:MGI:2 138 327]
Agbl4	3.41E-23	0.31 829 136	0.28	0.156	1.10E-18	ATP/GTP binding protein-like 4 [Source:MGI Symbol;Acc:MGI:1 918 244]
Chd9	3.90E-23	−0.3962457	0.631	0.768	1.26E-18	chromodomain helicase DNA binding protein 9 [Source:MGI Symbol;Acc:MGI:1 924 001]
Syt7	5.17E-23	0.29 056 634	0.256	0.109	1.67E-18	synaptotagmin VII [Source:MGI Symbol;Acc:MGI:1 859 545]
Pgm5	5.43E-23	0.36 064 808	0.208	0.037	1.75E-18	phosphoglucomutase 5 [Source:MGI Symbol;Acc:MGI:1 925 668]
Nrcam	7.62E-23	−0.4277169	0.655	0.822	2.46E-18	neuronal cell adhesion molecule [Source:MGI Symbol;Acc:MGI:104 750]
Esrrg	1.51E-22	−0.3788191	0.358	0.481	4.87E-18	estrogen-related receptor gamma [Source:MGI Symbol;Acc:MGI:1 347 056]
Ski	2.23E-22	0.36 561 522	0.356	0.176	7.19E-18	ski sarcoma viral oncogene homolog (avian) [Source:MGI Symbol;Acc:MGI:98 310]
Lrrc9	3.59E-22	0.25 188 778	0.23	0.074	1.16E-17	leucine rich repeat containing 9 [Source:MGI Symbol;Acc:MGI:1 925 507]
Kmt2c	4.36E-22	−0.4084218	0.503	0.646	1.41E-17	lysine (K)-specific methyltransferase 2C [Source:MGI Symbol;Acc:MGI:2 444 959]
Cdk17	5.13E-22	0.31 776 545	0.364	0.195	1.66E-17	cyclin-dependent kinase 17 [Source:MGI Symbol;Acc:MGI:97 517]
Bmp6	5.80E-22	0.30 526 888	0.189	0.045	1.87E-17	bone morphogenetic protein 6 [Source:MGI Symbol;Acc:MGI:88 182]
Mical2	6.40E-22	−0.4058543	0.453	0.638	2.07E-17	microtubule associated monooxygenase, calponin and LIM domain containing 2 [Source:MGI Symbol;Acc:MGI:2 444 947]
Shc3	6.90E-22	−0.3979053	0.132	0.374	2.23E-17	src homology 2 domain-containing transforming protein C3 [Source:MGI Symbol;Acc:MGI:106 179]
Nim1k	9.23E-22	−0.4065511	0.189	0.406	2.98E-17	NIM1 serine/threonine protein kinase [Source:MGI Symbol;Acc:MGI:2 442 399]
Egfr	1.06E-21	−0.4412839	0.178	0.453	3.42E-17	epidermal growth factor receptor [Source:MGI Symbol;Acc:MGI:95 294]
Ccdc136	1.11E-21	0.34 468 636	0.345	0.188	3.59E-17	coiled-coil domain containing 136 [Source:MGI Symbol;Acc:MGI:1 918 128]
9630028H03Rik	1.23E-21	−0.3985463	0.076	0.296	3.96E-17	RIKEN cDNA 9630028H03 gene [Source:MGI Symbol;Acc:MGI:2 444 526]
Arhgap6	1.37E-21	0.2 759 559	0.167	0.04	4.43E-17	Rho GTPase activating protein 6 [Source:MGI Symbol;Acc:MGI:1 196 332]
Slc25a21	1.67E-21	0.43 983 065	0.367	0.136	5.39E-17	solute carrier family 25 (mitochondrial oxodicarboxylate carrier), member 21 [Source:MGI Symbol;Acc:MGI:2 445 059]
Pde10a	1.67E-21	−0.8799821	0.182	0.397	5.39E-17	phosphodiesterase 10A [Source:MGI Symbol;Acc:MGI:1 345 143]
Igfbp5	2.16E-21	0.35 607 157	0.284	0.109	6.98E-17	insulin-like growth factor binding protein 5 [Source:MGI Symbol;Acc:MGI:96 440]
Dmd	2.40E-21	−0.4948086	0.668	0.818	7.75E-17	dystrophin, muscular dystrophy [Source:MGI Symbol;Acc:MGI:94 909]
Wwox	2.73E-21	0.661 116	0.731	0.531	8.82E-17	WW domain-containing oxidoreductase [Source:MGI Symbol;Acc:MGI:1 931 237]
Flvcr1	2.88E-21	0.28 350 783	0.18	0.058	9.29E-17	feline leukemia virus subgroup C cellular receptor 1 [Source:MGI Symbol;Acc:MGI:2 444 881]
Utrn	3.40E-21	0.56 232 044	0.696	0.592	1.10E-16	utrophin [Source:MGI Symbol;Acc:MGI:104 631]
Etv4	3.67E-21	−0.4148061	0.026	0.27	1.18E-16	ets variant 4 [Source:MGI Symbol;Acc:MGI:99 423]
Gabbr2	4.65E-21	−0.4809581	0.115	0.32	1.50E-16	gamma-aminobutyric acid (GABA) B receptor, 2 [Source:MGI Symbol;Acc:MGI:2 386 030]
Tsc22d1	7.13E-21	0.30 642 531	0.445	0.27	2.30E-16	TSC22 domain family, member 1 [Source:MGI Symbol;Acc:MGI:109 127]
Mgat5	7.30E-21	0.37 459 791	0.514	0.336	2.36E-16	mannoside acetylglucosaminyltransferase 5 [Source:MGI Symbol;Acc:MGI:894 701]
Etnppl	8.27E-21	−0.435439	0.184	0.398	2.67E-16	ethanolamine phosphate phospholyase [Source:MGI Symbol;Acc:MGI:1 919 010]
Kcnj10	1.83E-20	−0.3445052	0.215	0.351	5.91E-16	potassium inwardly-rectifying channel, subfamily J, member 10 [Source:MGI Symbol;Acc:MGI:1 194 504]
Pbx1	2.42E-20	0.45 860 109	0.889	0.901	7.82E-16	pre B cell leukemia homeobox 1 [Source:MGI Symbol;Acc:MGI:97 495]
Kif21a	2.63E-20	0.37 637 538	0.592	0.415	8.50E-16	kinesin family member 21A [Source:MGI Symbol;Acc:MGI:109 188]
F3	2.75E-20	−0.3861518	0.395	0.536	8.88E-16	coagulation factor III [Source:MGI Symbol;Acc:MGI:88 381]
Itpr2	4.32E-20	−0.3844484	0.302	0.484	1.40E-15	inositol 1,4,5-triphosphate receptor 2 [Source:MGI Symbol;Acc:MGI:99 418]
Map2k6	4.93E-20	−0.3807338	0.161	0.373	1.59E-15	mitogen-activated protein kinase kinase 6 [Source:MGI Symbol;Acc:MGI:1 346 870]
Ghr	5.27E-20	−0.4138501	0.293	0.486	1.70E-15	growth hormone receptor [Source:MGI Symbol;Acc:MGI:95 708]
Camk1d	6.31E-20	−0.4628045	0.453	0.671	2.04E-15	calcium/calmodulin-dependent protein kinase ID [Source:MGI Symbol;Acc:MGI:2 442 190]
Tenm3	6.32E-20	0.45 681 296	0.709	0.529	2.04E-15	teneurin transmembrane protein 3 [Source:MGI Symbol;Acc:MGI:1 345 183]
Serpine2	6.56E-20	−0.3778713	0.312	0.489	2.12E-15	serine (or cysteine) peptidase inhibitor, clade E, member 2 [Source:MGI Symbol;Acc:MGI:101 780]
Mpdz	8.64E-20	0.32 366 902	0.325	0.164	2.79E-15	multiple PDZ domain crumbs cell polarity complex component [Source:MGI Symbol;Acc:MGI:1 343 489]
Prr16	8.74E-20	−0.3898367	0.13	0.303	2.82E-15	proline rich 16 [Source:MGI Symbol;Acc:MGI:1 918 623]
Sipa1l1	9.14E-20	0.31 642 906	0.354	0.195	2.95E-15	signal-induced proliferation-associated 1 like 1 [Source:MGI Symbol;Acc:MGI:2 443 679]
9630014M24Rik	1.01E-19	−0.3604998	0.048	0.268	3.25E-15	RIKEN cDNA 9630014M24 gene [Source:MGI Symbol;Acc:MGI:3 588 234]
Pcdh11x	1.06E-19	−0.4301462	0.206	0.418	3.43E-15	protocadherin 11 X-linked [Source:MGI Symbol;Acc:MGI:2 442 849]
Daam2	1.24E-19	−0.4029957	0.38	0.579	4.01E-15	dishevelled associated activator of morphogenesis 2 [Source:MGI Symbol;Acc:MGI:1 923 691]
Myo5a	2.01E-19	0.31 211 767	0.351	0.218	6.49E-15	myosin VA [Source:MGI Symbol;Acc:MGI:105 976]
Ctnnd2	2.21E-19	−0.3354424	0.959	0.979	7.13E-15	catenin (cadherin associated protein), delta 2 [Source:MGI Symbol;Acc:MGI:1 195 966]
Nkain2	2.91E-19	1.07 747 926	0.694	0.465	9.39E-15	Na+/K + transporting ATPase interacting 2 [Source:MGI Symbol;Acc:MGI:1 923 447]
Tprkb	5.34E-19	0.41 617 893	0.54	0.367	1.72E-14	Tp53rk binding protein [Source:MGI Symbol;Acc:MGI:1 917 036]
St6galnac5	5.81E-19	−0.4632976	0.05	0.251	1.87E-14	ST6 (alpha-N-acetyl-neuraminyl-2,3-beta-galactosyl-1,3)-N-acetylgalactosaminide alpha-2,6-sialyltransferase 5 [Source:MGI Symbol;Acc:MGI:1 349 471]
Wnk2	6.61E-19	0.32 581 363	0.56	0.45	2.14E-14	WNK lysine deficient protein kinase 2 [Source:MGI Symbol;Acc:MGI:1 922 857]
Hipk2	1.04E-18	−0.3633073	0.33	0.52	3.34E-14	homeodomain interacting protein kinase 2 [Source:MGI Symbol;Acc:MGI:1 314 872]
Paqr8	1.23E-18	−0.409955	0.534	0.68	3.96E-14	progestin and adipoQ receptor family member VIII [Source:MGI Symbol;Acc:MGI:1 921 479]
Csmd3	1.59E-18	−0.3824358	0.317	0.442	5.14E-14	CUB and Sushi multiple domains 3 [Source:MGI Symbol;Acc:MGI:2 386 403]
Tril	1.66E-18	−0.2956732	0.113	0.258	5.36E-14	TLR4 interactor with leucine-rich repeats [Source:MGI Symbol;Acc:MGI:1 914 123]
Ttc3	1.97E-18	0.33 617 486	0.72	0.614	6.34E-14	tetratricopeptide repeat domain 3 [Source:MGI Symbol;Acc:MGI:1 276 539]
Syn2	2.93E-18	−0.3646474	0.377	0.511	9.45E-14	synapsin II [Source:MGI Symbol;Acc:MGI:103 020]
Zfp462	3.48E-18	0.41 639 775	0.531	0.321	1.12E-13	zinc finger protein 462 [Source:MGI Symbol;Acc:MGI:107 690]
Etv5	4.64E-18	−0.3136522	0.098	0.287	1.50E-13	ets variant 5 [Source:MGI Symbol;Acc:MGI:1 096 867]
Hif1a	4.84E-18	−0.285513	0.124	0.264	1.56E-13	hypoxia inducible factor 1, alpha subunit [Source:MGI Symbol;Acc:MGI:106 918]
Qk	5.84E-18	−0.3095092	0.905	0.952	1.88E-13	NA
Angpt1	6.03E-18	0.36 156 716	0.343	0.153	1.95E-13	angiopoietin 1 [Source:MGI Symbol;Acc:MGI:108 448]
Ptprz1	6.17E-18	−0.407751	0.813	0.897	1.99E-13	protein tyrosine phosphatase, receptor type Z, polypeptide 1 [Source:MGI Symbol;Acc:MGI:97 816]
Pde8b	7.34E-18	−0.4795091	0.193	0.429	2.37E-13	phosphodiesterase 8B [Source:MGI Symbol;Acc:MGI:2 443 999]
Paqr6	8.29E-18	−0.2573612	0.076	0.206	2.68E-13	progestin and adipoQ receptor family member VI [Source:MGI Symbol;Acc:MGI:1 916 207]
Rgs7bp	9.33E-18	−0.3628887	0.154	0.342	3.01E-13	regulator of G-protein signalling 7 binding protein [Source:MGI Symbol;Acc:MGI:106 334]
Gm30382	1.03E-17	−0.3200186	0.037	0.161	3.32E-13	predicted gene, 30 382 [Source:MGI Symbol;Acc:MGI:5 589 541]
Nr3c2	1.42E-17	−0.3763949	0.466	0.632	4.59E-13	nuclear receptor subfamily 3, group C, member 2 [Source:MGI Symbol;Acc:MGI:99 459]
Gm29514	1.61E-17	−0.3119071	0.046	0.218	5.19E-13	predicted gene 29 514 [Source:MGI Symbol;Acc:MGI:5 580 220]
Cdh20	2.29E-17	−0.3272213	0.623	0.715	7.38E-13	cadherin 20 [Source:MGI Symbol;Acc:MGI:1 346 069]
4930545L23Rik	2.29E-17	−0.3123195	0.039	0.207	7.39E-13	RIKEN cDNA 4930545L23 gene [Source:MGI Symbol;Acc:MGI:1 926 055]
Tsc22d3	2.66E-17	−0.3531744	0.167	0.308	8.58E-13	TSC22 domain family, member 3 [Source:MGI Symbol;Acc:MGI:1 196 284]
Mir124-2hg	2.83E-17	−0.3072933	0.111	0.287	9.14E-13	Mir124-2 host gene (non-protein coding) [Source:MGI Symbol;Acc:MGI:1 917 691]
Unc5c	3.79E-17	0.51 316 448	0.345	0.217	1.22E-12	unc-5 netrin receptor C [Source:MGI Symbol;Acc:MGI:1 095 412]
Ralgps2	3.84E-17	0.30 587 309	0.347	0.176	1.24E-12	Ral GEF with PH domain and SH3 binding motif 2 [Source:MGI Symbol;Acc:MGI:1 925 505]
Naaladl2	3.86E-17	0.47 242 159	0.527	0.334	1.25E-12	N-acetylated alpha-linked acidic dipeptidase-like 2 [Source:MGI Symbol;Acc:MGI:2 685 867]
Spred1	3.92E-17	−0.3515126	0.254	0.461	1.27E-12	sprouty protein with EVH-1 domain 1, related sequence [Source:MGI Symbol;Acc:MGI:2 150 016]
Prkce	4.64E-17	0.32 778 049	0.538	0.357	1.50E-12	protein kinase C, epsilon [Source:MGI Symbol;Acc:MGI:97 599]
Erbb4	5.00E-17	−0.2735387	0.564	0.56	1.61E-12	erb-b2 receptor tyrosine kinase 4 [Source:MGI Symbol;Acc:MGI:104 771]
Arhgap26	5.43E-17	−0.374634	0.354	0.529	1.75E-12	Rho GTPase activating protein 26 [Source:MGI Symbol;Acc:MGI:1 918 552]
Tgfb2	6.00E-17	0.27 281 362	0.252	0.101	1.94E-12	transforming growth factor, beta 2 [Source:MGI Symbol;Acc:MGI:98 726]
Rfx4	6.00E-17	0.50 589 577	0.668	0.524	1.94E-12	regulatory factor X, 4 (influences HLA class II expression) [Source:MGI Symbol;Acc:MGI:1 918 387]
Actr3b	6.84E-17	−0.3092165	0.095	0.279	2.21E-12	ARP3 actin-related protein 3B [Source:MGI Symbol;Acc:MGI:2 661 120]
Cacna1c	6.97E-17	0.30 918 393	0.234	0.113	2.25E-12	calcium channel, voltage-dependent, L type, alpha 1C subunit [Source:MGI Symbol;Acc:MGI:103 013]
Nrg2	7.41E-17	0.40 615 154	0.425	0.227	2.39E-12	neuregulin 2 [Source:MGI Symbol;Acc:MGI:1 098 246]
Abcc12	8.05E-17	0.27 124 368	0.124	0.001	2.60E-12	ATP-binding cassette, sub-family C (CFTR/MRP), member 12 [Source:MGI Symbol;Acc:MGI:2 441 679]
Lhfp	1.32E-16	−0.4484381	0.421	0.558	4.25E-12	lipoma HMGIC fusion partner [Source:MGI Symbol;Acc:MGI:1 920 048]
Cdh19	1.37E-16	−0.3995483	0.416	0.639	4.42E-12	cadherin 19, type 2 [Source:MGI Symbol;Acc:MGI:3 588 198]
Efr3b	2.37E-16	−0.3589656	0.252	0.436	7.66E-12	EFR3 homolog B [Source:MGI Symbol;Acc:MGI:2 444 851]
Snrk	2.59E-16	−0.2763449	0.13	0.276	8.35E-12	SNF related kinase [Source:MGI Symbol;Acc:MGI:108 104]
Nrbp2	2.67E-16	0.31 083 032	0.364	0.198	8.61E-12	nuclear receptor binding protein 2 [Source:MGI Symbol;Acc:MGI:2 385 017]
Znrf3	2.68E-16	−0.3601518	0.349	0.541	8.67E-12	zinc and ring finger 3 [Source:MGI Symbol;Acc:MGI:3 039 616]
Slc25a18	2.73E-16	−0.29355	0.182	0.331	8.81E-12	solute carrier family 25 (mitochondrial carrier), member 18 [Source:MGI Symbol;Acc:MGI:1 919 053]
Frem1	2.81E-16	−0.2688484	0.041	0.183	9.07E-12	Fras1 related extracellular matrix protein 1 [Source:MGI Symbol;Acc:MGI:2 670 972]
Irak2	3.22E-16	−0.3594776	0.284	0.462	1.04E-11	interleukin-1 receptor-associated kinase 2 [Source:MGI Symbol;Acc:MGI:2 429 603]
Nnat	3.46E-16	0.49 109 364	0.249	0.168	1.12E-11	neuronatin [Source:MGI Symbol;Acc:MGI:104 716]
Robo1	4.72E-16	−0.3444626	0.304	0.412	1.52E-11	roundabout guidance receptor 1 [Source:MGI Symbol;Acc:MGI:1 274 781]
Macrod1	5.02E-16	−0.2726389	0.124	0.274	1.62E-11	mono-ADP ribosylhydrolase 1 [Source:MGI Symbol;Acc:MGI:2 147 583]
Tafa5	6.98E-16	0.30 683 679	0.395	0.236	2.25E-11	TAFA chemokine like family member 5 [Source:MGI Symbol;Acc:MGI:2 146 182]
Hif3a	8.85E-16	−0.3066472	0.228	0.399	2.86E-11	hypoxia inducible factor 3, alpha subunit [Source:MGI Symbol;Acc:MGI:1 859 778]
Per3	1.19E-15	−0.2763239	0.21	0.352	3.83E-11	period circadian clock 3 [Source:MGI Symbol;Acc:MGI:1 277 134]
Gm6145	1.55E-15	−0.3410727	0.484	0.631	5.01E-11	predicted gene 6145 [Source:MGI Symbol;Acc:MGI:3 779 559]
Cdh22	1.77E-15	−0.2502555	0.093	0.193	5.71E-11	cadherin 22 [Source:MGI Symbol;Acc:MGI:1 341 843]
Itgav	2.98E-15	−0.3058459	0.193	0.375	9.61E-11	integrin alpha V [Source:MGI Symbol;Acc:MGI:96 608]
Ripor2	3.01E-15	−0.2624406	0.033	0.177	9.73E-11	RHO family interacting cell polarization regulator 2 [Source:MGI Symbol;Acc:MGI:2 444 879]
Map1b	3.10E-15	0.33 093 872	0.531	0.428	1.00E-10	microtubule-associated protein 1B [Source:MGI Symbol;Acc:MGI:1 306 778]
Stk32a	3.37E-15	0.63 100 544	0.41	0.226	1.09E-10	serine/threonine kinase 32A [Source:MGI Symbol;Acc:MGI:2 442 403]
Slc7a2	3.54E-15	0.38 023 418	0.458	0.309	1.14E-10	solute carrier family 7 (cationic amino acid transporter, y + system), member 2 [Source:MGI Symbol;Acc:MGI:99 828]
Pros1	3.70E-15	0.26 161 479	0.184	0.076	1.19E-10	protein S (alpha) [Source:MGI Symbol;Acc:MGI:1 095 733]
Glul	4.09E-15	−0.3745361	0.393	0.597	1.32E-10	glutamate-ammonia ligase (glutamine synthetase) [Source:MGI Symbol;Acc:MGI:95 739]
Washc2	4.58E-15	−0.2966635	0.2	0.359	1.48E-10	WASH complex subunit 2 [Source:MGI Symbol;Acc:MGI:106 463]
Sat1	4.69E-15	−0.3285342	0.26	0.416	1.52E-10	spermidine/spermine N1-acetyl transferase 1 [Source:MGI Symbol;Acc:MGI:98 233]
Man1a	5.06E-15	0.25 671 697	0.217	0.09	1.63E-10	mannosidase 1, alpha [Source:MGI Symbol;Acc:MGI:104 677]
Cap2	5.07E-15	−0.2923979	0.161	0.333	1.64E-10	CAP, adenylate cyclase-associated protein, 2 (yeast) [Source:MGI Symbol;Acc:MGI:1 914 502]
Pla2g7	5.26E-15	−0.3265165	0.416	0.595	1.70E-10	phospholipase A2, group VII (platelet-activating factor acetylhydrolase, plasma) [Source:MGI Symbol;Acc:MGI:1 351 327]
Cdh2	5.37E-15	−0.3571341	0.462	0.629	1.73E-10	cadherin 2 [Source:MGI Symbol;Acc:MGI:88 355]
Prex2	8.14E-15	−0.4228434	0.705	0.826	2.63E-10	phosphatidylinositol-3,4,5-trisphosphate-dependent Rac exchange factor 2 [Source:MGI Symbol;Acc:MGI:1 923 385]
Tmcc3	9.77E-15	−0.3073346	0.443	0.567	3.16E-10	transmembrane and coiled coil domains 3 [Source:MGI Symbol;Acc:MGI:2 442 900]
Spag9	1.02E-14	−0.3003432	0.586	0.696	3.28E-10	sperm associated antigen 9 [Source:MGI Symbol;Acc:MGI:1 918 084]
Adcy2	1.06E-14	0.29 525 012	0.577	0.487	3.41E-10	adenylate cyclase 2 [Source:MGI Symbol;Acc:MGI:99 676]
Gli3	1.10E-14	−0.3301032	0.542	0.692	3.55E-10	GLI-Kruppel family member GLI3 [Source:MGI Symbol;Acc:MGI:95 729]
Hmgn3	1.10E-14	−0.3043512	0.141	0.324	3.57E-10	high mobility group nucleosomal binding domain 3 [Source:MGI Symbol;Acc:MGI:2 138 069]
Ldlrad4	1.19E-14	−0.3189325	0.213	0.356	3.84E-10	low density lipoprotein receptor class A domain containing 4 [Source:MGI Symbol;Acc:MGI:1 277 150]
Eps15	1.28E-14	−0.2964594	0.462	0.606	4.14E-10	epidermal growth factor receptor pathway substrate 15 [Source:MGI Symbol;Acc:MGI:104 583]
Gli2	1.39E-14	−0.3222033	0.284	0.459	4.47E-10	GLI-Kruppel family member GLI2 [Source:MGI Symbol;Acc:MGI:95 728]
Dock1	1.56E-14	−0.3261815	0.523	0.673	5.04E-10	dedicator of cytokinesis 1 [Source:MGI Symbol;Acc:MGI:2 429 765]
Sox2ot	1.64E-14	−0.3305237	0.434	0.504	5.30E-10	SOX2 overlapping transcript (non-protein coding) [Source:MGI Symbol;Acc:MGI:2 444 112]
Cnn3	1.70E-14	0.28 344 252	0.317	0.194	5.50E-10	calponin 3, acidic [Source:MGI Symbol;Acc:MGI:1 919 244]
Ttyh1	1.92E-14	−0.2926797	0.605	0.718	6.19E-10	tweety family member 1 [Source:MGI Symbol;Acc:MGI:1 889 007]
Gm44257	2.12E-14	−0.2609873	0.033	0.197	6.85E-10	predicted gene, 44 257 [Source:MGI Symbol;Acc:MGI:5 690 649]
Adamts20	2.16E-14	0.29 924 181	0.273	0.148	6.97E-10	a disintegrin-like and metallopeptidase (reprolysin type) with thrombospondin type 1 motif, 20 [Source:MGI Symbol;Acc:MGI:2 660 628]
Dclk2	2.20E-14	0.30 007 378	0.419	0.267	7.11E-10	doublecortin-like kinase 2 [Source:MGI Symbol;Acc:MGI:1 918 012]
Cacna1a	2.21E-14	0.25 941 802	0.319	0.192	7.14E-10	calcium channel, voltage-dependent, P/Q type, alpha 1A subunit [Source:MGI Symbol;Acc:MGI:109 482]
Plekha5	2.37E-14	0.28 678 228	0.356	0.217	7.64E-10	pleckstrin homology domain containing, family A member 5 [Source:MGI Symbol;Acc:MGI:1 923 802]
Kifc3	2.45E-14	−0.2597676	0.161	0.317	7.90E-10	kinesin family member C3 [Source:MGI Symbol;Acc:MGI:109 202]
Lgr6	2.51E-14	−0.259391	0.024	0.156	8.11E-10	leucine-rich repeat-containing G protein-coupled receptor 6 [Source:MGI Symbol;Acc:MGI:2 441 805]
Eda	2.59E-14	0.32 338 302	0.362	0.222	8.37E-10	ectodysplasin-A [Source:MGI Symbol;Acc:MGI:1 195 272]
Ccdc162	2.67E-14	0.31 699 302	0.269	0.124	8.61E-10	coiled-coil domain containing 162 [Source:MGI Symbol;Acc:MGI:1 923 223]
Arid5b	3.24E-14	0.2 963 038	0.282	0.15	1.04E-09	AT rich interactive domain 5B (MRF1-like) [Source:MGI Symbol;Acc:MGI:2 175 912]
Garem1	3.77E-14	−0.3605985	0.254	0.449	1.22E-09	GRB2 associated regulator of MAPK1 subtype 1 [Source:MGI Symbol;Acc:MGI:2 685 790]
Arhgap5	3.93E-14	−0.3348438	0.662	0.757	1.27E-09	Rho GTPase activating protein 5 [Source:MGI Symbol;Acc:MGI:1 332 637]
Immp2l	3.95E-14	0.31 435 518	0.33	0.194	1.27E-09	IMP2 inner mitochondrial membrane peptidase-like (S. cerevisiae) [Source:MGI Symbol;Acc:MGI:2 135 611]
Aldoc	4.99E-14	−0.2564749	0.356	0.476	1.61E-09	aldolase C, fructose-bisphosphate [Source:MGI Symbol;Acc:MGI:101 863]
Grip1	5.24E-14	−0.320981	0.124	0.269	1.69E-09	glutamate receptor interacting protein 1 [Source:MGI Symbol;Acc:MGI:1 921 303]
Fgf1	5.34E-14	−0.2747626	0.197	0.346	1.73E-09	fibroblast growth factor 1 [Source:MGI Symbol;Acc:MGI:95 515]
App	5.83E-14	0.32 161 687	0.655	0.53	1.88E-09	amyloid beta (A4) precursor protein [Source:MGI Symbol;Acc:MGI:88 059]
Wipf1	6.20E-14	−0.2843777	0.139	0.254	2.00E-09	WAS/WASL interacting protein family, member 1 [Source:MGI Symbol;Acc:MGI:2 178 801]
Cyfip1	7.55E-14	−0.2702309	0.219	0.367	2.44E-09	cytoplasmic FMR1 interacting protein 1 [Source:MGI Symbol;Acc:MGI:1 338 801]
Gnao1	8.00E-14	−0.5415402	0.594	0.675	2.58E-09	guanine nucleotide binding protein, alpha O [Source:MGI Symbol;Acc:MGI:95 775]
Ccdc141	8.02E-14	−0.2931722	0.341	0.454	2.59E-09	coiled-coil domain containing 141 [Source:MGI Symbol;Acc:MGI:1 919 735]
Stard13	8.21E-14	0.4 152 649	0.555	0.388	2.65E-09	StAR-related lipid transfer (START) domain containing 13 [Source:MGI Symbol;Acc:MGI:2 385 331]
Fut9	8.62E-14	−0.322833	0.538	0.66	2.78E-09	fucosyltransferase 9 [Source:MGI Symbol;Acc:MGI:1 330 859]
Sycp2	9.81E-14	−0.2689264	0.104	0.267	3.17E-09	synaptonemal complex protein 2 [Source:MGI Symbol;Acc:MGI:1 933 281]
Tox	1.04E-13	−0.3711668	0.299	0.514	3.36E-09	thymocyte selection-associated high mobility group box [Source:MGI Symbol;Acc:MGI:2 181 659]
Eif4g3	1.08E-13	−0.3246149	0.401	0.571	3.48E-09	eukaryotic translation initiation factor 4 gamma, 3 [Source:MGI Symbol;Acc:MGI:1 923 935]
Stat3	1.34E-13	0.27 159 352	0.306	0.153	4.34E-09	signal transducer and activator of transcription 3 [Source:MGI Symbol;Acc:MGI:103 038]
Sned1	1.48E-13	0.2 999 979	0.347	0.2	4.78E-09	sushi, nidogen and EGF-like domains 1 [Source:MGI Symbol;Acc:MGI:3 045 960]
Usp54	1.72E-13	−0.271357	0.206	0.345	5.55E-09	ubiquitin specific peptidase 54 [Source:MGI Symbol;Acc:MGI:1 926 037]
Tle4	2.16E-13	−0.3141217	0.249	0.441	6.97E-09	transducin-like enhancer of split 4 [Source:MGI Symbol;Acc:MGI:104 633]
Dbx2	2.45E-13	−0.2581829	0.221	0.358	7.91E-09	developing brain homeobox 2 [Source:MGI Symbol;Acc:MGI:107 445]
Btbd9	2.62E-13	0.31 317 453	0.508	0.346	8.47E-09	BTB (POZ) domain containing 9 [Source:MGI Symbol;Acc:MGI:1 916 625]
Fam214a	2.90E-13	−0.2777073	0.213	0.354	9.37E-09	NA
Cachd1	3.12E-13	0.33 962 337	0.477	0.327	1.01E-08	cache domain containing 1 [Source:MGI Symbol;Acc:MGI:2 444 177]
Fnbp1	3.31E-13	−0.2930825	0.636	0.734	1.07E-08	formin binding protein 1 [Source:MGI Symbol;Acc:MGI:109 606]
Pid1	3.41E-13	−0.3188128	0.347	0.512	1.10E-08	phosphotyrosine interaction domain containing 1 [Source:MGI Symbol;Acc:MGI:2 138 391]
Id4	3.49E-13	0.36 185 574	0.462	0.282	1.13E-08	inhibitor of DNA binding 4 [Source:MGI Symbol;Acc:MGI:99 414]
Chst11	3.52E-13	0.31 210 577	0.412	0.262	1.14E-08	carbohydrate sulfotransferase 11 [Source:MGI Symbol;Acc:MGI:1 927 166]
Atl2	4.05E-13	−0.2610801	0.304	0.424	1.31E-08	atlastin GTPase 2 [Source:MGI Symbol;Acc:MGI:1 929 492]
Dpf3	5.01E-13	−0.2910054	0.265	0.426	1.62E-08	double PHD fingers 3 [Source:MGI Symbol;Acc:MGI:1 917 377]
Arrb1	5.31E-13	−0.2537328	0.18	0.339	1.71E-08	arrestin, beta 1 [Source:MGI Symbol;Acc:MGI:99 473]
Fam155a	5.80E-13	0.69 052 615	0.484	0.287	1.87E-08	NA
Zfhx4	7.06E-13	0.3 268 659	0.356	0.23	2.28E-08	zinc finger homeodomain 4 [Source:MGI Symbol;Acc:MGI:2 137 668]
Fgf14	7.06E-13	0.39 407 139	0.863	0.758	2.28E-08	fibroblast growth factor 14 [Source:MGI Symbol;Acc:MGI:109 189]
Hivep3	7.55E-13	−0.3266302	0.575	0.729	2.44E-08	human immunodeficiency virus type I enhancer binding protein 3 [Source:MGI Symbol;Acc:MGI:106 589]
Fermt2	8.05E-13	−0.2998407	0.341	0.501	2.60E-08	fermitin family member 2 [Source:MGI Symbol;Acc:MGI:2 385 001]
Rnf220	8.48E-13	−0.2999229	0.102	0.193	2.74E-08	ring finger protein 220 [Source:MGI Symbol;Acc:MGI:1 913 993]
Rmst	1.07E-12	0.52 794 937	0.803	0.758	3.47E-08	rhabdomyosarcoma 2 associated transcript (non-coding RNA) [Source:MGI Symbol;Acc:MGI:1 099 806]
Dnm3	1.48E-12	0.3 694 322	0.49	0.306	4.77E-08	dynamin 3 [Source:MGI Symbol;Acc:MGI:1 341 299]
Gulp1	1.67E-12	−0.2701814	0.15	0.295	5.38E-08	GULP, engulfment adaptor PTB domain containing 1 [Source:MGI Symbol;Acc:MGI:1 920 407]
Prkn	1.96E-12	0.42 415 941	0.735	0.6	6.32E-08	parkin RBR E3 ubiquitin protein ligase [Source:MGI Symbol;Acc:MGI:1 355 296]
Hivep2	2.85E-12	−0.3000869	0.401	0.544	9.21E-08	human immunodeficiency virus type I enhancer binding protein 2 [Source:MGI Symbol;Acc:MGI:1 338 076]
Agl	2.91E-12	−0.2530156	0.282	0.41	9.40E-08	amylo-1,6-glucosidase, 4-alpha-glucanotransferase [Source:MGI Symbol;Acc:MGI:1 924 809]
Astn2	3.41E-12	0.43 229 755	0.59	0.514	1.10E-07	astrotactin 2 [Source:MGI Symbol;Acc:MGI:1 889 277]
Abi1	3.62E-12	−0.2695589	0.334	0.476	1.17E-07	abl interactor 1 [Source:MGI Symbol;Acc:MGI:104 913]
Enah	4.34E-12	−0.2949006	0.557	0.689	1.40E-07	ENAH actin regulator [Source:MGI Symbol;Acc:MGI:108 360]
Tmem164	5.37E-12	−0.3220514	0.356	0.514	1.74E-07	transmembrane protein 164 [Source:MGI Symbol;Acc:MGI:2 148 020]
Aco2	5.41E-12	−0.250002	0.295	0.434	1.75E-07	aconitase 2, mitochondrial [Source:MGI Symbol;Acc:MGI:87 880]
Dlgap1	5.65E-12	−0.3280328	0.725	0.847	1.82E-07	DLG associated protein 1 [Source:MGI Symbol;Acc:MGI:1 346 065]
Cpeb3	5.85E-12	−0.2707785	0.438	0.553	1.89E-07	cytoplasmic polyadenylation element binding protein 3 [Source:MGI Symbol;Acc:MGI:2 443 075]
Nfib	7.15E-12	−0.2799681	0.74	0.821	2.31E-07	nuclear factor I/B [Source:MGI Symbol;Acc:MGI:103 188]
Iqsec2	8.06E-12	0.25 033 821	0.336	0.196	2.60E-07	IQ motif and Sec7 domain 2 [Source:MGI Symbol;Acc:MGI:3 528 396]
Shisa6	8.24E-12	−0.2575845	0.1	0.159	2.66E-07	shisa family member 6 [Source:MGI Symbol;Acc:MGI:2 685 725]
Nfkb1	8.60E-12	0.31 729 213	0.451	0.309	2.78E-07	nuclear factor of kappa light polypeptide gene enhancer in B cells 1, p105 [Source:MGI Symbol;Acc:MGI:97 312]
Myo18a	9.58E-12	−0.2661236	0.232	0.385	3.09E-07	myosin XVIIIA [Source:MGI Symbol;Acc:MGI:2 667 185]
Ugp2	1.08E-11	0.26 792 952	0.516	0.372	3.49E-07	UDP-glucose pyrophosphorylase 2 [Source:MGI Symbol;Acc:MGI:2 183 447]
Enpp2	1.36E-11	0.30 137 984	0.41	0.242	4.40E-07	ectonucleotide pyrophosphatase/phosphodiesterase 2 [Source:MGI Symbol;Acc:MGI:1 321 390]
Frmpd1	1.37E-11	−0.2725015	0.254	0.404	4.42E-07	FERM and PDZ domain containing 1 [Source:MGI Symbol;Acc:MGI:2 446 274]
Ano6	2.02E-11	0.28 037 371	0.269	0.142	6.53E-07	anoctamin 6 [Source:MGI Symbol;Acc:MGI:2 145 890]
Tcf25	2.14E-11	−0.2524353	0.495	0.625	6.92E-07	transcription factor 25 (basic helix-loop-helix) [Source:MGI Symbol;Acc:MGI:1 914 105]
Sel1l3	2.21E-11	0.25 017 174	0.2	0.101	7.13E-07	sel-1 suppressor of lin-12-like 3 (C. elegans) [Source:MGI Symbol;Acc:MGI:1 916 941]
Pigk	2.24E-11	−0.3192257	0.108	0.257	7.24E-07	phosphatidylinositol glycan anchor biosynthesis, class K [Source:MGI Symbol;Acc:MGI:1 913 863]
Meis2	2.39E-11	0.28 700 823	0.722	0.64	7.73E-07	Meis homeobox 2 [Source:MGI Symbol;Acc:MGI:108 564]
Clmn	2.43E-11	−0.3741937	0.338	0.501	7.85E-07	calmin [Source:MGI Symbol;Acc:MGI:2 136 957]
Pard3b	2.59E-11	0.36 304 281	0.677	0.57	8.37E-07	par-3 family cell polarity regulator beta [Source:MGI Symbol;Acc:MGI:1 919 301]
Zfhx3	2.74E-11	0.44 295 793	0.518	0.363	8.84E-07	zinc finger homeobox 3 [Source:MGI Symbol;Acc:MGI:99 948]
Bcan	2.75E-11	−0.2554758	0.557	0.649	8.87E-07	brevican [Source:MGI Symbol;Acc:MGI:1 096 385]
Acss1	3.09E-11	−0.2567365	0.243	0.397	9.98E-07	acyl-CoA synthetase short-chain family member 1 [Source:MGI Symbol;Acc:MGI:1 915 988]
Adk	3.22E-11	−0.2820038	0.657	0.766	1.04E-06	adenosine kinase [Source:MGI Symbol;Acc:MGI:87 930]
Clasp2	4.17E-11	−0.2734401	0.599	0.691	1.35E-06	CLIP associating protein 2 [Source:MGI Symbol;Acc:MGI:1 923 749]
Alcam	5.04E-11	−0.3209116	0.364	0.519	1.63E-06	activated leukocyte cell adhesion molecule [Source:MGI Symbol;Acc:MGI:1 313 266]
Cspp1	5.14E-11	0.26 083 565	0.486	0.348	1.66E-06	centrosome and spindle pole associated protein 1 [Source:MGI Symbol;Acc:MGI:2 681 832]
Plpp3	5.22E-11	−0.317785	0.696	0.788	1.68E-06	phospholipid phosphatase 3 [Source:MGI Symbol;Acc:MGI:1 915 166]
Megf10	5.80E-11	−0.2519749	0.28	0.419	1.87E-06	multiple EGF-like-domains 10 [Source:MGI Symbol;Acc:MGI:2 685 177]
Gphn	6.45E-11	−0.2689496	0.874	0.911	2.08E-06	gephyrin [Source:MGI Symbol;Acc:MGI:109 602]
Fus	6.61E-11	0.25 396 173	0.516	0.376	2.13E-06	fused in sarcoma [Source:MGI Symbol;Acc:MGI:1 353 633]
Wwc1	8.27E-11	−0.2659263	0.412	0.538	2.67E-06	WW, C2 and coiled-coil domain containing 1 [Source:MGI Symbol;Acc:MGI:2 388 637]
Lrrc4c	8.56E-11	−0.4415473	0.894	0.901	2.76E-06	leucine rich repeat containing 4C [Source:MGI Symbol;Acc:MGI:2 442 636]
Rorb	1.46E-10	−0.3277256	0.746	0.857	4.71E-06	RAR-related orphan receptor beta [Source:MGI Symbol;Acc:MGI:1 343 464]
Sgip1	1.72E-10	−0.2827046	0.625	0.726	5.55E-06	SH3-domain GRB2-like (endophilin) interacting protein 1 [Source:MGI Symbol;Acc:MGI:1 920 344]
Gm48742	1.76E-10	−0.2586425	0.23	0.367	5.69E-06	predicted gene, 48 742 [Source:MGI Symbol;Acc:MGI:6 098 410]
Ttc28	2.09E-10	0.28 895 935	0.497	0.373	6.76E-06	tetratricopeptide repeat domain 28 [Source:MGI Symbol;Acc:MGI:2 140 873]
Igsf11	2.14E-10	0.28 984 729	0.492	0.353	6.90E-06	immunoglobulin superfamily, member 11 [Source:MGI Symbol;Acc:MGI:2 388 477]
Fbxl7	2.42E-10	0.30 362 553	0.215	0.082	7.83E-06	F-box and leucine-rich repeat protein 7 [Source:MGI Symbol;Acc:MGI:3 052 506]
Lama2	2.60E-10	−0.3106816	0.616	0.718	8.39E-06	laminin, alpha 2 [Source:MGI Symbol;Acc:MGI:99 912]
Camk2n1	2.74E-10	−0.2732411	0.269	0.459	8.84E-06	calcium/calmodulin-dependent protein kinase II inhibitor 1 [Source:MGI Symbol;Acc:MGI:1 913 509]
Spire1	3.03E-10	−0.2678142	0.551	0.672	9.78E-06	spire type actin nucleation factor 1 [Source:MGI Symbol;Acc:MGI:1 915 416]
Thrb	3.43E-10	−0.2655069	0.616	0.676	1.11E-05	thyroid hormone receptor beta [Source:MGI Symbol;Acc:MGI:98 743]
Grid1	3.76E-10	−0.2721874	0.275	0.445	1.21E-05	glutamate receptor, ionotropic, delta 1 [Source:MGI Symbol;Acc:MGI:95 812]
Dst	3.99E-10	0.33 094 572	0.688	0.635	1.29E-05	dystonin [Source:MGI Symbol;Acc:MGI:104 627]
Lhfpl3	5.06E-10	0.52 784 176	0.438	0.288	1.63E-05	lipoma HMGIC fusion partner-like 3 [Source:MGI Symbol;Acc:MGI:1 925 076]
Lrmda	5.92E-10	−0.3362263	0.219	0.36	1.91E-05	leucine rich melanocyte differentiation associated [Source:MGI Symbol;Acc:MGI:1 923 883]
Slc9a9	1.26E-09	0.27 808 996	0.488	0.373	4.08E-05	solute carrier family 9 (sodium/hydrogen exchanger), member 9 [Source:MGI Symbol;Acc:MGI:2 679 732]
Mapk10	1.51E-09	−0.2638008	0.568	0.662	4.87E-05	mitogen-activated protein kinase 10 [Source:MGI Symbol;Acc:MGI:1 346 863]
B3galt1	2.00E-09	−0.2654669	0.861	0.919	6.45E-05	UDP-Gal:betaGlcNAc beta 1,3-galactosyltransferase, polypeptide 1 [Source:MGI Symbol;Acc:MGI:1 349 403]
Ddah1	2.19E-09	−0.2550976	0.217	0.381	7.07E-05	dimethylarginine dimethylaminohydrolase 1 [Source:MGI Symbol;Acc:MGI:1 916 469]
Smyd3	3.06E-09	0.30 146 981	0.512	0.361	9.89E-05	SET and MYND domain containing 3 [Source:MGI Symbol;Acc:MGI:1 916 976]
Pde4d	3.57E-09	0.49 104 454	0.816	0.741	0.00 011 538	phosphodiesterase 4D, cAMP specific [Source:MGI Symbol;Acc:MGI:99 555]
Prdx6	3.89E-09	0.28 389 077	0.64	0.527	0.00 012 564	peroxiredoxin 6 [Source:MGI Symbol;Acc:MGI:894 320]
Ankrd28	3.94E-09	0.27 308 201	0.479	0.355	0.0 001 273	ankyrin repeat domain 28 [Source:MGI Symbol;Acc:MGI:2 145 661]
Map4k4	4.15E-09	0.25 600 624	0.518	0.417	0.00 013 397	mitogen-activated protein kinase kinase kinase kinase 4 [Source:MGI Symbol;Acc:MGI:1 349 394]
Acss3	4.20E-09	0.26 918 853	0.321	0.189	0.00 013 546	acyl-CoA synthetase short-chain family member 3 [Source:MGI Symbol;Acc:MGI:2 685 720]
Dock7	4.82E-09	0.27 946 924	0.408	0.292	0.00 015 556	dedicator of cytokinesis 7 [Source:MGI Symbol;Acc:MGI:1 914 549]
Bcl2	8.22E-09	0.25 489 851	0.408	0.338	0.00 026 533	B cell leukemia/lymphoma 2 [Source:MGI Symbol;Acc:MGI:88 138]
4930488L21Rik	1.21E-08	−0.3121966	0.143	0.283	0.00 039 211	RIKEN cDNA 4930488L21 gene [Source:MGI Symbol;Acc:MGI:1 923 059]
Fhit	2.18E-08	0.40 458 542	0.553	0.388	0.00 070 321	fragile histidine triad gene [Source:MGI Symbol;Acc:MGI:1 277 947]
Ank	2.18E-08	0.32 658 131	0.512	0.422	0.00 070 377	progressive ankylosis [Source:MGI Symbol;Acc:MGI:3 045 421]
Fam13c	3.14E-08	−0.2522978	0.15	0.278	0.00 101 426	family with sequence similarity 13, member C [Source:MGI Symbol;Acc:MGI:1 918 971]
Thsd7a	3.44E-08	0.31 348 494	0.451	0.303	0.00 111 155	thrombospondin, type I, domain containing 7A [Source:MGI Symbol;Acc:MGI:2 685 683]
Epha5	4.54E-08	0.30 165 598	0.542	0.382	0.00 146 634	Eph receptor A5 [Source:MGI Symbol;Acc:MGI:99 654]
Phactr1	5.31E-08	−0.2799404	0.332	0.498	0.00 171 272	phosphatase and actin regulator 1 [Source:MGI Symbol;Acc:MGI:2 659 021]
Inpp4b	9.18E-08	0.25 348 123	0.265	0.151	0.00 296 391	inositol polyphosphate-4-phosphatase, type II [Source:MGI Symbol;Acc:MGI:2 158 925]

Among the enriched biological terms associated with the upregulated genes are neuron and cell differentiation, regulation of cell migration, cell junction organization, and protein binding. Additionally, enriched biological functions unique to AG2 and not observed in AG1 included astrocyte end-foot, T-tubule, actin filament organization, and regulation of cell migration.

Moreover, we identified 16 TFs (only 1 shared with AG1 population) found upregulated, including Glis3, Stat3, Smad6, Atoh8, Arid5b, Nfkb1 and Id3.

Complement component 3 (C3) was exclusively expressed in AG2 of spinal cord. GFAP/C3-positive astrocytes subpopulations have been reported in numerous brain samples from neurodegenerative disease patients such as Alzheimer's disease, multiple sclerosis, Huntington's disease, Parkinson's disease, and ALS [67, 95, 96].

The expression of Gfap and other accompanying markers links AG2 with the Gfap positive astrocyte population reported in previous studies and described as an activated-state glial population in normal physiological conditions, and reactive astrocytes in disease [64, 67, 97, 98, 80].

### Identification of a large astrocytic population related with mature astrocyte functions

AG 3, by comparison, was the largest and most heterogeneous. Each AG3 was comprised on average by three clusters on each sample indicating intra-specimen heterogeneity, with a high similarity score and bound tightly together in the similarity graph (Fig. 3B). We found 158 genes significantly overexpressed (*P*-value < 0.005) in contrast to AG1 and AG2 (Table 3). Biological term enrichment and ontology analysis of upregulated genes uncover functions such as post synapse, neuron projection, synaptic membrane, glutamatergic synapse, neuron to neuron synapse, postsynaptic specialization, and postsynaptic density.

**Table 3.** Differentially upregulated genes (*P-*value < 0.005) in AG 3 in comparison with AG1 and AG2

Gene	p_val	avg_log2FC	pct.1	pct.2	p_val_adj	description
St6galnac3	1.53E-241	−1.5936279	0.099	0.537	4.95E-237	ST6 (alpha-N-acetyl-neuraminyl-2,3-beta-galactosyl-1,3)-N-acetylgalactosaminide alpha-2,6-sialyltransferase 3 [Source:MGI Symbol;Acc:MGI:1 341 828]
Ablim2	1.04E-231	−1.6423225	0.132	0.472	3.36E-227	actin-binding LIM protein 2 [Source:MGI Symbol;Acc:MGI:2 385 758]
Nkain2	1.31E-205	−1.7842838	0.436	0.711	4.24E-201	Na+/K + transporting ATPase interacting 2 [Source:MGI Symbol;Acc:MGI:1 923 447]
Gpc5	3.70E-156	1.4 386 111	0.985	0.655	1.19E-151	glypican 5 [Source:MGI Symbol;Acc:MGI:1 194 894]
Lsamp	2.79E-149	1.03 552 766	1	0.996	9.01E-145	limbic system-associated membrane protein [Source:MGI Symbol;Acc:MGI:1 261 760]
Pkp4	8.39E-146	−1.003656	0.215	0.54	2.71E-141	plakophilin 4 [Source:MGI Symbol;Acc:MGI:109 281]
Gfap	4.94E-145	−1.4446486	0.208	0.536	1.60E-140	glial fibrillary acidic protein [Source:MGI Symbol;Acc:MGI:95 697]
Prune2	4.16E-140	−1.0552678	0.127	0.476	1.34E-135	prune homolog 2 [Source:MGI Symbol;Acc:MGI:1 925 004]
Grik2	2.62E-136	−1.1644838	0.444	0.745	8.45E-132	glutamate receptor, ionotropic, kainate 2 (beta 2) [Source:MGI Symbol;Acc:MGI:95 815]
Prr5l	2.29E-127	−1.0058918	0.05	0.352	7.38E-123	proline rich 5 like [Source:MGI Symbol;Acc:MGI:1 919 696]
Slc8a1	1.72E-121	−1.0143689	0.218	0.523	5.56E-117	solute carrier family 8 (sodium/calcium exchanger), member 1 [Source:MGI Symbol;Acc:MGI:107 956]
Trpm3	1.51E-117	1.51 328 014	0.952	0.563	4.88E-113	transient receptor potential cation channel, subfamily M, member 3 [Source:MGI Symbol;Acc:MGI:2 443 101]
Sorbs2	2.64E-116	−1.1643688	0.172	0.467	8.53E-112	sorbin and SH3 domain containing 2 [Source:MGI Symbol;Acc:MGI:1 924 574]
Gm3764	1.75E-112	0.96 079 984	0.952	0.749	5.65E-108	predicted gene 3764 [Source:MGI Symbol;Acc:MGI:3 781 938]
Adamtsl1	4.27E-109	−0.8447468	0.02	0.316	1.38E-104	ADAMTS-like 1 [Source:MGI Symbol;Acc:MGI:1 924 989]
Cacnb2	4.88E-109	−1.1412021	0.251	0.528	1.58E-104	calcium channel, voltage-dependent, beta 2 subunit [Source:MGI Symbol;Acc:MGI:894 644]
Kirrel3	3.05E-108	1.28 033 172	0.89	0.528	9.85E-104	kirre like nephrin family adhesion molecule 3 [Source:MGI Symbol;Acc:MGI:1 914 953]
Mdga2	8.42E-95	0.93 802 895	0.979	0.85	2.72E-90	MAM domain containing glycosylphosphatidylinositol anchor 2 [Source:MGI Symbol;Acc:MGI:2 444 706]
Cobll1	4.35E-94	−0.5949885	0.044	0.318	1.41E-89	Cobl-like 1 [Source:MGI Symbol;Acc:MGI:2 442 894]
Slc38a1	1.99E-92	−0.8966144	0.232	0.507	6.42E-88	solute carrier family 38, member 1 [Source:MGI Symbol;Acc:MGI:2 145 895]
Ccdc148	4.14E-92	−0.7860848	0.053	0.291	1.34E-87	coiled-coil domain containing 148 [Source:MGI Symbol;Acc:MGI:3 039 583]
Nrxn1	4.43E-92	0.87 728 253	0.998	0.91	1.43E-87	neurexin I [Source:MGI Symbol;Acc:MGI:1 096 391]
Plcb1	1.88E-91	1.13 264 861	0.951	0.748	6.07E-87	phospholipase C, beta 1 [Source:MGI Symbol;Acc:MGI:97 613]
A2m	4.93E-90	−0.8817557	0.065	0.298	1.59E-85	alpha-2-macroglobulin [Source:MGI Symbol;Acc:MGI:2 449 119]
Tmeff2	6.26E-89	−0.8573165	0.158	0.353	2.02E-84	transmembrane protein with EGF-like and two follistatin-like domains 2 [Source:MGI Symbol;Acc:MGI:1 861 735]
Dgki	8.89E-87	−0.7768695	0.221	0.536	2.87E-82	diacylglycerol kinase, iota [Source:MGI Symbol;Acc:MGI:2 443 430]
Kcnd2	1.16E-84	1.29 103 548	0.72	0.44	3.74E-80	potassium voltage-gated channel, Shal-related family, member 2 [Source:MGI Symbol;Acc:MGI:102 663]
Pdzrn3	1.83E-84	−0.613695	0.084	0.372	5.89E-80	PDZ domain containing RING finger 3 [Source:MGI Symbol;Acc:MGI:1 933 157]
Slc24a2	2.21E-83	−0.8937233	0.315	0.427	7.15E-79	solute carrier family 24 (sodium/potassium/calcium exchanger), member 2 [Source:MGI Symbol;Acc:MGI:1 923 626]
Gria2	5.83E-83	0.91 744 617	0.775	0.493	1.88E-78	glutamate receptor, ionotropic, AMPA2 (alpha 2) [Source:MGI Symbol;Acc:MGI:95 809]
Grm7	1.28E-82	−0.8519308	0.212	0.436	4.12E-78	glutamate receptor, metabotropic 7 [Source:MGI Symbol;Acc:MGI:1 351 344]
Gm20713	2.19E-81	0.85 012 078	0.727	0.283	7.08E-77	predicted gene 20 713 [Source:MGI Symbol;Acc:MGI:5 313 160]
Cadps	1.18E-80	−0.8169065	0.299	0.571	3.79E-76	Ca2+-dependent secretion activator [Source:MGI Symbol;Acc:MGI:1 350 922]
Rora	2.23E-78	0.80 516 094	0.991	0.959	7.20E-74	RAR-related orphan receptor alpha [Source:MGI Symbol;Acc:MGI:104 661]
Kcnj3	1.93E-77	−0.6938563	0.158	0.453	6.24E-73	potassium inwardly-rectifying channel, subfamily J, member 3 [Source:MGI Symbol;Acc:MGI:104 742]
Slc6a11	2.45E-77	0.82 740 909	0.597	0.302	7.92E-73	solute carrier family 6 (neurotransmitter transporter, GABA), member 11 [Source:MGI Symbol;Acc:MGI:95 630]
Gm14964	5.63E-76	−0.5812385	0.154	0.403	1.82E-71	predicted gene 14 964 [Source:MGI Symbol;Acc:MGI:3 641 621]
Slc7a10	8.30E-75	0.80 503 351	0.57	0.172	2.68E-70	solute carrier family 7 (cationic amino acid transporter, y + system), member 10 [Source:MGI Symbol;Acc:MGI:1 858 261]
Ccdc3	1.94E-73	−0.5646798	0.02	0.244	6.25E-69	coiled-coil domain containing 3 [Source:MGI Symbol;Acc:MGI:1 921 436]
Prickle2	8.03E-73	−0.709148	0.345	0.607	2.59E-68	prickle planar cell polarity protein 2 [Source:MGI Symbol;Acc:MGI:1 925 144]
Brinp3	1.40E-70	0.82 937 771	0.716	0.353	4.52E-66	bone morphogenetic protein/retinoic acid inducible neural specific 3 [Source:MGI Symbol;Acc:MGI:2 443 035]
Pex5l	1.61E-70	−0.8327684	0.183	0.329	5.20E-66	peroxisomal biogenesis factor 5-like [Source:MGI Symbol;Acc:MGI:1 916 672]
Prkca	2.52E-70	−0.7776889	0.551	0.752	8.12E-66	protein kinase C, alpha [Source:MGI Symbol;Acc:MGI:97 595]
Asap3	7.08E-70	−0.4526105	0.025	0.239	2.29E-65	ArfGAP with SH3 domain, ankyrin repeat and PH domain 3 [Source:MGI Symbol;Acc:MGI:2 684 986]
St18	8.32E-70	−0.7018588	0.063	0.225	2.69E-65	suppression of tumorigenicity 18 [Source:MGI Symbol;Acc:MGI:2 446 700]
Ldb2	1.58E-69	−0.5796662	0.114	0.359	5.09E-65	LIM domain binding 2 [Source:MGI Symbol;Acc:MGI:894 670]
Edil3	2.54E-69	−0.68913	0.178	0.362	8.21E-65	EGF-like repeats and discoidin I-like domains 3 [Source:MGI Symbol;Acc:MGI:1 329 025]
Unc5c	5.97E-68	−0.7357904	0.182	0.42	1.93E-63	unc-5 netrin receptor C [Source:MGI Symbol;Acc:MGI:1 095 412]
Gm12239	9.70E-68	1.08 440 517	0.581	0.242	3.13E-63	predicted gene 12 239 [Source:MGI Symbol;Acc:MGI:3 651 547]
C4b	1.35E-67	−0.5100712	0.066	0.312	4.35E-63	complement component 4B (Chido blood group) [Source:MGI Symbol;Acc:MGI:88 228]
Tmem108	2.42E-67	−0.522789	0.043	0.245	7.80E-63	transmembrane protein 108 [Source:MGI Symbol;Acc:MGI:1 932 411]
Dock10	2.55E-66	−0.617134	0.105	0.266	8.24E-62	dedicator of cytokinesis 10 [Source:MGI Symbol;Acc:MGI:2 146 320]
Gm4876	1.07E-65	−0.6175802	0.128	0.368	3.45E-61	predicted gene 4876 [Source:MGI Symbol;Acc:MGI:3 647 654]
Sulf2	1.90E-64	−0.4298459	0.034	0.238	6.14E-60	sulfatase 2 [Source:MGI Symbol;Acc:MGI:1 919 293]
Col23a1	2.45E-64	−0.812356	0.112	0.34	7.92E-60	collagen, type XXIII, alpha 1 [Source:MGI Symbol;Acc:MGI:2 653 243]
Prkag2	6.39E-63	−0.5685136	0.156	0.376	2.06E-58	protein kinase, AMP-activated, gamma 2 non-catalytic subunit [Source:MGI Symbol;Acc:MGI:1 336 153]
Ctnna2	2.00E-61	−0.8570844	0.669	0.809	6.45E-57	catenin (cadherin associated protein), alpha 2 [Source:MGI Symbol;Acc:MGI:88 275]
Myoc	1.78E-60	−0.5794284	0.011	0.188	5.74E-56	myocilin [Source:MGI Symbol;Acc:MGI:1 202 864]
Enpp2	2.58E-60	−0.5283773	0.212	0.453	8.33E-56	ectonucleotide pyrophosphatase/phosphodiesterase 2 [Source:MGI Symbol;Acc:MGI:1 321 390]
Frmd5	6.05E-60	−0.6754012	0.103	0.255	1.95E-55	FERM domain containing 5 [Source:MGI Symbol;Acc:MGI:2 442 557]
Sema6a	9.70E-60	−0.4141433	0.059	0.283	3.13E-55	sema domain, transmembrane domain (TM), and cytoplasmic domain, (semaphorin) 6A [Source:MGI Symbol;Acc:MGI:1 203 727]
Arhgef4	1.15E-59	−0.7358996	0.565	0.697	3.70E-55	Rho guanine nucleotide exchange factor (GEF) 4 [Source:MGI Symbol;Acc:MGI:2 442 507]
Zfp536	5.69E-59	−0.5264944	0.044	0.204	1.84E-54	zinc finger protein 536 [Source:MGI Symbol;Acc:MGI:1 926 102]
Enox1	1.09E-58	−0.7117745	0.249	0.504	3.51E-54	ecto-NOX disulfide-thiol exchanger 1 [Source:MGI Symbol;Acc:MGI:2 444 896]
Pakap	8.14E-57	−0.420385	0.033	0.214	2.63E-52	paralemmin A kinase anchor protein [Source:MGI Symbol;Acc:MGI:5 141 924]
Man1c1	1.77E-56	−0.4697253	0.059	0.256	5.71E-52	mannosidase, alpha, class 1C, member 1 [Source:MGI Symbol;Acc:MGI:2 446 214]
Nrp2	2.58E-56	−0.3914145	0.043	0.236	8.32E-52	neuropilin 2 [Source:MGI Symbol;Acc:MGI:1 100 492]
Csmd1	4.93E-56	−0.5926443	0.386	0.544	1.59E-51	CUB and Sushi multiple domains 1 [Source:MGI Symbol;Acc:MGI:2 137 383]
Nhsl1	7.81E-56	0.7 363 414	0.737	0.443	2.52E-51	NHS-like 1 [Source:MGI Symbol;Acc:MGI:106 390]
Cd44	3.70E-55	−0.432558	0.022	0.198	1.19E-50	CD44 antigen [Source:MGI Symbol;Acc:MGI:88 338]
Dnm3	1.96E-54	−0.5668362	0.281	0.511	6.32E-50	dynamin 3 [Source:MGI Symbol;Acc:MGI:1 341 299]
Pde4b	2.27E-54	−0.765601	0.548	0.506	7.32E-50	phosphodiesterase 4B, cAMP specific [Source:MGI Symbol;Acc:MGI:99 557]
Atp1a2	6.26E-54	0.67 797 427	0.938	0.811	2.02E-49	ATPase, Na+/K + transporting, alpha 2 polypeptide [Source:MGI Symbol;Acc:MGI:88 106]
Pacrg	7.92E-54	−0.6165849	0.279	0.547	2.56E-49	PARK2 co-regulated [Source:MGI Symbol;Acc:MGI:1 916 560]
Rgs7	1.69E-53	0.75 771 718	0.653	0.342	5.44E-49	regulator of G protein signaling 7 [Source:MGI Symbol;Acc:MGI:1 346 089]
Pitpnc1	1.79E-53	0.77 375 072	0.916	0.761	5.79E-49	phosphatidylinositol transfer protein, cytoplasmic 1 [Source:MGI Symbol;Acc:MGI:1 919 045]
Pde7b	2.22E-53	0.75 004 257	0.713	0.379	7.18E-49	phosphodiesterase 7B [Source:MGI Symbol;Acc:MGI:1 352 752]
Sorbs1	4.64E-53	−0.7089635	0.633	0.779	1.50E-48	sorbin and SH3 domain containing 1 [Source:MGI Symbol;Acc:MGI:700 014]
Kif21a	6.38E-53	−0.497393	0.388	0.623	2.06E-48	kinesin family member 21A [Source:MGI Symbol;Acc:MGI:109 188]
Ccser1	9.85E-53	−0.6570347	0.276	0.446	3.18E-48	coiled-coil serine rich 1 [Source:MGI Symbol;Acc:MGI:3 045 354]
Adgrb3	1.78E-52	0.71 451 476	0.96	0.89	5.76E-48	adhesion G protein-coupled receptor B3 [Source:MGI Symbol;Acc:MGI:2 441 837]
Sntg2	4.21E-52	−0.3433437	0.015	0.191	1.36E-47	syntrophin, gamma 2 [Source:MGI Symbol;Acc:MGI:1 919 541]
Mbp	6.86E-52	−0.597413	0.376	0.513	2.21E-47	myelin basic protein [Source:MGI Symbol;Acc:MGI:96 925]
Tmem132b	3.47E-51	−0.3404389	0.062	0.264	1.12E-46	transmembrane protein 132B [Source:MGI Symbol;Acc:MGI:3 609 245]
Phyhd1	2.24E-50	−0.5059512	0.221	0.452	7.24E-46	phytanoyl-CoA dioxygenase domain containing 1 [Source:MGI Symbol;Acc:MGI:3 612 860]
Glis3	3.59E-50	−0.6254211	0.524	0.715	1.16E-45	GLIS family zinc finger 3 [Source:MGI Symbol;Acc:MGI:2 444 289]
Elmo1	7.87E-50	−0.5288191	0.104	0.268	2.54E-45	engulfment and cell motility 1 [Source:MGI Symbol;Acc:MGI:2 153 044]
Car10	2.40E-49	0.85 983 394	0.494	0.282	7.75E-45	carbonic anhydrase 10 [Source:MGI Symbol;Acc:MGI:1 919 855]
Ptch1	3.37E-49	0.70 455 439	0.581	0.285	1.09E-44	patched 1 [Source:MGI Symbol;Acc:MGI:105 373]
Aebp1	1.40E-48	−0.3410028	0.011	0.182	4.52E-44	AE binding protein 1 [Source:MGI Symbol;Acc:MGI:1 197 012]
Pcdh7	2.00E-48	0.5 983 106	0.886	0.744	6.47E-44	protocadherin 7 [Source:MGI Symbol;Acc:MGI:1 860 487]
Dok6	6.61E-48	−0.5449044	0.156	0.382	2.13E-43	docking protein 6 [Source:MGI Symbol;Acc:MGI:3 639 495]
Garnl3	1.52E-47	−0.3735695	0.083	0.266	4.90E-43	GTPase activating RANGAP domain-like 3 [Source:MGI Symbol;Acc:MGI:2 139 309]
Robo2	1.53E-47	−0.6783839	0.565	0.728	4.95E-43	roundabout guidance receptor 2 [Source:MGI Symbol;Acc:MGI:1 890 110]
Synpo2	4.98E-47	−0.6241953	0.162	0.393	1.61E-42	synaptopodin 2 [Source:MGI Symbol;Acc:MGI:2 153 070]
Zeb2	5.73E-46	−0.5489778	0.514	0.657	1.85E-41	zinc finger E-box binding homeobox 2 [Source:MGI Symbol;Acc:MGI:1 344 407]
Padi2	5.77E-46	−0.4531858	0.124	0.336	1.86E-41	peptidyl arginine deiminase, type II [Source:MGI Symbol;Acc:MGI:1 338 892]
C530044C16Rik	5.84E-46	−0.3439918	0.011	0.162	1.89E-41	RIKEN cDNA C530044C16 gene [Source:MGI Symbol;Acc:MGI:2 443 109]
Reep1	2.13E-44	−0.4485735	0.071	0.239	6.88E-40	receptor accessory protein 1 [Source:MGI Symbol;Acc:MGI:1 098 827]
Map7	6.02E-44	−0.4631434	0.147	0.298	1.94E-39	microtubule-associated protein 7 [Source:MGI Symbol;Acc:MGI:1 328 328]
Atp13a4	6.00E-43	0.52 133 603	0.498	0.219	1.94E-38	ATPase type 13A4 [Source:MGI Symbol;Acc:MGI:1 924 456]
Cpne8	3.12E-42	−0.2962867	0.035	0.192	1.01E-37	copine VIII [Source:MGI Symbol;Acc:MGI:1 914 121]
Tagln3	3.76E-42	−0.4510063	0.297	0.487	1.21E-37	transgelin 3 [Source:MGI Symbol;Acc:MGI:1 926 784]
Frmd4a	4.71E-42	0.63 758 173	0.935	0.782	1.52E-37	FERM domain containing 4A [Source:MGI Symbol;Acc:MGI:1 919 850]
Sema3a	7.94E-42	−0.3742641	0.031	0.174	2.56E-37	sema domain, immunoglobulin domain (Ig), short basic domain, secreted, (semaphorin) 3A [Source:MGI Symbol;Acc:MGI:107 558]
Tnc	1.52E-41	−0.2898748	0.019	0.177	4.89E-37	tenascin C [Source:MGI Symbol;Acc:MGI:101 922]
Cacnb4	5.46E-41	−0.5538659	0.231	0.423	1.76E-36	calcium channel, voltage-dependent, beta 4 subunit [Source:MGI Symbol;Acc:MGI:103 301]
Neat1	6.65E-41	−0.6480804	0.521	0.627	2.15E-36	nuclear paraspeckle assembly transcript 1 (non-protein coding) [Source:MGI Symbol;Acc:MGI:1 914 211]
Ano4	3.04E-40	−0.3717007	0.074	0.209	9.82E-36	anoctamin 4 [Source:MGI Symbol;Acc:MGI:2 443 344]
Adamts9	4.01E-40	−0.5152188	0.195	0.419	1.29E-35	a disintegrin-like and metallopeptidase (reprolysin type) with thrombospondin type 1 motif, 9 [Source:MGI Symbol;Acc:MGI:1 916 320]
Luzp2	4.27E-40	0.98 895 087	0.903	0.681	1.38E-35	leucine zipper protein 2 [Source:MGI Symbol;Acc:MGI:1 889 615]
March1	6.66E-40	−0.415775	0.085	0.221	2.15E-35	NA
Igfbp5	7.96E-40	−0.4011506	0.092	0.281	2.57E-35	insulin-like growth factor binding protein 5 [Source:MGI Symbol;Acc:MGI:96 440]
Hecw2	9.58E-40	−0.3916858	0.09	0.215	3.09E-35	HECT, C2 and WW domain containing E3 ubiquitin protein ligase 2 [Source:MGI Symbol;Acc:MGI:2 685 817]
Ank2	1.52E-39	−0.6975627	0.954	0.949	4.92E-35	ankyrin 2, brain [Source:MGI Symbol;Acc:MGI:88 025]
Trp63	2.59E-39	0.64 506 946	0.324	0.111	8.35E-35	transformation related protein 63 [Source:MGI Symbol;Acc:MGI:1 330 810]
Adgrl3	2.95E-39	0.53 274 227	0.906	0.796	9.52E-35	adhesion G protein-coupled receptor L3 [Source:MGI Symbol;Acc:MGI:2 441 950]
9530026P05Rik	1.12E-38	−0.5969957	0.225	0.422	3.61E-34	RIKEN cDNA 9530026P05 gene [Source:MGI Symbol;Acc:MGI:1 924 659]
Fmn1	2.01E-38	−0.5751858	0.195	0.358	6.49E-34	formin 1 [Source:MGI Symbol;Acc:MGI:101 815]
D7Ertd443e	3.39E-38	−0.2901466	0.026	0.16	1.09E-33	DNA segment, Chr 7, ERATO Doi 443, expressed [Source:MGI Symbol;Acc:MGI:1 196 431]
Tex11	7.26E-38	−0.2560236	0.011	0.15	2.34E-33	testis expressed gene 11 [Source:MGI Symbol;Acc:MGI:1 933 237]
Sema6d	1.10E-37	−0.7374482	0.528	0.664	3.54E-33	sema domain, transmembrane domain (TM), and cytoplasmic domain, (semaphorin) 6D [Source:MGI Symbol;Acc:MGI:2 387 661]
Tulp4	2.02E-37	−0.4493122	0.379	0.563	6.51E-33	tubby like protein 4 [Source:MGI Symbol;Acc:MGI:1 916 092]
Aqp4	3.92E-37	−0.495628	0.404	0.593	1.27E-32	aquaporin 4 [Source:MGI Symbol;Acc:MGI:107 387]
Dscaml1	6.17E-37	−0.3247857	0.031	0.145	1.99E-32	DS cell adhesion molecule like 1 [Source:MGI Symbol;Acc:MGI:2 150 309]
Eya4	9.63E-37	−0.3663993	0.093	0.268	3.11E-32	EYA transcriptional coactivator and phosphatase 4 [Source:MGI Symbol;Acc:MGI:1 337 104]
Thbs4	2.03E-36	−0.2864441	0.007	0.128	6.56E-32	thrombospondin 4 [Source:MGI Symbol;Acc:MGI:1 101 779]
Apbb1ip	2.03E-36	−0.2760399	0.012	0.164	6.57E-32	amyloid beta (A4) precursor protein-binding, family B, member 1 interacting protein [Source:MGI Symbol;Acc:MGI:1 861 354]
Ank3	2.07E-36	−0.4310119	0.238	0.366	6.67E-32	ankyrin 3, epithelial [Source:MGI Symbol;Acc:MGI:88 026]
Slc4a4	2.32E-36	0.53 869 848	0.927	0.846	7.47E-32	solute carrier family 4 (anion exchanger), member 4 [Source:MGI Symbol;Acc:MGI:1 927 555]
Syne1	2.48E-36	0.48 914 007	0.884	0.774	8.02E-32	spectrin repeat containing, nuclear envelope 1 [Source:MGI Symbol;Acc:MGI:1 927 152]
Cadm2	8.59E-36	0.47 857 272	0.992	0.92	2.77E-31	cell adhesion molecule 2 [Source:MGI Symbol;Acc:MGI:2 442 722]
Pxdn	3.14E-35	−0.2916801	0.071	0.222	1.02E-30	peroxidasin [Source:MGI Symbol;Acc:MGI:1 916 925]
Cmya5	4.19E-35	−0.3340131	0.093	0.272	1.35E-30	cardiomyopathy associated 5 [Source:MGI Symbol;Acc:MGI:1 923 719]
4930402H24Rik	5.42E-35	−0.4868307	0.83	0.86	1.75E-30	NA
Reln	6.39E-35	−0.4320311	0.11	0.295	2.06E-30	reelin [Source:MGI Symbol;Acc:MGI:103 022]
Retreg1	9.38E-35	−0.2923592	0.096	0.266	3.03E-30	reticulophagy regulator 1 [Source:MGI Symbol;Acc:MGI:1 913 520]
Gpld1	1.38E-34	0.46 185 208	0.395	0.151	4.47E-30	glycosylphosphatidylinositol specific phospholipase D1 [Source:MGI Symbol;Acc:MGI:106 604]
Plp1	1.47E-34	−0.434856	0.599	0.731	4.76E-30	proteolipid protein (myelin) 1 [Source:MGI Symbol;Acc:MGI:97 623]
Mapt	2.50E-34	−0.4417669	0.427	0.56	8.06E-30	microtubule-associated protein tau [Source:MGI Symbol;Acc:MGI:97 180]
Eps8	2.62E-34	0.47 960 348	0.537	0.274	8.46E-30	epidermal growth factor receptor pathway substrate 8 [Source:MGI Symbol;Acc:MGI:104 684]
Tab2	3.98E-34	−0.3833314	0.251	0.45	1.28E-29	TGF-beta activated kinase 1/MAP3K7 binding protein 2 [Source:MGI Symbol;Acc:MGI:1 915 902]
Dock4	4.61E-34	0.53 604 373	0.815	0.641	1.49E-29	dedicator of cytokinesis 4 [Source:MGI Symbol;Acc:MGI:1 918 006]
Ptprk	2.18E-33	−0.3266188	0.086	0.219	7.02E-29	protein tyrosine phosphatase, receptor type, K [Source:MGI Symbol;Acc:MGI:103 310]
Airn	4.11E-33	−0.3179305	0.054	0.184	1.33E-28	antisense Igf2r RNA [Source:MGI Symbol;Acc:MGI:1 353 471]
Erbin	2.15E-32	−0.4181598	0.299	0.46	6.94E-28	Erbb2 interacting protein [Source:MGI Symbol;Acc:MGI:1 890 169]
Plxdc2	2.72E-32	−0.4398	0.267	0.443	8.77E-28	plexin domain containing 2 [Source:MGI Symbol;Acc:MGI:1 914 698]
Vav3	3.24E-32	0.49 950 379	0.446	0.187	1.05E-27	vav 3 oncogene [Source:MGI Symbol;Acc:MGI:1 888 518]
Sh3d19	4.83E-32	−0.2590898	0.041	0.171	1.56E-27	SH3 domain protein D19 [Source:MGI Symbol;Acc:MGI:1 350 923]
Gm10635	6.44E-32	−0.3184162	0.023	0.15	2.08E-27	predicted gene 10 635 [Source:MGI Symbol;Acc:MGI:3 641 740]
Itih3	1.22E-31	0.46 778 967	0.342	0.128	3.95E-27	inter-alpha trypsin inhibitor, heavy chain 3 [Source:MGI Symbol;Acc:MGI:96 620]
Kcnma1	1.29E-31	0.58 278 397	0.672	0.416	4.18E-27	potassium large conductance calcium-activated channel, subfamily M, alpha member 1 [Source:MGI Symbol;Acc:MGI:99 923]
Acsbg1	1.66E-31	0.46 229 443	0.606	0.406	5.36E-27	acyl-CoA synthetase bubblegum family member 1 [Source:MGI Symbol;Acc:MGI:2 385 656]
Naaladl2	2.22E-31	−0.5059697	0.314	0.528	7.16E-27	N-acetylated alpha-linked acidic dipeptidase-like 2 [Source:MGI Symbol;Acc:MGI:2 685 867]
Rps6ka5	2.78E-31	−0.3575731	0.204	0.395	8.99E-27	ribosomal protein S6 kinase, polypeptide 5 [Source:MGI Symbol;Acc:MGI:1 920 336]
Mobp	3.94E-31	−0.347979	0.135	0.258	1.27E-26	myelin-associated oligodendrocytic basic protein [Source:MGI Symbol;Acc:MGI:108 511]
Cdh10	9.07E-31	0.49 836 499	0.738	0.536	2.93E-26	cadherin 10 [Source:MGI Symbol;Acc:MGI:107 436]
Cadm1	1.31E-30	0.50 571 336	0.835	0.648	4.24E-26	cell adhesion molecule 1 [Source:MGI Symbol;Acc:MGI:1 889 272]
Arhgap6	1.40E-30	−0.2542343	0.034	0.144	4.51E-26	Rho GTPase activating protein 6 [Source:MGI Symbol;Acc:MGI:1 196 332]
Dner	2.35E-30	−0.344161	0.173	0.33	7.57E-26	delta/notch-like EGF repeat containing [Source:MGI Symbol;Acc:MGI:2 152 889]
Disp3	2.80E-30	−0.3023469	0.077	0.199	9.04E-26	dispatched RND transporter family member 3 [Source:MGI Symbol;Acc:MGI:2 444 403]
Npas3	4.84E-30	0.46 551 269	0.985	0.943	1.56E-25	neuronal PAS domain protein 3 [Source:MGI Symbol;Acc:MGI:1 351 610]
Id3	6.28E-30	−0.340075	0.134	0.312	2.03E-25	inhibitor of DNA binding 3 [Source:MGI Symbol;Acc:MGI:96 398]
Kalrn	6.35E-30	0.44 370 462	0.566	0.321	2.05E-25	kalirin, RhoGEF kinase [Source:MGI Symbol;Acc:MGI:2 685 385]
Fgfr3	1.96E-29	0.41 311 401	0.634	0.457	6.32E-25	fibroblast growth factor receptor 3 [Source:MGI Symbol;Acc:MGI:95 524]
Agap1	2.08E-29	−0.3462155	0.198	0.343	6.72E-25	ArfGAP with GTPase domain, ankyrin repeat and PH domain 1 [Source:MGI Symbol;Acc:MGI:2 653 690]
Bin1	2.61E-29	−0.2968423	0.13	0.262	8.41E-25	bridging integrator 1 [Source:MGI Symbol;Acc:MGI:108 092]
Tspan7	3.06E-29	0.46 649 108	0.784	0.63	9.89E-25	tetraspanin 7 [Source:MGI Symbol;Acc:MGI:1 298 407]
Cables1	3.75E-29	0.43 697 152	0.47	0.218	1.21E-24	CDK5 and Abl enzyme substrate 1 [Source:MGI Symbol;Acc:MGI:1 927 065]
Ddr1	4.57E-29	−0.2918304	0.134	0.288	1.47E-24	discoidin domain receptor family, member 1 [Source:MGI Symbol;Acc:MGI:99 216]
Ano1	4.74E-29	−0.3299227	0.003	0.144	1.53E-24	anoctamin 1, calcium activated chloride channel [Source:MGI Symbol;Acc:MGI:2 142 149]
Grin2c	7.34E-29	0.38 585 443	0.428	0.188	2.37E-24	glutamate receptor, ionotropic, NMDA2C (epsilon 3) [Source:MGI Symbol;Acc:MGI:95 822]
Slco1c1	8.32E-29	0.44 635 941	0.459	0.235	2.69E-24	solute carrier organic anion transporter family, member 1c1 [Source:MGI Symbol;Acc:MGI:1 889 679]
Trim2	9.96E-29	−0.3765173	0.308	0.491	3.21E-24	tripartite motif-containing 2 [Source:MGI Symbol;Acc:MGI:1 933 163]
Fat3	1.02E-28	0.48 826 949	0.685	0.526	3.29E-24	FAT atypical cadherin 3 [Source:MGI Symbol;Acc:MGI:2 444 314]
Spock1	1.36E-28	−0.3010901	0.111	0.228	4.39E-24	sparc/osteonectin, cwcv and kazal-like domains proteoglycan 1 [Source:MGI Symbol;Acc:MGI:105 371]
Grin3a	1.95E-28	−0.3937081	0.138	0.293	6.30E-24	glutamate receptor ionotropic, NMDA3A [Source:MGI Symbol;Acc:MGI:1 933 206]
Ptprg	2.33E-28	0.48 924 426	0.642	0.496	7.51E-24	NA
Nwd1	2.35E-28	0.41 679 627	0.705	0.506	7.59E-24	NACHT and WD repeat domain containing 1 [Source:MGI Symbol;Acc:MGI:2 442 268]
Wwox	3.10E-28	−0.566715	0.526	0.678	1.00E-23	WW domain-containing oxidoreductase [Source:MGI Symbol;Acc:MGI:1 931 237]
Cep85l	6.31E-28	0.4 097 875	0.578	0.382	2.04E-23	centrosomal protein 85-like [Source:MGI Symbol;Acc:MGI:3 642 684]
Gria4	6.44E-28	0.59 105 045	0.426	0.258	2.08E-23	glutamate receptor, ionotropic, AMPA4 (alpha 4) [Source:MGI Symbol;Acc:MGI:95 811]
Nxn	9.83E-28	−0.4726422	0.421	0.595	3.17E-23	nucleoredoxin [Source:MGI Symbol;Acc:MGI:109 331]
2610307P16Rik	1.27E-27	−0.3165983	0.064	0.198	4.11E-23	RIKEN cDNA 2610307P16 gene [Source:MGI Symbol;Acc:MGI:1 919 768]
Ccdc85a	1.29E-27	0.47 682 104	0.524	0.282	4.16E-23	coiled-coil domain containing 85A [Source:MGI Symbol;Acc:MGI:2 445 069]
Grid2	2.01E-27	0.48 400 785	0.91	0.761	6.50E-23	glutamate receptor, ionotropic, delta 2 [Source:MGI Symbol;Acc:MGI:95 813]
Nrbp2	3.89E-27	−0.313165	0.184	0.355	1.25E-22	nuclear receptor binding protein 2 [Source:MGI Symbol;Acc:MGI:2 385 017]
Etv4	6.28E-27	0.34 377 119	0.281	0.073	2.03E-22	ets variant 4 [Source:MGI Symbol;Acc:MGI:99 423]
Cep112	6.56E-27	−0.2566422	0.072	0.225	2.12E-22	centrosomal protein 112 [Source:MGI Symbol;Acc:MGI:1 923 673]
Rnf220	1.42E-26	−0.3829309	0.161	0.242	4.60E-22	ring finger protein 220 [Source:MGI Symbol;Acc:MGI:1 913 993]
Rtn4	3.75E-26	−0.3559138	0.503	0.652	1.21E-21	reticulon 4 [Source:MGI Symbol;Acc:MGI:1 915 835]
Klhl29	4.42E-26	−0.2814585	0.123	0.278	1.43E-21	kelch-like 29 [Source:MGI Symbol;Acc:MGI:2 683 857]
Sorcs1	7.06E-26	−0.3292721	0.112	0.226	2.28E-21	sortilin-related VPS10 domain containing receptor 1 [Source:MGI Symbol;Acc:MGI:1 929 666]
App	8.22E-26	−0.3678775	0.521	0.642	2.65E-21	amyloid beta (A4) precursor protein [Source:MGI Symbol;Acc:MGI:88 059]
Grk3	1.23E-25	0.38 379 014	0.572	0.339	3.97E-21	G protein-coupled receptor kinase 3 [Source:MGI Symbol;Acc:MGI:87 941]
Atp1b2	1.53E-25	0.45 621 018	0.541	0.426	4.95E-21	ATPase, Na+/K + transporting, beta 2 polypeptide [Source:MGI Symbol;Acc:MGI:88 109]
Bmp6	1.82E-25	−0.2566358	0.039	0.16	5.88E-21	bone morphogenetic protein 6 [Source:MGI Symbol;Acc:MGI:88 182]
Ndrg2	1.93E-25	0.40 022 616	0.73	0.62	6.22E-21	N-myc downstream regulated gene 2 [Source:MGI Symbol;Acc:MGI:1 352 498]
Mgat4c	3.09E-25	0.47 103 312	0.565	0.479	9.97E-21	MGAT4 family, member C [Source:MGI Symbol;Acc:MGI:1 914 819]
Pdgfd	5.25E-25	−0.4308435	0.39	0.528	1.69E-20	platelet-derived growth factor, D polypeptide [Source:MGI Symbol;Acc:MGI:1 919 035]
Nav2	6.00E-25	−0.4203342	0.521	0.64	1.94E-20	neuron navigator 2 [Source:MGI Symbol;Acc:MGI:2 183 691]
Shc3	9.06E-25	0.35 170 743	0.387	0.172	2.92E-20	src homology 2 domain-containing transforming protein C3 [Source:MGI Symbol;Acc:MGI:106 179]
Plscr4	1.07E-24	−0.2635147	0.099	0.251	3.44E-20	phospholipid scramblase 4 [Source:MGI Symbol;Acc:MGI:2 143 267]
Dgkb	1.55E-24	0.41 451 406	0.899	0.788	5.02E-20	diacylglycerol kinase, beta [Source:MGI Symbol;Acc:MGI:2 442 474]
Fry	1.97E-24	0.38 056 965	0.677	0.567	6.35E-20	FRY microtubule binding protein [Source:MGI Symbol;Acc:MGI:2 443 895]
Tnik	2.85E-24	0.39 465 172	0.926	0.852	9.19E-20	TRAF2 and NCK interacting kinase [Source:MGI Symbol;Acc:MGI:1 916 264]
Slc1a2	4.57E-24	0.38 376 796	0.999	0.976	1.47E-19	solute carrier family 1 (glial high affinity glutamate transporter), member 2 [Source:MGI Symbol;Acc:MGI:101 931]
Tmc7	4.76E-24	0.36 285 491	0.343	0.152	1.54E-19	transmembrane channel-like gene family 7 [Source:MGI Symbol;Acc:MGI:2 443 317]
Nrcam	5.21E-24	0.38 906 253	0.831	0.684	1.68E-19	neuronal cell adhesion molecule [Source:MGI Symbol;Acc:MGI:104 750]
Pgm5	1.33E-23	−0.2678154	0.034	0.161	4.28E-19	phosphoglucomutase 5 [Source:MGI Symbol;Acc:MGI:1 925 668]
Rapgef4	1.55E-23	−0.3033841	0.123	0.236	5.00E-19	Rap guanine nucleotide exchange factor (GEF) 4 [Source:MGI Symbol;Acc:MGI:1 917 723]
Adipor2	2.96E-23	−0.2924237	0.152	0.241	9.56E-19	adiponectin receptor 2 [Source:MGI Symbol;Acc:MGI:93 830]
Gabbr2	8.65E-23	0.40 045 043	0.33	0.151	2.79E-18	gamma-aminobutyric acid (GABA) B receptor, 2 [Source:MGI Symbol;Acc:MGI:2 386 030]
Phactr3	1.14E-22	0.36 083 155	0.518	0.285	3.68E-18	phosphatase and actin regulator 3 [Source:MGI Symbol;Acc:MGI:1 921 439]
Dst	1.39E-22	−0.3671163	0.621	0.718	4.49E-18	dystonin [Source:MGI Symbol;Acc:MGI:104 627]
Gjb6	1.94E-22	0.31 796 427	0.347	0.164	6.27E-18	gap junction protein, beta 6 [Source:MGI Symbol;Acc:MGI:107 588]
Dip2b	2.13E-22	−0.313708	0.344	0.487	6.89E-18	disco interacting protein 2 homolog B [Source:MGI Symbol;Acc:MGI:2 145 977]
Ntng1	3.12E-22	−0.3157252	0.062	0.177	1.01E-17	netrin G1 [Source:MGI Symbol;Acc:MGI:1 934 028]
Frmpd4	3.81E-22	−0.2667709	0.136	0.234	1.23E-17	FERM and PDZ domain containing 4 [Source:MGI Symbol;Acc:MGI:3 042 378]
Kcnip3	3.99E-22	0.37 212 366	0.377	0.187	1.29E-17	Kv channel interacting protein 3, calsenilin [Source:MGI Symbol;Acc:MGI:1 929 258]
Ppp1r16b	4.87E-22	−0.2635873	0.094	0.174	1.57E-17	protein phosphatase 1, regulatory subunit 16B [Source:MGI Symbol;Acc:MGI:2 151 841]
Slc6a1	7.68E-22	0.35 533 763	0.601	0.442	2.48E-17	solute carrier family 6 (neurotransmitter transporter, GABA), member 1 [Source:MGI Symbol;Acc:MGI:95 627]
Sorcs2	8.98E-22	0.31 923 246	0.349	0.161	2.90E-17	sortilin-related VPS10 domain containing receptor 2 [Source:MGI Symbol;Acc:MGI:1 932 289]
Mpdz	1.18E-21	−0.26438	0.155	0.299	3.81E-17	multiple PDZ domain crumbs cell polarity complex component [Source:MGI Symbol;Acc:MGI:1 343 489]
Tsc22d1	1.35E-21	−0.2744768	0.261	0.415	4.35E-17	TSC22 domain family, member 1 [Source:MGI Symbol;Acc:MGI:109 127]
Xylt1	1.69E-21	0.39 333 349	0.511	0.288	5.45E-17	xylosyltransferase 1 [Source:MGI Symbol;Acc:MGI:2 451 073]
Ski	2.22E-21	−0.287537	0.171	0.312	7.18E-17	ski sarcoma viral oncogene homolog (avian) [Source:MGI Symbol;Acc:MGI:98 310]
Tenm4	2.83E-21	0.39 167 545	0.423	0.251	9.12E-17	teneurin transmembrane protein 4 [Source:MGI Symbol;Acc:MGI:2 447 063]
Ablim1	2.84E-21	0.35 555 579	0.517	0.315	9.15E-17	actin-binding LIM protein 1 [Source:MGI Symbol;Acc:MGI:1 194 500]
Ahcyl1	3.24E-21	0.36 060 642	0.668	0.504	1.05E-16	S-adenosylhomocysteine hydrolase-like 1 [Source:MGI Symbol;Acc:MGI:2 385 184]
Shroom3	5.82E-21	0.39 782 592	0.457	0.319	1.88E-16	shroom family member 3 [Source:MGI Symbol;Acc:MGI:1 351 655]
Mertk	1.09E-20	0.43 718 999	0.798	0.638	3.51E-16	MER proto-oncogene tyrosine kinase [Source:MGI Symbol;Acc:MGI:96 965]
Wasf3	1.16E-20	0.33 069 633	0.551	0.362	3.75E-16	WASP family, member 3 [Source:MGI Symbol;Acc:MGI:2 658 986]
Myo16	2.10E-20	−0.2681525	0.095	0.209	6.78E-16	myosin XVI [Source:MGI Symbol;Acc:MGI:2 685 951]
9630028H03Rik	2.28E-20	0.32 473 426	0.304	0.125	7.38E-16	RIKEN cDNA 9630028H03 gene [Source:MGI Symbol;Acc:MGI:2 444 526]
Plekha5	4.64E-20	−0.2723481	0.204	0.352	1.50E-15	pleckstrin homology domain containing, family A member 5 [Source:MGI Symbol;Acc:MGI:1 923 802]
Npas2	4.88E-20	0.38 493 547	0.571	0.389	1.58E-15	neuronal PAS domain protein 2 [Source:MGI Symbol;Acc:MGI:109 232]
St3gal4	1.26E-19	0.3 840 103	0.546	0.343	4.07E-15	ST3 beta-galactoside alpha-2,3-sialyltransferase 4 [Source:MGI Symbol;Acc:MGI:1 316 743]
Dmd	1.92E-19	0.42 285 619	0.824	0.698	6.19E-15	dystrophin, muscular dystrophy [Source:MGI Symbol;Acc:MGI:94 909]
Myo5a	1.96E-19	−0.2638717	0.209	0.335	6.32E-15	myosin VA [Source:MGI Symbol;Acc:MGI:105 976]
Ralgps2	3.83E-19	−0.2590348	0.168	0.316	1.24E-14	Ral GEF with PH domain and SH3 binding motif 2 [Source:MGI Symbol;Acc:MGI:1 925 505]
Tprkb	5.94E-19	−0.3453149	0.36	0.504	1.92E-14	Tp53rk binding protein [Source:MGI Symbol;Acc:MGI:1 917 036]
Plcl1	6.13E-19	−0.4096364	0.585	0.521	1.98E-14	phospholipase C-like 1 [Source:MGI Symbol;Acc:MGI:3 036 262]
Mgat5	6.17E-19	−0.3184611	0.326	0.486	1.99E-14	mannoside acetylglucosaminyltransferase 5 [Source:MGI Symbol;Acc:MGI:894 701]
Htra1	7.27E-19	0.36 090 744	0.662	0.472	2.35E-14	HtrA serine peptidase 1 [Source:MGI Symbol;Acc:MGI:1 929 076]
Vegfa	7.45E-19	0.35 819 241	0.539	0.373	2.41E-14	vascular endothelial growth factor A [Source:MGI Symbol;Acc:MGI:103 178]
Nim1k	1.14E-18	0.321 931	0.413	0.241	3.68E-14	NIM1 serine/threonine protein kinase [Source:MGI Symbol;Acc:MGI:2 442 399]
Nrg2	1.19E-18	−0.3042189	0.223	0.373	3.84E-14	neuregulin 2 [Source:MGI Symbol;Acc:MGI:1 098 246]
Hif1a	1.93E-18	0.27 065 116	0.274	0.138	6.25E-14	hypoxia inducible factor 1, alpha subunit [Source:MGI Symbol;Acc:MGI:106 918]
Mical2	2.68E-18	0.32 752 013	0.648	0.483	8.64E-14	microtubule associated monooxygenase, calponin and LIM domain containing 2 [Source:MGI Symbol;Acc:MGI:2 444 947]
Lhfpl3	3.27E-18	−0.3983802	0.291	0.375	1.05E-13	lipoma HMGIC fusion partner-like 3 [Source:MGI Symbol;Acc:MGI:1 925 076]
Ntm	3.91E-18	0.2 944 428	0.995	0.97	1.26E-13	neurotrimin [Source:MGI Symbol;Acc:MGI:2 446 259]
Tafa1	4.53E-18	0.48 678 136	0.311	0.165	1.46E-13	TAFA chemokine like family member 1 [Source:MGI Symbol;Acc:MGI:2 443 695]
Arid5b	5.38E-18	−0.2622279	0.142	0.262	1.74E-13	AT rich interactive domain 5B (MRF1-like) [Source:MGI Symbol;Acc:MGI:2 175 912]
Esrrg	6.07E-18	0.36 245 516	0.491	0.368	1.96E-13	estrogen-related receptor gamma [Source:MGI Symbol;Acc:MGI:1 347 056]
Ankrd28	6.58E-18	−0.2947165	0.341	0.483	2.13E-13	ankyrin repeat domain 28 [Source:MGI Symbol;Acc:MGI:2 145 661]
Wdr17	7.20E-18	0.40 737 087	0.844	0.751	2.32E-13	WD repeat domain 17 [Source:MGI Symbol;Acc:MGI:1 924 662]
Fus	7.50E-18	−0.2719157	0.364	0.511	2.42E-13	fused in sarcoma [Source:MGI Symbol;Acc:MGI:1 353 633]
Dpyd	8.99E-18	−0.29758	0.254	0.385	2.90E-13	dihydropyrimidine dehydrogenase [Source:MGI Symbol;Acc:MGI:2 139 667]
Stard13	1.30E-17	−0.3748371	0.375	0.541	4.20E-13	StAR-related lipid transfer (START) domain containing 13 [Source:MGI Symbol;Acc:MGI:2 385 331]
Adcy8	1.36E-17	−0.3060634	0.241	0.349	4.41E-13	adenylate cyclase 8 [Source:MGI Symbol;Acc:MGI:1 341 110]
Ptprd	1.76E-17	−0.324527	0.848	0.848	5.68E-13	protein tyrosine phosphatase, receptor type, D [Source:MGI Symbol;Acc:MGI:97 812]
9630014M24Rik	2.04E-17	0.26 701 295	0.273	0.105	6.58E-13	RIKEN cDNA 9630014M24 gene [Source:MGI Symbol;Acc:MGI:3 588 234]
Btbd9	2.29E-17	−0.2898079	0.336	0.486	7.39E-13	BTB (POZ) domain containing 9 [Source:MGI Symbol;Acc:MGI:1 916 625]
Auts2	2.68E-17	0.3 804 811	0.911	0.802	8.65E-13	autism susceptibility candidate 2 [Source:MGI Symbol;Acc:MGI:1 919 847]
Arhgap23	3.14E-17	−0.2714286	0.234	0.322	1.01E-12	Rho GTPase activating protein 23 [Source:MGI Symbol;Acc:MGI:3 697 726]
Pde10a	3.38E-17	0.58 969 939	0.404	0.234	1.09E-12	phosphodiesterase 10A [Source:MGI Symbol;Acc:MGI:1 345 143]
Grip1	4.32E-17	0.31 380 716	0.281	0.134	1.40E-12	glutamate receptor interacting protein 1 [Source:MGI Symbol;Acc:MGI:1 921 303]
St6galnac5	7.24E-17	0.32 665 322	0.255	0.104	2.34E-12	ST6 (alpha-N-acetyl-neuraminyl-2,3-beta-galactosyl-1,3)-N-acetylgalactosaminide alpha-2,6-sialyltransferase 5 [Source:MGI Symbol;Acc:MGI:1 349 471]
Ctnnd2	7.33E-17	0.29 063 944	0.981	0.959	2.37E-12	catenin (cadherin associated protein), delta 2 [Source:MGI Symbol;Acc:MGI:1 195 966]
Map2k6	7.54E-17	0.2 867 068	0.382	0.205	2.43E-12	mitogen-activated protein kinase kinase 6 [Source:MGI Symbol;Acc:MGI:1 346 870]
Gm35188	1.00E-16	0.33 492 285	0.642	0.477	3.23E-12	predicted gene, 35 188 [Source:MGI Symbol;Acc:MGI:5 594 347]
Kmt2c	1.20E-16	0.32 629 187	0.649	0.543	3.89E-12	lysine (K)-specific methyltransferase 2C [Source:MGI Symbol;Acc:MGI:2 444 959]
Eda	1.43E-16	−0.3156606	0.21	0.356	4.62E-12	ectodysplasin-A [Source:MGI Symbol;Acc:MGI:1 195 272]
Egfr	1.70E-16	0.3 217 737	0.458	0.258	5.49E-12	epidermal growth factor receptor [Source:MGI Symbol;Acc:MGI:95 294]
Negr1	2.09E-16	0.37 537 639	0.853	0.697	6.76E-12	neuronal growth regulator 1 [Source:MGI Symbol;Acc:MGI:2 444 846]
Chd9	2.70E-16	0.31 204 806	0.773	0.66	8.72E-12	chromodomain helicase DNA binding protein 9 [Source:MGI Symbol;Acc:MGI:1 924 001]
4930545L23Rik	3.15E-16	0.2 525 562	0.213	0.075	1.02E-11	RIKEN cDNA 4930545L23 gene [Source:MGI Symbol;Acc:MGI:1 926 055]
Mbnl2	3.57E-16	−0.2971172	0.584	0.687	1.15E-11	muscleblind like splicing factor 2 [Source:MGI Symbol;Acc:MGI:2 145 597]
Cdc42bpa	3.90E-16	−0.2882029	0.566	0.645	1.26E-11	CDC42 binding protein kinase alpha [Source:MGI Symbol;Acc:MGI:2 441 841]
Rgs7bp	5.91E-16	0.28 765 137	0.344	0.214	1.91E-11	regulator of G-protein signalling 7 binding protein [Source:MGI Symbol;Acc:MGI:106 334]
Hip1	6.79E-16	0.31 045 931	0.482	0.302	2.19E-11	huntingtin interacting protein 1 [Source:MGI Symbol;Acc:MGI:1 099 804]
Kcnj10	7.30E-16	0.28 764 876	0.358	0.238	2.36E-11	potassium inwardly-rectifying channel, subfamily J, member 10 [Source:MGI Symbol;Acc:MGI:1 194 504]
Gab1	8.69E-16	−0.2510665	0.229	0.339	2.80E-11	growth factor receptor bound protein 2-associated protein 1 [Source:MGI Symbol;Acc:MGI:108 088]
Limch1	1.22E-15	0.3 424 342	0.676	0.507	3.95E-11	LIM and calponin homology domains 1 [Source:MGI Symbol;Acc:MGI:1 924 819]
Gm30382	1.52E-15	0.27 365 545	0.168	0.058	4.92E-11	predicted gene, 30 382 [Source:MGI Symbol;Acc:MGI:5 589 541]
Aldoc	1.70E-15	0.27 040 773	0.489	0.356	5.48E-11	aldolase C, fructose-bisphosphate [Source:MGI Symbol;Acc:MGI:101 863]
Wwc1	1.81E-15	0.30 910 571	0.553	0.403	5.85E-11	WW, C2 and coiled-coil domain containing 1 [Source:MGI Symbol;Acc:MGI:2 388 637]
Phka1	1.91E-15	0.35 229 305	0.647	0.521	6.17E-11	phosphorylase kinase alpha 1 [Source:MGI Symbol;Acc:MGI:97 576]
Map4k4	1.93E-15	−0.2665458	0.405	0.524	6.22E-11	mitogen-activated protein kinase kinase kinase kinase 4 [Source:MGI Symbol;Acc:MGI:1 349 394]
Stk32a	2.16E-15	−0.4253767	0.227	0.343	6.99E-11	serine/threonine kinase 32A [Source:MGI Symbol;Acc:MGI:2 442 403]
Igsf11	2.48E-15	−0.269838	0.342	0.48	8.01E-11	immunoglobulin superfamily, member 11 [Source:MGI Symbol;Acc:MGI:2 388 477]
Utrn	2.56E-15	−0.3520944	0.596	0.647	8.26E-11	utrophin [Source:MGI Symbol;Acc:MGI:104 631]
Prkn	2.62E-15	−0.35671	0.592	0.715	8.46E-11	parkin RBR E3 ubiquitin protein ligase [Source:MGI Symbol;Acc:MGI:1 355 296]
Syn2	3.73E-15	0.29 810 587	0.52	0.392	1.21E-10	synapsin II [Source:MGI Symbol;Acc:MGI:103 020]
Dbx2	4.19E-15	0.25 917 693	0.37	0.226	1.35E-10	developing brain homeobox 2 [Source:MGI Symbol;Acc:MGI:107 445]
Gm6145	4.52E-15	0.30 080 397	0.641	0.5	1.46E-10	predicted gene 6145 [Source:MGI Symbol;Acc:MGI:3 779 559]
Id4	4.92E-15	−0.2927395	0.274	0.429	1.59E-10	inhibitor of DNA binding 4 [Source:MGI Symbol;Acc:MGI:99 414]
Dpf3	5.68E-15	0.2 822 158	0.437	0.283	1.83E-10	double PHD fingers 3 [Source:MGI Symbol;Acc:MGI:1 917 377]
F3	8.81E-15	0.30 629 678	0.542	0.423	2.84E-10	coagulation factor III [Source:MGI Symbol;Acc:MGI:88 381]
Sat1	2.02E-14	0.28 493 381	0.424	0.288	6.51E-10	spermidine/spermine N1-acetyl transferase 1 [Source:MGI Symbol;Acc:MGI:98 233]
Pbx1	2.05E-14	−0.3281921	0.9	0.897	6.62E-10	pre B cell leukemia homeobox 1 [Source:MGI Symbol;Acc:MGI:97 495]
Tmem178b	2.22E-14	−0.3464875	0.398	0.484	7.18E-10	transmembrane protein 178B [Source:MGI Symbol;Acc:MGI:3 647 581]
Ghr	2.76E-14	0.30 505 469	0.492	0.338	8.90E-10	growth hormone receptor [Source:MGI Symbol;Acc:MGI:95 708]
Ptprz1	2.79E-14	0.29 632 444	0.898	0.839	9.02E-10	protein tyrosine phosphatase, receptor type Z, polypeptide 1 [Source:MGI Symbol;Acc:MGI:97 816]
Gm16168	2.81E-14	−0.2524413	0.199	0.306	9.08E-10	predicted gene 16 168 [Source:MGI Symbol;Acc:MGI:3 802 010]
Arhgap26	3.02E-14	0.30 202 183	0.534	0.4	9.74E-10	Rho GTPase activating protein 26 [Source:MGI Symbol;Acc:MGI:1 918 552]
Etnppl	3.68E-14	0.32 550 823	0.401	0.249	1.19E-09	ethanolamine phosphate phospholyase [Source:MGI Symbol;Acc:MGI:1 919 010]
Slc7a2	3.76E-14	−0.2692809	0.305	0.417	1.22E-09	solute carrier family 7 (cationic amino acid transporter, y + system), member 2 [Source:MGI Symbol;Acc:MGI:99 828]
Ube2e2	4.03E-14	−0.3036555	0.478	0.601	1.30E-09	ubiquitin-conjugating enzyme E2E 2 [Source:MGI Symbol;Acc:MGI:2 384 997]
Gli3	4.62E-14	0.28 367 684	0.699	0.573	1.49E-09	GLI-Kruppel family member GLI3 [Source:MGI Symbol;Acc:MGI:95 729]
Ccdc141	4.83E-14	0.2 782 979	0.467	0.338	1.56E-09	coiled-coil domain containing 141 [Source:MGI Symbol;Acc:MGI:1 919 735]
Tmtc2	4.83E-14	0.36 794 504	0.573	0.369	1.56E-09	transmembrane and tetratricopeptide repeat containing 2 [Source:MGI Symbol;Acc:MGI:1 914 057]
Immp2l	6.36E-14	−0.2688214	0.185	0.312	2.05E-09	IMP2 inner mitochondrial membrane peptidase-like (S. cerevisiae) [Source:MGI Symbol;Acc:MGI:2 135 611]
Tenm3	7.88E-14	−0.3230642	0.527	0.657	2.54E-09	teneurin transmembrane protein 3 [Source:MGI Symbol;Acc:MGI:1 345 183]
Agl	8.71E-14	0.25 181 808	0.421	0.289	2.81E-09	amylo-1,6-glucosidase, 4-alpha-glucanotransferase [Source:MGI Symbol;Acc:MGI:1 924 809]
Phkg1	8.88E-14	0.27 904 117	0.679	0.573	2.87E-09	phosphorylase kinase gamma 1 [Source:MGI Symbol;Acc:MGI:97 579]
Nbea	9.70E-14	−0.2588562	0.513	0.611	3.13E-09	neurobeachin [Source:MGI Symbol;Acc:MGI:1 347 075]
Smyd3	1.16E-13	−0.2825564	0.35	0.497	3.74E-09	SET and MYND domain containing 3 [Source:MGI Symbol;Acc:MGI:1 916 976]
Chst11	1.30E-13	−0.2610528	0.259	0.37	4.21E-09	carbohydrate sulfotransferase 11 [Source:MGI Symbol;Acc:MGI:1 927 166]
Ptgds	1.42E-13	−0.2725734	0.386	0.469	4.57E-09	prostaglandin D2 synthase (brain) [Source:MGI Symbol;Acc:MGI:99 261]
Bmpr1b	1.51E-13	−0.3168801	0.67	0.741	4.86E-09	bone morphogenetic protein receptor, type 1B [Source:MGI Symbol;Acc:MGI:107 191]
Rmst	1.63E-13	−0.3892972	0.755	0.795	5.26E-09	rhabdomyosarcoma 2 associated transcript (non-coding RNA) [Source:MGI Symbol;Acc:MGI:1 099 806]
Camk1d	1.68E-13	0.30 376 059	0.674	0.519	5.42E-09	calcium/calmodulin-dependent protein kinase ID [Source:MGI Symbol;Acc:MGI:2 442 190]
Ttc28	1.69E-13	−0.2612668	0.364	0.486	5.45E-09	tetratricopeptide repeat domain 28 [Source:MGI Symbol;Acc:MGI:2 140 873]
Zfp462	1.76E-13	−0.2739835	0.325	0.446	5.67E-09	zinc finger protein 462 [Source:MGI Symbol;Acc:MGI:107 690]
Pde8b	2.74E-13	0.29 540 452	0.43	0.271	8.84E-09	phosphodiesterase 8B [Source:MGI Symbol;Acc:MGI:2 443 999]
Slc25a21	3.38E-13	−0.273829	0.137	0.282	1.09E-08	solute carrier family 25 (mitochondrial oxodicarboxylate carrier), member 21 [Source:MGI Symbol;Acc:MGI:2 445 059]
Arhgef28	5.58E-13	0.27 161 243	0.441	0.362	1.80E-08	Rho guanine nucleotide exchange factor (GEF) 28 [Source:MGI Symbol;Acc:MGI:1 346 016]
Pla2g7	6.34E-13	0.28 317 997	0.601	0.457	2.05E-08	phospholipase A2, group VII (platelet-activating factor acetylhydrolase, plasma) [Source:MGI Symbol;Acc:MGI:1 351 327]
Prr16	1.04E-12	0.30 343 277	0.307	0.175	3.34E-08	proline rich 16 [Source:MGI Symbol;Acc:MGI:1 918 623]
Spred1	1.39E-12	0.25 156 328	0.463	0.319	4.48E-08	sprouty protein with EVH-1 domain 1, related sequence [Source:MGI Symbol;Acc:MGI:2 150 016]
Atp2b2	1.40E-12	0.25 015 034	0.586	0.427	4.53E-08	ATPase, Ca++ transporting, plasma membrane 2 [Source:MGI Symbol;Acc:MGI:105 368]
Exoc6b	1.53E-12	−0.2506715	0.434	0.524	4.93E-08	exocyst complex component 6B [Source:MGI Symbol;Acc:MGI:1 923 164]
Itpr2	1.56E-12	0.25 815 607	0.49	0.345	5.02E-08	inositol 1,4,5-triphosphate receptor 2 [Source:MGI Symbol;Acc:MGI:99 418]
Serpine2	1.96E-12	0.27 511 277	0.496	0.348	6.32E-08	serine (or cysteine) peptidase inhibitor, clade E, member 2 [Source:MGI Symbol;Acc:MGI:101 780]
Irak2	1.97E-12	0.26 542 063	0.468	0.325	6.36E-08	interleukin-1 receptor-associated kinase 2 [Source:MGI Symbol;Acc:MGI:2 429 603]
Fgf14	2.41E-12	−0.2718435	0.76	0.823	7.79E-08	fibroblast growth factor 14 [Source:MGI Symbol;Acc:MGI:109 189]
2610035D17Rik	3.32E-12	−0.2719188	0.406	0.534	1.07E-07	RIKEN cDNA 2610035D17 gene [Source:MGI Symbol;Acc:MGI:1 919 636]
Gli2	3.49E-12	0.25 442 862	0.465	0.323	1.13E-07	GLI-Kruppel family member GLI2 [Source:MGI Symbol;Acc:MGI:95 728]
Map4	6.20E-12	−0.2509026	0.478	0.561	2.00E-07	microtubule-associated protein 4 [Source:MGI Symbol;Acc:MGI:97 178]
Ptprj	6.24E-12	0.31 704 566	0.43	0.288	2.02E-07	protein tyrosine phosphatase, receptor type, J [Source:MGI Symbol;Acc:MGI:104 574]
Nnat	7.56E-12	−0.3323499	0.171	0.211	2.44E-07	neuronatin [Source:MGI Symbol;Acc:MGI:104 716]
Fut9	8.43E-12	0.28 325 366	0.664	0.564	2.72E-07	fucosyltransferase 9 [Source:MGI Symbol;Acc:MGI:1 330 859]
Cpq	1.19E-11	0.26 741 001	0.524	0.434	3.83E-07	carboxypeptidase Q [Source:MGI Symbol;Acc:MGI:1 889 205]
Pigk	1.20E-11	0.25 920 647	0.263	0.142	3.86E-07	phosphatidylinositol glycan anchor biosynthesis, class K [Source:MGI Symbol;Acc:MGI:1 913 863]
Grid1	1.20E-11	0.25 653 833	0.452	0.309	3.88E-07	glutamate receptor, ionotropic, delta 1 [Source:MGI Symbol;Acc:MGI:95 812]
Plpp3	2.08E-11	0.27 305 668	0.794	0.711	6.72E-07	phospholipid phosphatase 3 [Source:MGI Symbol;Acc:MGI:1 915 166]
Tbc1d5	2.18E-11	−0.2562902	0.396	0.463	7.05E-07	TBC1 domain family, member 5 [Source:MGI Symbol;Acc:MGI:1 919 488]
Znrf3	2.20E-11	0.25 537 248	0.542	0.412	7.12E-07	zinc and ring finger 3 [Source:MGI Symbol;Acc:MGI:3 039 616]
Zfhx3	3.18E-11	−0.2884542	0.367	0.452	1.03E-06	zinc finger homeobox 3 [Source:MGI Symbol;Acc:MGI:99 948]
Hivep3	4.73E-11	0.2 583 213	0.732	0.617	1.53E-06	human immunodeficiency virus type I enhancer binding protein 3 [Source:MGI Symbol;Acc:MGI:106 589]
Nr3c2	5.97E-11	0.28 553 291	0.633	0.521	1.93E-06	nuclear receptor subfamily 3, group C, member 2 [Source:MGI Symbol;Acc:MGI:99 459]
Nav3	6.01E-11	0.32 715 619	0.519	0.369	1.94E-06	neuron navigator 3 [Source:MGI Symbol;Acc:MGI:2 183 703]
Rfx4	6.35E-11	−0.3286631	0.522	0.624	2.05E-06	regulatory factor X, 4 (influences HLA class II expression) [Source:MGI Symbol;Acc:MGI:1 918 387]
Ank	7.92E-11	−0.2564949	0.418	0.493	2.56E-06	progressive ankylosis [Source:MGI Symbol;Acc:MGI:3 045 421]
Rorb	8.58E-11	0.28 096 134	0.863	0.766	2.77E-06	RAR-related orphan receptor beta [Source:MGI Symbol;Acc:MGI:1 343 464]
Paqr8	9.40E-10	0.26 548 741	0.68	0.585	3.03E-05	progestin and adipoQ receptor family member VIII [Source:MGI Symbol;Acc:MGI:1 921 479]
Dlgap1	1.02E-09	0.25 343 148	0.85	0.758	3.28E-05	DLG associated protein 1 [Source:MGI Symbol;Acc:MGI:1 346 065]
Pcdh9	1.62E-09	0.30 264 933	0.992	0.959	5.25E-05	protocadherin 9 [Source:MGI Symbol;Acc:MGI:1 306 801]
Grm3	2.89E-09	0.43 138 154	0.569	0.469	9.32E-05	glutamate receptor, metabotropic 3 [Source:MGI Symbol;Acc:MGI:1 351 340]
Cdh20	1.24E-08	0.27 398 562	0.717	0.65	0.000 399 728	cadherin 20 [Source:MGI Symbol;Acc:MGI:1 346 069]
Fam155a	1.75E-08	−0.449773	0.293	0.396	0.00 056 492	NA
Lhfp	5.61E-08	0.30 326 922	0.56	0.46	0.001 810 805	lipoma HMGIC fusion partner [Source:MGI Symbol;Acc:MGI:1 920 048]

**Figure 5.**
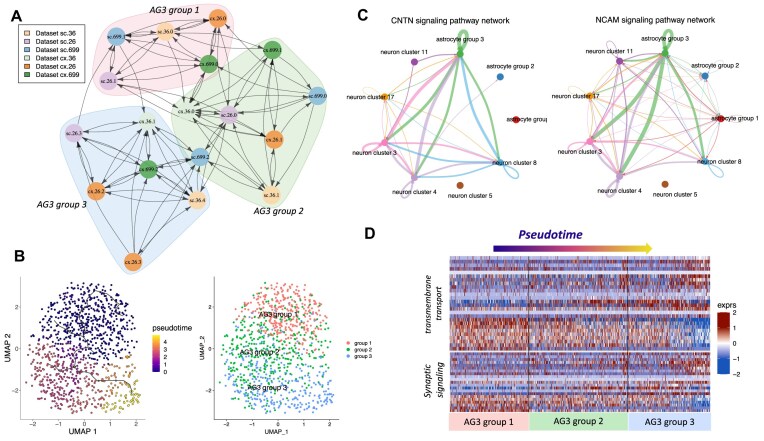
Detailed analysis of AG 3. (**A**) Similarity of clusters within AG3 subpopulation. Community analysis over the graph showed three distinct groups. (**B**) UMAP of AG3 subgroups and the computed pseudotime across these subgroups. Expression data from AG3 from all mice was previously merge and processed. (**C**) Cell-cell signaling pathways CNTN and NEGR with an active role of AG3 with various neuron subclusters. (**D**) Gene expression of relevant biological functions that showed significant differential expression as function of pseudotime.

To interrogate the inner variability of AG3, we performed an individual similarity test using CFS metric by sub-setting the clusters from AG3 cells and performing a community analysis on the resulting graph. Results revealed three distinct sub-groups (Fig. 5A), that once annotated, showed similar sources of variability on the merged data of AG3 cells as a second test (Fig. 5B). We decided to include cluster 2 from sample sc.699 on AG3 group 3 as the number of inward edges was higher from subgroup 3 than from the assigned community group 2. We hypothesized these sub-populations corresponded to transient astrocytic states. To test this, we computed pseudotime scores using Monocle2 [36] for these three distinct groups using the merged data (Fig. 5B). Top genes found differentially expressed as a function of pseudotime were linked by similar terms related with cell junction, cell-cell and synapse signaling, glutamatergic synapse, and transmembrane transport (Fig. 5D). Cell-cell communication analysis using the motor cortex expression data from the tree mice found Contactin (CNTN) and Neural Cell Adhesion Molecule (NCAM) signaling pathways exclusively active between AG3 and neuronal subclusters (Fig. 5C). CNTN family of cell recognition molecules are involved in the maintenance of the nervous system [99]. NCAM mediates neural cell–cell interactions and neurite outgrowth [100] and facilitates the interaction between astrocytes and synapses, modulating neuronal communication [101].

## Discussion

Single cell sequencing represents a powerful approach offering an unprecedent resolution at molecular level. Here we introduce ClusterFoldSimilarity, a novel methodology designed to analyze collections of independent datasets including multimodal, multi-tissue or multi-species experiments, without data integration. Due to its interpretability, CFS enhance reproducibility and comparability of single cell studies, and can be easily integrated into the existing bioinformatics ecosystem. Our novel metric successfully identifies similar cell groups across any number of independent datasets, encompassing different sequencing technologies, achieving similar benchmarking scores at cell type labeling as other state of the art integrative methods, although with a higher accuracy when the number of overlapping features between single cell studies is low. Importantly, it achieves this without the need for an integrative step or batch effect removal over the sequencing signal, both of which can be strongly influenced by noise and inherent partial stochasticity in the data, potentially resulting in the loss of information. It first computes FCs using a Bayesian approach to estimate differential abundance, adapting and expanding previous statistical frameworks, to construct a comparable frame of reference between groups and datasets. The similarity can be understood as a cumulative score across all features, in which concordant FCs in the same vector space point at a similar phenotype. A potential limitation of our method arises when cell-level resolution is needed, as our metric operates at cell-group level, information at individual-cell level is not available. To partially mitigate this, incrementing the granularity and the number of groups could be desirable. Although, we show that our metric captures information related with the heterogeneity of cell groups, and the feature selection ability of our method can help identifying distinct markers of the composing members of the clusters on downstream analysis.

We demonstrate the effective comparison of diverse omics datasets, first by benchmarking cell labeling accuracy using a complex mice gastrulation dataset, and a multi-species pancreatic assay. Secondly, we show how our methodology is capable of matching conserved cell groups across different species, using pancreatic single-cell RNA-seq from human and mouse. In a third example, we expand the multimodality to include single-cell proteomics (CyTOF), bulk-sorted RNA-Seq and single-cell RNA-Seq from synovial tissue of RA and OA patients from B cells, T cells, monocytes, and stromal fibroblast populations. The similarity scores correctly associated cell groups with the same phenotype, even with a small number of common features available (n = 33). An additional multimodal assay was tested employing gene activity scores derived from ATAC-seq and a reference RNA-Seq data, matching populations across peripheral blood mononuclear cells (PBMC) datasets.

Finally, to exemplify a real use case, we sequence and analyze single-nuclei RNA-Seq data obtained from the spinal cord and motor cortex of adult mice, with a focus on exploring astrocytes as a disease-relevant glial cell subpopulation. By utilizing our similarity score, we identify three distinct subpopulations that are present in both tissues, each associated with diverse functionalities: a small population enriched in neurogenesis markers, a medium-size group of activated Gfap-positive astrocytes, and a large group of mature astrocytes with three additional sub-phenotypes. These findings will allow to further explore the roles of different astrocyte types or activation states depending, for example, on age or disease.

We believe that ClusterFoldSimilarity presents a valuable contribution to the ecosystem of bioinformatic tools for single-cell analysis, providing easily interpretable results, and solutions for the construction and exploitation of future cell atlases, which will be indispensable for gaining a comprehensive understanding of cell and tissue heterogeneity.

## Supplementary Material

lqaf042_Supplemental_Files

## Data Availability

The algorithm ClusterFoldSimilariy is implemented in R language and freely available for download from Bioconductor repository (https://www.bioconductor.org/) as an R package (DOI: 10.18129/B9.bioc.ClusterFoldSimilarity), and on GitHub: https://github.com/OscarGVelasco/ClusterFoldSimilarity. Data from the pancreatic single-cell RNA-Seq datasets used for validation are available from Gene Expression Omnibus (GEO) under accession numbers: GSE84133 [102], GSE86469 [103], GSE81608 [104], and ArrayExpress: E-MTAB-5061 [105]. PBMC ATAC-Seq and RNA-Seq data from 10x was downloaded from 10x genomic website: https://www.10xgenomics.com/resources/datasets/10k-human-pbmcs-atac-v2-chromium-controller-2-standard for unimodal ATAC-Seq. CITE-seq atlas of the circulating human immune system, comprising 160k cells with 26 annotated cell types was downloaded from GEO database under the accession number GEO: GSE164378. RA and OA single-cell RNA-seq data, bulk RNA-seq data and mass cytometry data were downloaded from ImmPort (https://www.immport.org/shared/study/SDY998) under study accession code SDY998. CellRanger output and Seurat objects with the processed data, including astrocyte subpopulation annotation, from the mouse single-nuclei RNA-Seq data from spinal cord and cortex generated for this analysis, as well as some of the processed test public datasets, are openly available in Zenodo (doi: 10.5281/zenodo.14843698). All the R script to generate the results, benchmarks and figures can be found on the Zenodo repository: 10.5281/zenodo.15082356.
