# Supplementary material for: Identifying similar populations across independent single cell studies without data integration
Source: NAR Genom Bioinform. 2025 Apr 24;7(2):lqaf042. doi: 10.1093/nargab/lqaf042 (PMC12019640; doi:10.1093/nargab/lqaf042)
Supplement: lqaf042_Supplemental_Files [file lqaf042_supplemental_files.zip › Supplementary Figures 1 2 3 4.pdf]

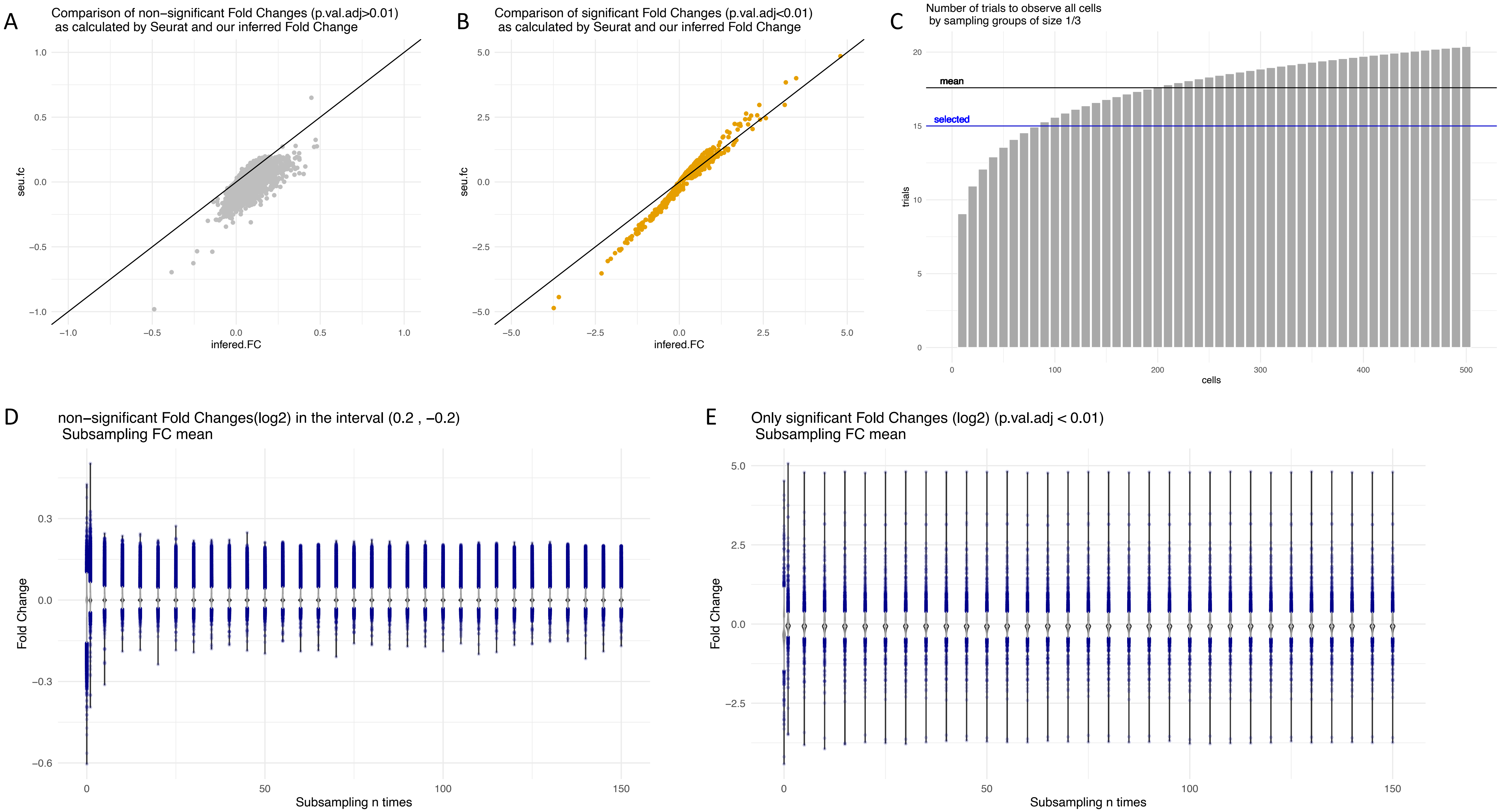
A
Comparison of non−significant Fold Changes (p.val.adj>0.01)
as calculated by Seurat and our inferred Fold Change
seu.fc
infered.FC
B
Comparison of significant Fold Changes (p.val.adj<0.01)
as calculated by Seurat and our inferred Fold Change
seu.fc
infered.FC
C
Number of trials to observe all cells
by sampling groups of size 1/3
mean
selected
trials
cells
D
non−significant Fold Changes(log2) in the interval (0.2 , −0.2)
Subsampling FC mean
Fold Change
Subsampling n times
E
Only significant Fold Changes (log2) (p.val.adj < 0.01)
Subsampling FC mean
Fold Change
Subsampling n times

Supplementary Figure 1. Fold-change subsampling impact metrics using human pancreas alpha cells VS beta cells. ***a*** *Comparison of non-significant fold-change values computed using Seurat and out methodology.* ***b*** *Comparison of significant (p.value.adj<0.01) fold-change values computed using Seurat and out methodology. For significant fold-changes the values are closer to the observed values by Seurat.* ***c*** *average number of subsampling's needed to observe every cell in a group of n. cells.* ***d*** *Distribution of non-significant fold-changes in the range [-0,2, 0.2] across the different subsampling sizes; 0 corresponds to the original observed log2 fold-change.* ***e*** *Distribution of significant (p.val.adj<0.01) fold-changes across the different subsampling sizes; 0 original observed log2 fold-change.*

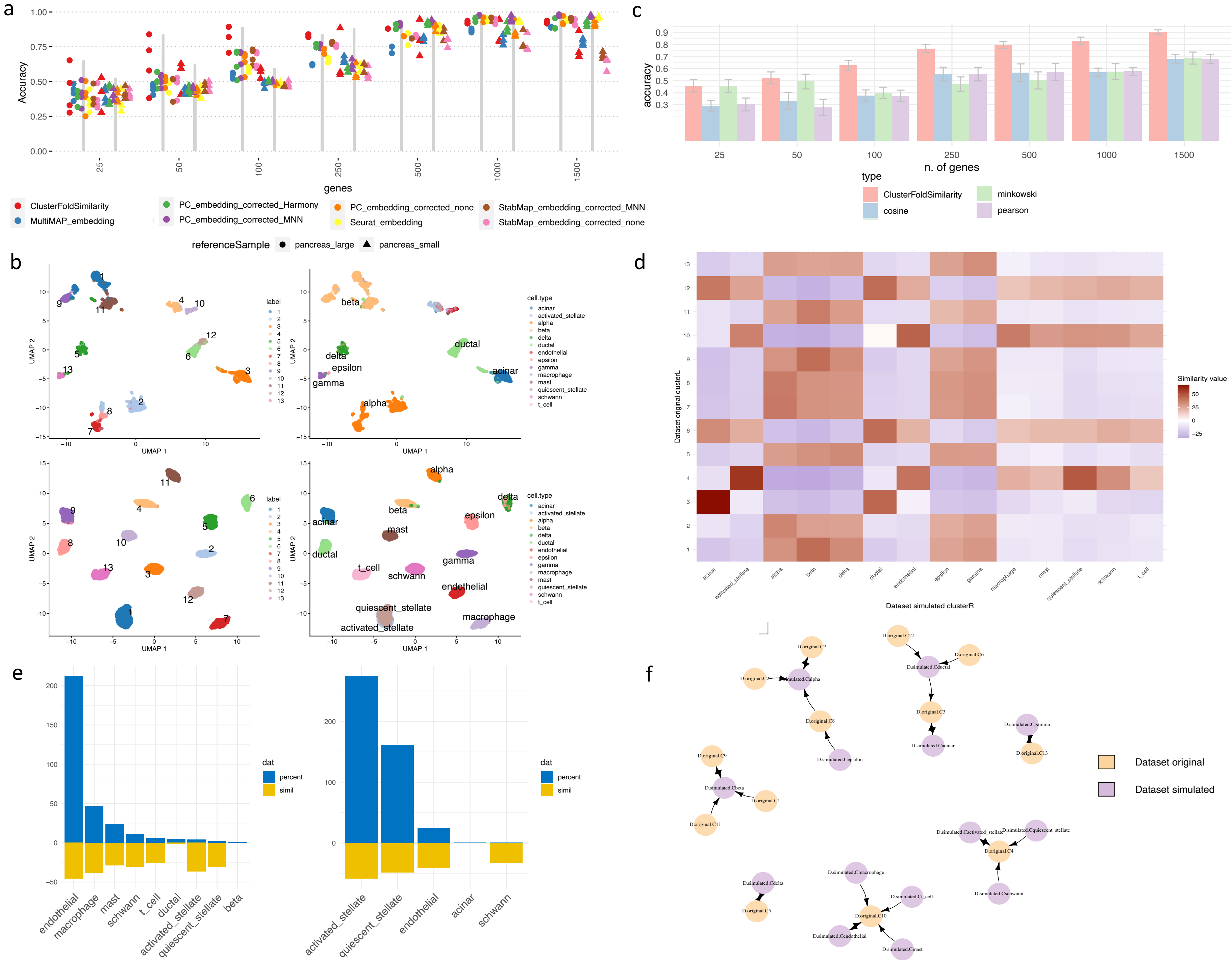
a
Accuracy
genes
25
50
100
250
500
1000
1500
ClusterFoldSimilarity
MultiMAP_embedding
PC_embedding_corrected_Harmony
PC_embedding_corrected_MNN
PC_embedding_corrected_none
Seurat_embedding
StabMap_embedding_corrected_MNN
StabMap_embedding_corrected_none
referenceSample
pancreas_large
pancreas_small
c
accuracy
n. of genes
type
ClusterFoldSimilarity
cosine
minkowski
pearson
b
UMAP 1
UMAP 2
label
cell.type
acinar
activated_stellate
alpha
beta
delta
ductal
endothelial
epsilon
gamma
macrophage
mast
quiescent_stellate
schwann
t_cell
d
Dataset original clusterL
Dataset simulated clusterR
Similarity value
e
dat
percent
simil
endothelial
macrophage
mast
schwann
t_cell
ductal
activated_stellate
quiescent_stellate
beta
acinar
f
Dataset original
Dataset simulated

*Supplementary Figure 2. **a** Plot showing cell type classification accuracy benchmark comparison against integrative methods (MultiMap; naive PCA, PCA MNN corrected, PCA Harmony corrected, Seurat CCA, StabMap, StabMap MNN corrected) using two pancreatic scRNA-Seq datasets with varying sizes. **b** Barplot showing obtained accuracies from the benchmark comparing CFS to the Pearson distance, cosine similarity, and Minkowski distance using pancreas scRNA-Seq. **c** UMAP dimensionality reduction of human pancreas Dataset 1 from Baron et. al (top) and simulated dataset (below) showing the cluster labels (left) and annotated cell type label (right). **d** Heatmap showing all the computed similarity scores between datasets. **e** Barplots showing the number of cells (blue) versus the similarity value for the cell type (yellow). Left: results for Cluster 10, right: results for Cluster 4. **f** Directed graph of clusters by similarity, where each node corresponds with a cluster/cell type group from one dataset (dataset original: dataset from Baron et. al, simulated: dataset simulated from the original using scDesign2).*

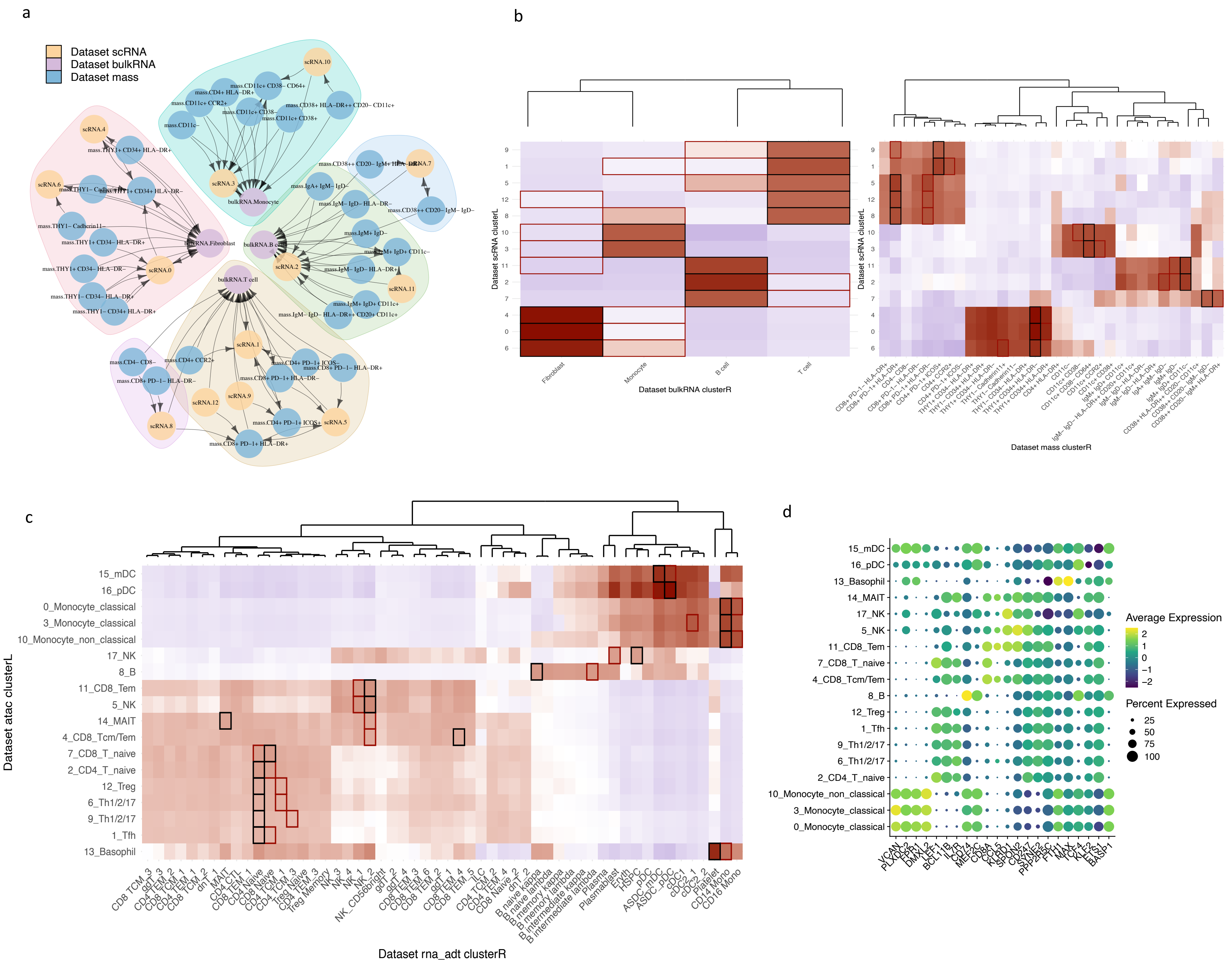
a
Dataset scRNA
Dataset bulkRNA
Dataset mass
b
Dataset scRNA clusterL
Fibroblast
Monocyte
B cell
T cell
Dataset bulkRNA clusterR
Dataset mass clusterR
c
15_mDC
16_pDC
0_Monocyte_classical
3_Monocyte_classical
10_Monocyte_non_classical
17_NK
8_B
11_CD8_Tem
5_NK
14_MAIT
4_CD8_Tcm/Tem
7_CD8_T_naive
2_CD4_T_naive
12_Treg
6_Th1/2/17
9_Th1/2/17
1_Tfh
13_Basophil
Dataset atac clusterL
Dataset rna_adt clusterR
d
15_mDC
16_pDC
13_Basophil
14_MAIT
17_NK
5_NK
11_CD8_Tem
7_CD8_T_naive
4_CD8_Tcm/Tem
8_B
12_Treg
1_Tfh
9_Th1/2/17
6_Th1/2/17
2_CD4_T_naive
10_Monocyte_non_classical
3_Monocyte_classical
0_Monocyte_classical
Average Expression
2
1
0
−1
−2
Percent Expressed
25
50
75
100

Supplementary Figure 3. a. Directed graph of top similarity values between clusters and cell groups from single cell RNA-seq, sorted bulk RNA-seq and Cytof samples from rheumatoid arthritis (RA) and osteoarthritis (OA) from sorted B cells, T cells, monocytes, and stromal fibroblast populations (single cell RNA-Seq fibroblast clusters 0, 4, 6; T cell clusters 1, 5, 8, 9, 12; B cells clusters: 2, 7, 11; monocyte clusters: 3, 10) b. Heatmap of all similarity values from single cell RNA-Seq clusters and bulk RNA-Seq and Cytof samples. c. Heatmap showing all computed similarity scores between the single cell ATAC-Seq gene activity scores and the RNA-Seq annotated dataset. Black squares highlight the top value of similarity, dark red squares the top second similarity value. d. Selected markers by ClusterFoldSimilarity for clusters of the ATAC-Seq data.

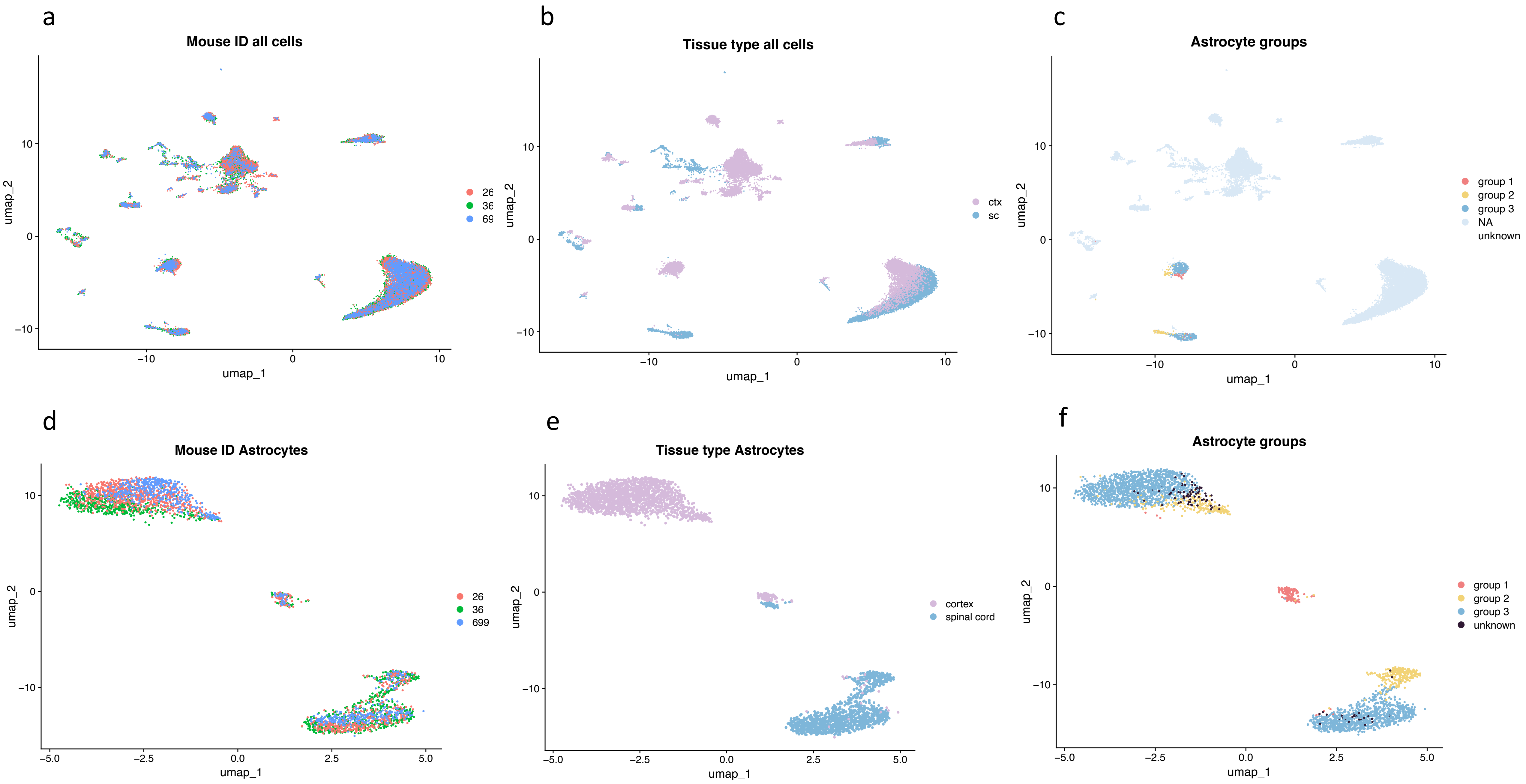
a
Mouse ID all cells
umap_2
umap_1
26
36
69
b
Tissue type all cells
umap_2
umap_1
ctx
sc
c
Astrocyte groups
umap_2
umap_1
group 1
group 2
group 3
NA
unknown
d
Mouse ID Astrocytes
umap_2
umap_1
26
36
699
e
Tissue type Astrocytes
umap_2
umap_1
cortex
spinal cord
f
Astrocyte groups
umap_2
umap_1
group 1
group 2
group 3
unknown

Supplementary Figure 4. Integrated single-nuclei RNA-Seq data from spinal cord and motor cortex from the three mice. a. UMAP plot showing all cells by mice id. b. UMAP plot showing all cells by tissue id. c. UMAP plot showing astrocyte groups detected by ClusterFoldSimilarity in the context of all cells. d. UMAP plot showing astrocytes by mice id. e. UMAP plot showing astrocytes by tissue id. f. UMAP plot showing astrocytes sub-populations detected by ClusterFoldSimilarity.
